# Capsaicin Interaction with TRPV1 Channels in a Lipid Bilayer: Molecular Dynamics Simulation

**DOI:** 10.1016/j.bpj.2015.02.013

**Published:** 2015-03-24

**Authors:** Sonya M. Hanson, Simon Newstead, Kenton J. Swartz, Mark S.P. Sansom

**Affiliations:** 1 Department of Biochemistry, University of Oxford, Oxford, United Kingdom; 2 Computational Biology Program, Memorial Sloan-Kettering Cancer Center, New York, New York; 3 Molecular Physiology and Biophysics Section, Porter Neuroscience Research Center, National Institute of Neurological Disorders and Stroke, National Institutes of Health, Bethesda, Maryland

## Abstract

Transient receptor potential vanilloid subtype 1 (TRPV1) is a heat-sensitive ion channel also involved in pain sensation, and is the receptor for capsaicin, the active ingredient of hot chili peppers. The recent structures of TRPV1 revealed putative ligand density within the S1 to S4 voltage-sensor-like domain of the protein. However, questions remain regarding the dynamic role of the lipid bilayer in ligand binding to TRPV1. Molecular dynamics simulations were used to explore behavior of capsaicin in a 1-palmitoyl-2-oleoyl phosphatidylcholine bilayer and with the target S1–S4 transmembrane helices of TRPV1. Equilibrium simulations reveal a preferred interfacial localization for capsaicin. We also observed a capsaicin molecule flipping from the extracellular to the intracellular leaflet, and subsequently able to access the intracellular TRPV1 binding site. Calculation of the potential of mean force (i.e., free energy profile) of capsaicin along the bilayer normal confirms that it prefers an interfacial localization. The free energy profile indicates that there is a nontrivial but surmountable barrier to the flipping of capsaicin between opposing leaflets of the bilayer. Molecular dynamics of the S1–S4 transmembrane helices of the TRPV1 in a lipid bilayer confirm that Y511, known to be crucial to capsaicin binding, has a distribution along the bilayer normal similar to that of the aromatic group of capsaicin. Simulations were conducted of the TRPV1 S1–S4 transmembrane helices in the presence of capsaicin placed in the aqueous phase, in the lipid, or docked to the protein. No stable interaction between ligand and protein was seen for simulations initiated with capsaicin in the bilayer. However, interactions were seen between TRPV1 and capsaicin starting from the cytosolic aqueous phase, and capsaicin remained stable in the majority of simulations from the docked pose. We discuss the significance of capsaicin flipping from the extracellular to the intracellular leaflet and mechanisms of binding site access by capsaicin.

## Introduction

**Figure 1:**
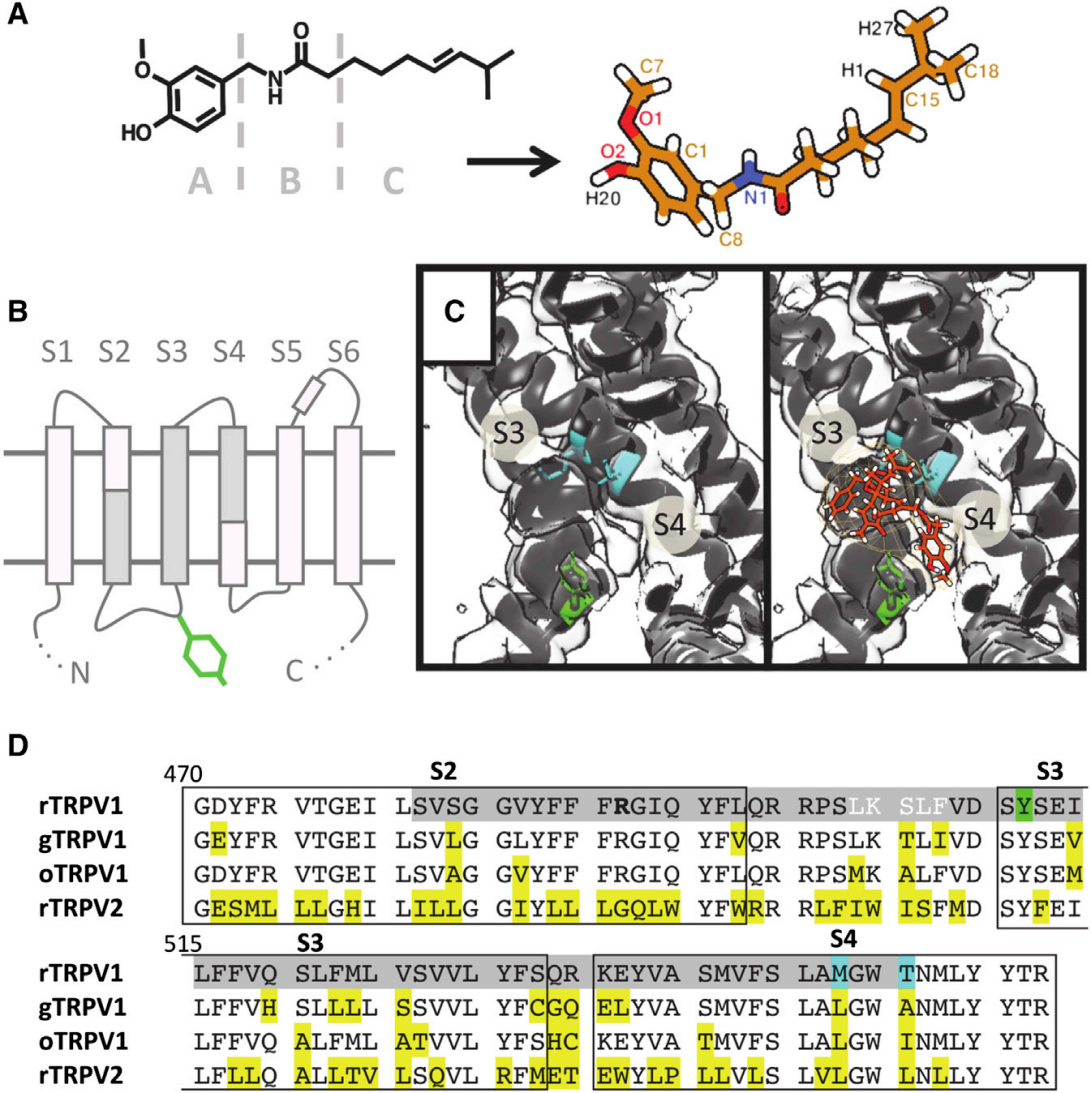
Capsaicin and TRPV1. (*A*) Capsaicin is a hydrophobic molecule that may be divided into three functional groups: the A group aromatic head with hydrogen bonding potentiality, the B group dipolar amide-bond region, and the C group hydrophobic tail. (*B*) The TM region of TRPV1 is composed of six TM helices, S1–S6, of which S5 and S6 form the pore domain, and the S2–S4 helices (*in gray*) are predicted to contain the capsaicin binding site, including the tyrosine 511, indicated in green. (*C*) A cryo-EM structure (PDB ID: 3J5Q) solved with both RTX and DkTx bound, shows density near the expected RTX and capsaicin-binding site (14). Residues known to be significant in RTX binding are colored as in (*D*). (*D*) Sequence alignment of rat (r), chicken (g), and rabbit (o) TRPV1 and rat (r) TRPV2, highlighting in gray the region thought to be physiologically significant (11,12). The tyrosine 511 is highlighted in green. M547 and T550 are also highlighted in blue as significant to ligand binding in TRPV1 (13). The region of the disordered S2–S3 loop is shown in white. Differences from the rat sequences are highlighted yellow. To see this figure in color, go online.

Transient receptor potential (TRP) channels are a diverse set of nonselective cation channels found in eukaryotic cells. These channels are often involved in sensory transduction, participating in the direct detection of stimuli ranging from osmotic sensing to temperature change, and are crucial members of lipid-based signaling pathways (1,2). Among the diversity of TRP channels are the receptors for molecules such as menthol, camphor, mustard, and capsaicin, the latter being responsible for the pungent hot nature of chili peppers (3). Capsaicin specifically activates the heat-sensitive transient receptor potential vanilloid subtype 1 (TRPV1) ion channel (4). Along with other members of the TRP channel family, known to play key roles in temperature sensation and other sensory functions (5), TRPV1 is a prominent therapeutic target (6,7). As capsaicin is a lipophilic molecule (Fig. 1 *A*) and the TRPV1 channel is an integral membrane protein with an overall transmembrane (TM) architecture resembling that of voltage-gated potassium (Kv) channels (8), it is likely that interactions with the membrane environment play a key role in mediating the effect of capsaicin. It is therefore important to understand those interactions in molecular detail.

Application of capsaicin to sensory neurons increases membrane conductance to cations, and was a valuable tool to early studies of nociceptive neurons (9). The capsaicin molecule may be divided into three functional groups: (A) the substituted aromatic region or vanillyl group, (B) the dipolar amide-bond region, and (C) the hydrophobic tail region (Fig. 1 *A*). The effects of modifications to each of these groups was well explored by the early nineties, for example, removal of the 4′-OH in the A group removes capsaicin activity, whereas the most important characteristic of the C group acyl chain tail is its hydrophobicity as it tolerates variations from as few as five carbons to as many as 15 without extensively modifying activity (10). Although these studies gave clues regarding tolerated modifications to capsaicin, the hydrophobicity of the capsaicin molecule and its derivatives make quantitative direct binding studies to the TM TRPV1 protein difficult. As a result resiniferatoxin (RTX), a compound from Moroccan cactus with higher affinity to TRPV1, has been used to perform radioligand binding experiments (11).

TRP channels belong to the tetrameric 6-TM superfamily of ion channels, which are composed of the pore-forming S5–S6 TM helices and an outer domain formed by the S1–S4 TM helices, which constitute the voltage-sensing domain (VSD) in canonical voltage-gated channels (Fig. 1 *B*). The VSD-like domain of TRP channels does not contain the positively charged amino acids in the S4 helix characteristic of voltage-sensitive channels. However, mutagenesis and related studies have revealed that residues found crucial to capsaicin and RTX binding are within the S2–S4 helices of TRPV1 (12–14) (see Fig. 1 *D*), thus implicating the VSD-like domain in the overall activation mechanism of the channel. In particular, mutating a tyrosine residue on the cytoplasmic side of the S3 helix (Y511) (Fig. 1 *B*) to alanine removes capsaicin sensitivity while the channel retains sensitivity to pH and heat (12). Channel activation by the endogenous ligand anandamide, released during tissue injury, is also lost in this mutant. Several mutagenesis studies have confirmed Y511’s role in vanilloid binding to TRPV1, and also identified residues crucial to RTX binding in the middle of the S4 helix (13) (see Fig. 1 *D*).

Recently, the structure of rat TRPV1 at 3.4 Å resolution has confirmed that these residues lie within the lipid bilayer (Fig. 1 *C*), in line with the suggestion of a TM-binding site for TRPV1 ligands (8). Structures of TRPV1 with agonists bound (14) revealed both changes in the central pore domain upon ligand application as well as the location of the bound agonist molecules. Although the electron density attributed to RTX and to capsaicin near the S2–S4 helices was not sufficiently detailed to resolve their exact orientation and interactions, the RTX density was consistent with an interpretation that its equivalent to the A group of capsaicin interacts with the Y511 near the water-bilayer interface on the inner (i.e., cytosolic) side of the membrane (Fig. 1 *C*) (14).

Capsaicin is a relatively hydrophobic compound and a number of biophysical studies have revealed effects of capsaicin on model lipid bilayers ranging from pore formation (15), to effecting the phase transition temperature of lipids (16), to increasing bilayer fluidity (17). Additionally, a number of studies have highlighted the experimental issues associated with exploring the mode of action of hydrophobic compounds. For example, one study showed threefold higher potency upon longer preincubation with the ligand (18) and another identified a positive correlation between lipid partition coefficients and the pungency of TRPV1 agonists (19). Furthermore, there remains a lack of clarity as to whether capsaicin acts via the outer (i.e., extracellular) or inner (i.e., cytosolic) side of the cell membrane. Although capsaicin and RTX can both activate the channel when applied from either side of the membrane, a charged analog of capsaicin only activates TRPV1 when applied intracellularly (20). Several other experiments, however, have also approached this question with conflicting results (20–24).

The surprisingly diverse consequences of the incomplete understanding of the role of the lipid bilayer in the interaction of capsaicin and its analogs with TRPV1 lead to a need for a higher resolution picture of the three elements of the system. Molecular dynamics (MD) simulation studies can reveal details of the interactions between hydrophobic molecules and lipid bilayers (25–34), and between lipids and integral membrane proteins (35–37). Previous relevant studies of the TRPV1-capsaicin interaction include simulations of capsaicin in an octanol-water system (38), as well as a docking study of capsaicin to a TRPV1 homology model (39). Neither of these studies directly addresses the role of the phospholipid bilayer in the interaction of capsaicin and TRPV1. However, simulation studies of other lipid-like molecules have explored their interactions with a lipid bilayer, addressing the issue of flip-flop from one bilayer leaflet to the other (40–42). These studies provide a possible protocol for using simulations to study how externally applied capsaicin reaches a cytosolic-facing binding site.

In this work, we explore the mechanistic importance of the lipid bilayer in ligand binding to TRPV1. By combining submicrosecond simulations of capsaicin in a phospholipid bilayer, both in the absence and the presence of the S1–S4 domain of TRPV1, with calculations of the free energy landscape for capsaicin translation along the bilayer normal, we arrive at a plausible model of how capsaicin accesses its binding site on the cytosolic face of the cell membrane. We find that the distribution of capsaicin in a lipid bilayer aligns the aromatic A group with the carbonyls of the lipid, consistent with the location of the comparable functional group of RTX in recent structural studies. Additionally, we propose the significance of capsaicin flip-flop from the extracellular to the intracellular side of the bilayer to access its binding site on TRPV1.

## Materials and Methods

Simulations of capsaicin with a 1-palmitoyl-2-oleoyl phosphatidylcholine (POPC) bilayer with or without protein were performed and analyzed using the GROMACS 4.5.5 (www.gromacs.org) (43) using the OPLS force field (44). Each simulation contained four capsaicin molecules, corresponding to 13 mM capsaicin. For each simulation the capsaicin molecules were initially placed at the center of each of the four *x*-*y* quadrants of the simulation box, either in the aqueous phase at a distance of 30 Å from the center of the bilayer, or at the center of the bilayer. To introduce the capsaicin molecule to the system, a slow-growth approach was used to transform a noninteracting molecule to a fully interacting molecule over a period of 0.5 ns. After this setup procedure 2 × 100 ns and 1 × 500 ns simulations were conducted with capsaicin in the aqueous starting position, and 1 × 500 ns simulation were conducted with capsaicin in the lipid starting position, each simulation containing four capsaicin molecules (see Table 1). A control POPC bilayer with no capsaicin molecules present was simulated for 500 ns.

Each simulation box contained 256 POPC molecules to form the bilayer, which was initially created using coarse-grained self-assembly methods (45). This bilayer was then converted to an atomistic representation. The final simulation box was of dimensions ∼93 Å × 93 Å × 99 Å and contained 16893 TIP4P waters and NaCl at a concentration of ∼230 mM. The system was maintained at a constant temperature of 323 K and a constant pressure of 1 atm using semiisotropic coupling with the Berendsen algorithm (46). Capsaicin was parameterized in the OPLS all-atom force field using topolbuild version 1.3 for initial parameterization along with an in-house script top-fill. The similarity of functional group in capsaicin to those present in peptides meant no subsequent manual curation was necessary (see the Supporting Material for the parameters used).

Umbrella sampling simulations employed the *z* axis (as an approximation to the bilayer normal) as the reaction coordinate, with windows spaced at 1 Å intervals. These simulations were conducted using GROMACS 4.6.1. An umbrella potential using a force constant of 1000 kJ mol^−1^ nm^−2^ was applied to the center of mass of the capsaicin molecule using the pull geometry cylinder method (27). Production simulations were run for 10 ns/window, of which the last 5 ns were used to generate a potential of mean force (PMF) profile using the weighted histogram analysis method (47). The profile was symmetrized and errors were calculated by the bootstrapping method (48).

Simulations including protein were conducted using a similar setup to those without protein, but including the S1–S4 helix domain of the apo structure of TRPV1 (Protein Data Bank (PDB) ID: 3J5P; 3.4 Å resolution) (8). The unresolved loop between the S2 and S3 helices (represented in *white lettering in the sequence alignment* in Fig. 1 *C*) was modeled in using Modeler version 9.10 (49). For simulations in which capsaicin was added to the aqueous phase, the S1–S4 TM region was initially embedded into the bilayer using a coarse-grained self-assembly approach, and subsequently converted into an atomistic representation. In a separate set of simulations, the g_membed (50) tool was used to insert the protein into a bilayer already containing four capsaicin molecules in the intracellular leaflet. Overall 4 × 50 ns and 2 × 100 ns simulations each were conducted to explore the capsaicin-S1–S4 interaction from the aqueous and lipid phase, and 6 × 50 ns simulations were conducted to explore possible interactions of the capsaicin molecules already on the intracellular leaflet with the TRPV1 S1-S4 helices. Three 100 ns simulations were also run of just the S1–S4 protein in the membrane without any capsaicin (see Table 1).

To further investigate the interaction between capsaicin and TRPV1 within the context of an explicit bilayer, simulations were conducted starting with capsaicin in a docked pose on TRPV1 S1–S4. Docking in the absence of a bilayer was performed with AutoDock Vina (51) on all three full-length TRPV1 structures (PDB ID: 3J5P, 3J5R, and 3J5Q). Simulations, however, were only started from the top-ranked capsaicin pose docked to 3J5R, as this was deemed the most physiological starting point. The docked capsaicin and the S1–S4 helices of 3J5R were inserted into a POPC membrane using the g_membed tool and 3 × 50 ns simulations were run. Analysis of these simulations was conducted using the gromacs clustering algorithm (52) and the volmap analysis tool in VMD. Images were generated using PyMOL (53), VMD (54), and Chimera (55).

## Results

### Capsaicin localization

**Figure 2:**
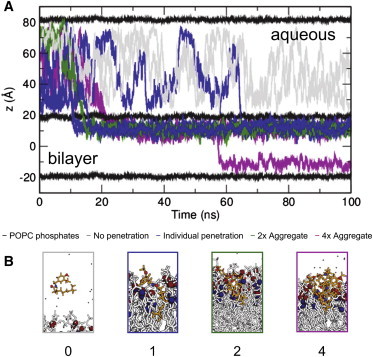
Overview of simulations of capsaicin in the presence of a POPC bilayer. (*A*) Capsaicin molecules penetrated the lipid bilayer spontaneously, though with differing pathways as shown in this plot of the center of mass of individual capsaicin molecules: molecules that inserted individually are shown in blue, molecules that inserted as aggregates of two are shown in green, and molecules that inserted as aggregates of four are shown in magenta. Molecules that remained in the aqueous phase are shown in gray. Each line represents an individual capsaicin molecule. The horizontal black traces correspond to the positions of the lipid bilayer phosphates. The topmost black line and the bottommost correspond to the same lipid bilayer phosphates, due to periodic boundary conditions. Note that the capsaicin molecules represented in this plot are from three separate simulations, and not all capsaicin molecules entered the bilayer from the same side, but have here been superposed as such for ease of conceptualization. (*B*) Snapshots of capsaicin and bilayer for failure to penetrate (0; *gray*), for an individually inserted molecule (1; *blue*), and for insertion as an aggregate of 2 (2; *green*), or 4 (4; *magenta*). To see this figure in color, go online.

Capsaicin is relatively lipophilic as evidenced by an octanol-water partition coefficient of ∼3.8, just above that of *n*-propylbenzene (56,57). In three simulations starting with four capsaicin molecules in the aqueous solution adjacent to a phospholipid bilayer (see Table 1), spontaneous insertion of most of the capsaicin molecules (83%) into the lipid bilayer occurred within the first 100 ns of simulation (Fig. 2). In some cases (four) individual capsaicin molecules are inserted into the bilayer, although in other cases either two (one instance with two capsaicin molecules) or four (one instance with four capsaicin molecules) capsaicin molecules formed an aggregate in the aqueous phase before insertion. However, even when the capsaicin molecules are inserted as an aggregate the capsaicin molecules subsequently disaggregated (laterally) following insertion into the bilayer. In Fig. 2 the trajectories along the bilayer normal of the individual capsaicin molecules from these simulations are shown.

**Figure 3:**
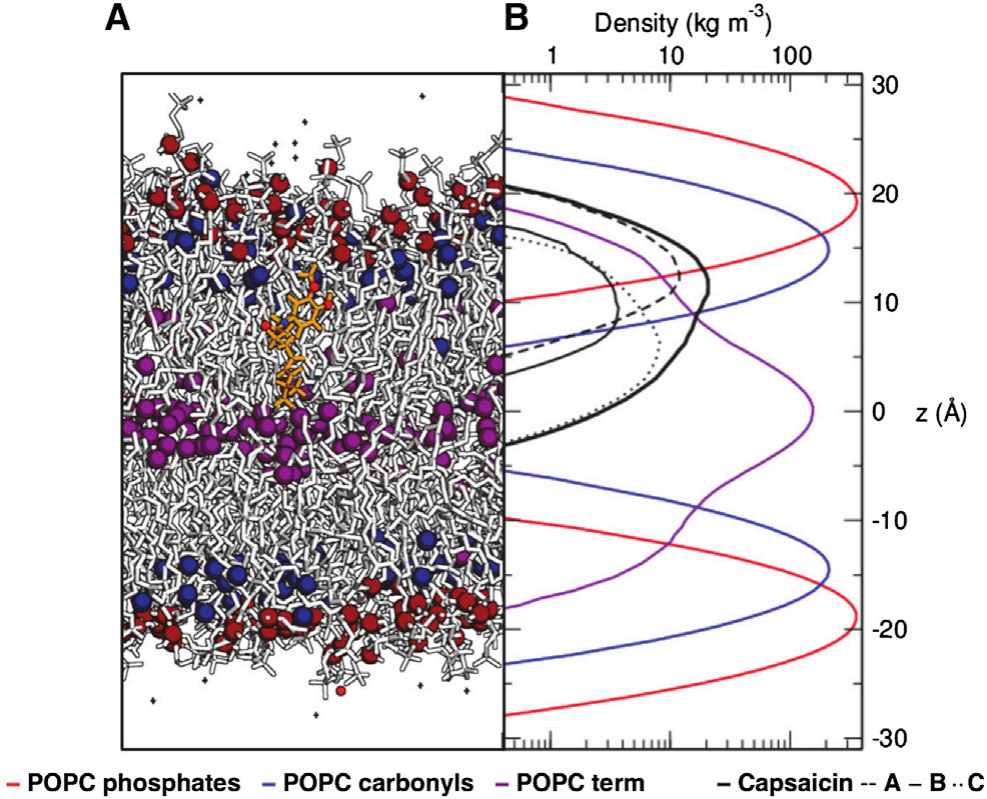
Localization of capsaicin within a model bilayer. During the course of simulations the location of capsaicin remained consistent (*A*) once it penetrated the bilayer. Here, a partial density profile (*B*) of 30 ns of simulation shows how capsaicin sits within a POPC bilayer. POPC phosphates, carbonyls, and terminal methyls are shown in red, blue, and purple, respectively. The capsaicin molecule is shown as a thick black line, and the distribution of the A, B, and C groups are shown as dashed, straight, and dotted lines, respectively. The A-group containing the phenol moiety aligns, on average, with the carbonyls of the POPC, whereas the rest of the molecule extends toward the center of the bilayer. To see this figure in color, go online.

Capsaicin molecules that formed aggregates before insertion subsequently diffused away from each other laterally within the bilayer. Their localization along the bilayer normal remained consistent through the remainder of the simulations, and was not distinguishable from that of capsaicin, which had inserted as individual (i.e., not aggregated) capsaicin molecules. The density distributions of capsaicin and of its A, B, and C groups along the bilayer normal can be compared with the location of the lipid headgroups and tails (Fig. 3). These density profiles show that the vanillyl group (A-region) interacts predominantly with the carbonyls of the lipid molecules. This is comparable to previously seen interactions of amphipathic aromatic (i.e., tyrosine and tryptophan) amino acid side chains of membrane proteins with these groups of lipids (30). An example of a long-lasting (>20 ns) H-bond interaction between a phospholipid carbonyl and capsaicin is shown in the Fig. S1.

**Figure 4:**
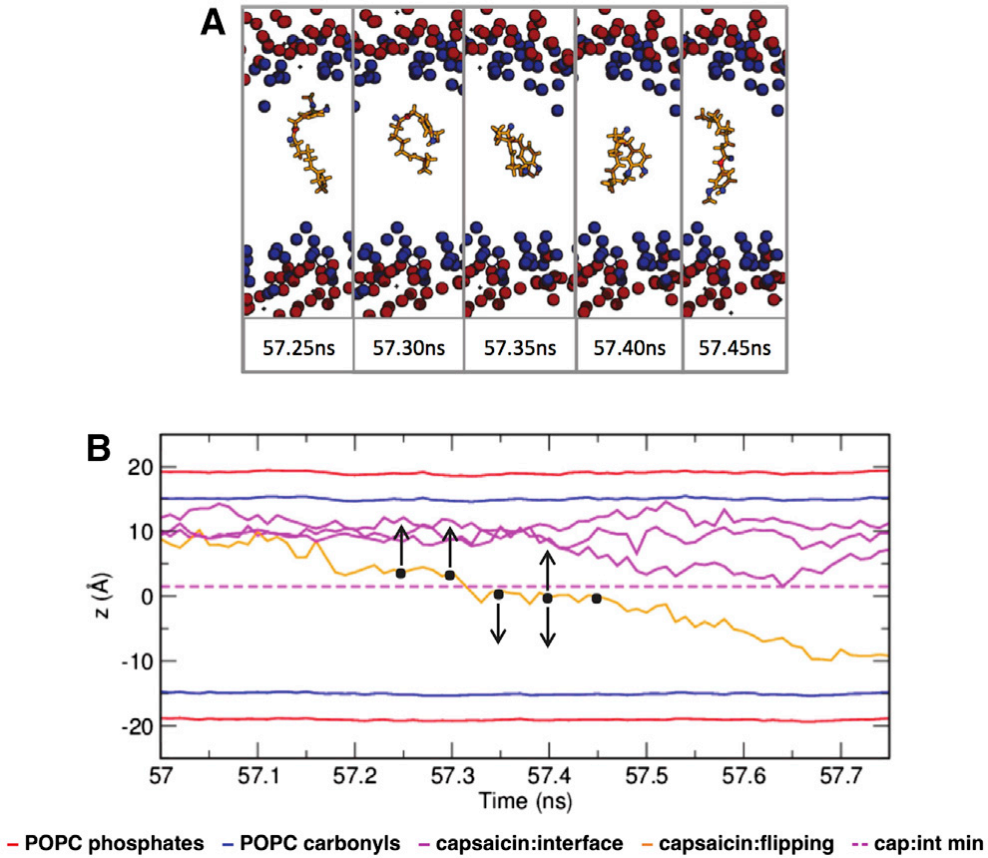
Capsaicin was seen to flip spontaneously from one bilayer to the other. (*A*) Snapshots of the flip-flop event. (*B*) Distance of the center of mass of a capsaicin molecule relative to the bilayer center versus time providing a more detailed view of the single capsaicin that flips from one bilayer to the other. POPC phosphates are shown in red, whereas carbonyls are shown in blue. Capsaicin molecules that did not flip are shown in magenta, with their closest approach to the center of the bilayer shown as a dotted magenta line. Snapshots of the flipping capsaicin were taken at either side of this line, indicated by black dots. The arrows indicate the directions of movement capsaicin in additional simulations (re)started at different time points along this transition (see main text and Fig. S2 for details). To see this figure in color, go online.

Inspection of Fig. 2 reveals that one capsaicin molecule, originally in a 4× aggregate formed in the aqueous phase before insertion (shown by the *magenta line*), flips from one leaflet of the bilayer to the other. To explore possible capsaicin flip-flops further, two simulations of four capsaicin molecules each were extended to 500 ns, resulting in a total of over 4 *μ*s of individual capsaicin trajectories in a lipid bilayer. A similar event, however, did not reoccur. In Fig. 4 *A* the observed flip is shown in atomistic detail via snapshots that represent the molecule before and after the flip-flop transition (Fig. 4 *B*). Additional simulations (re)started at different time points along this transition (see *vertical arrows* in Fig. 4 *B*) suggest that it may correspond to passage over a local maximum (i.e., barrier) in a free energy landscape in that the capsaicin molecule could either flop back to its initial (i.e., extracellular) leaflet location or flip to its new (i.e., cytosolic) location (see Fig. S2 for the trajectories of capsaicin in these (re)started simulations). The presence of a local energy barrier would also explain the low frequency (once in 4 *μ*s total of simulation) with which such a flip was observed.

### Free energy profile

**Figure 5:**
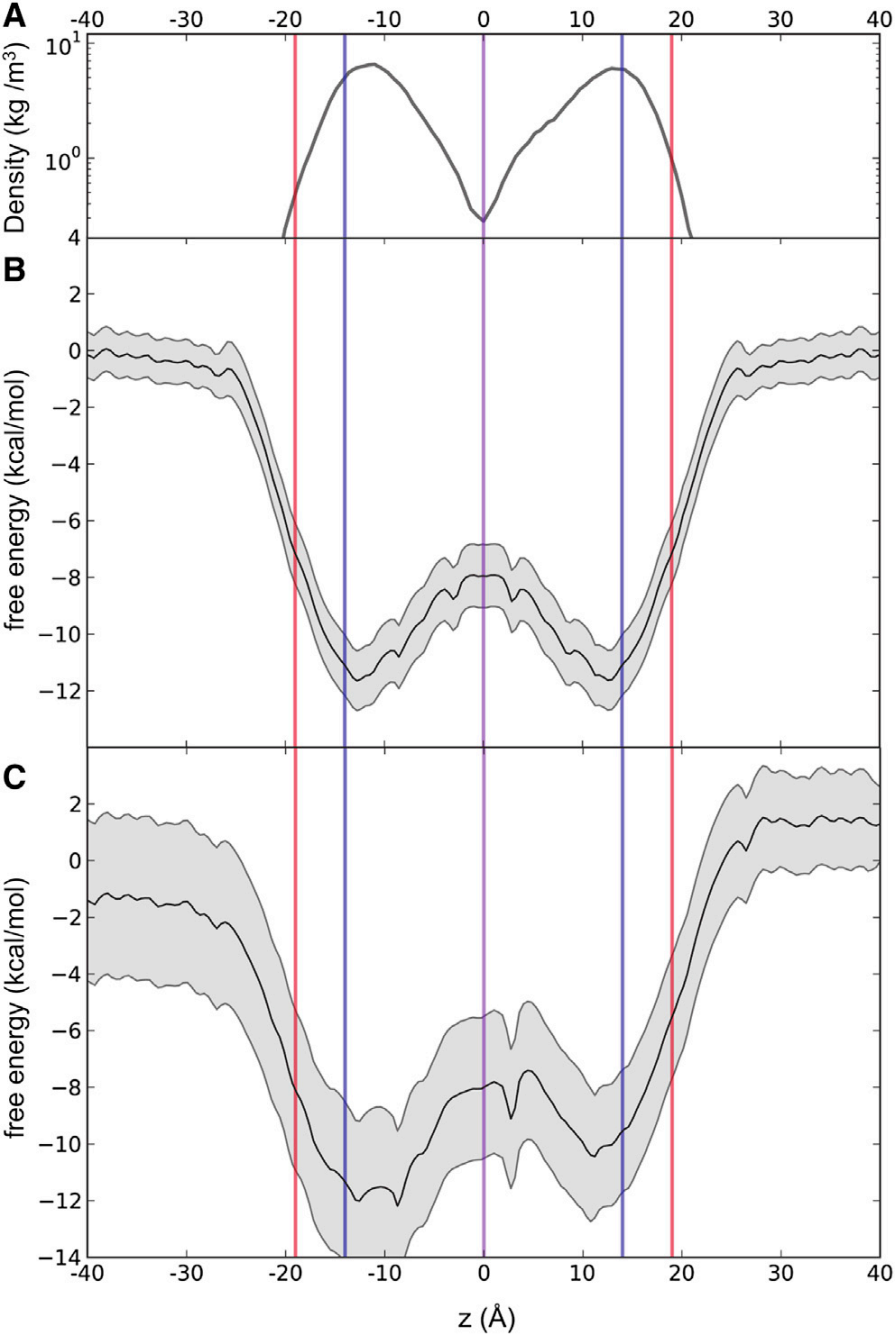
Potential of mean force of capsaicin along the normal of a POPC bilayer. (*A*) The partial density averaged over 50 ns for two capsaicin molecules, initially positioned one in each leaflet of the bilayer (see Fig. 2) compared to the (*B*) symmetrized and (*C*) unsymmetrized PMF profiles, as calculated from the last 5 ns of each 10 ns umbrella restrained simulations at 1 Å windows along the *z* axis, with the free energy set to zero in the aqueous phase. The gray region either side of the PMFs represents the standard deviation on either side of the average profile as calculated by bootstrapping. Vertical lines indicate the mean positions along *z* of the phosphates (*red*), carbonyls (*blue*), and terminal methyls (*purple*) of the lipid molecules. To see this figure in color, go online.

To provide a more quantitative description of the energy landscape underlying capsaicin movement across a lipid bilayer we have calculated the PMF for translation of a capsaicin molecule along the *z* axis, i.e., along the approximate bilayer normal. The PMF for capsaicin crossing a POPC bilayer was obtained by umbrella sampling using 10 ns restrained simulations for 1 Å windows along *z*. Thus, the resulting PMF provides a free energy profile for translation of a single capsaicin molecule from one side to the other of the bilayer (Fig. 5). This profile (see Fig. 5 *B*) is in good agreement with the distribution of capsaicin molecules seen in the earlier standard MD simulations (Fig. 5 *A*).

It is informative to consider errors in the estimates of the PMF. To this end one may compare the symmetrized (Fig. 5 *B*) and unsymmetrized (Fig. 5 *C*) PMFs. Both reveal a similar free energy profile, with energy minima at the water/lipid headgroup interface, and a modest energy barrier at the center of the bilayer. An approximate estimate of the height of the barrier relative to the minima with capsaicin at the preferred interfacial location may be obtained from the relative densities in those two regions, and compared to the estimates from the two PMFs. This gives estimates of the free energy barrier at the center of the bilayer of ∼6 kT from the symmetrized PMF (Fig. 5 *B*), of ∼3 to ∼7 kT from the unsymmetrized PMF (Fig. 5 *C*), and of ∼3 kT from the density profile (Fig. 5 *A*).

The resulting free energy profile also matches well with previous calculations of related PMFs, such as of those previously calculated for the amino acids tyrosine and leucine (30). Because there is no barrier to bilayer insertion and a small but nontrivial barrier (∼6 kT) for crossing the hydrophobic core, it is in agreement with the qualitative results of the unrestrained simulations discussed previously, and provides a more quantitative view of barriers capsaicin faces in accessing the potential TRPV1 binding site on the cytosolic face of the cell membrane.

Capsaicin, with a 10-carbon acyl chain tail (the *C-group* in Fig. 1 *A*), is a relatively large compound with which to conduct free energy calculations across a lipid bilayer. We therefore had some concerns as to whether 10 ns for each simulation window in the umbrella sampling calculations would be sufficient to provide a reasonable, albeit approximate, estimate of the underlying PMF. We were encouraged that a consistent profile indicative of convergent behavior was seen when comparing PMF profiles calculated from subsections of the trajectories (see e.g., Fig. S3). We were also encouraged by the close correspondence between the PMF profile and the distribution of capsaicin molecules from standard MD simulations (Fig. 5 *A*). Examination of an unsymmetrized PMF (Fig. 5 *C*) suggested an error of ∼1 kcal/mol in estimation of well depths and barrier heights. Thus, the depth of the energy well at the bilayer interface relative to the reference bulk aqueous phase was −10 kcal/mol for z = −12 Å and −11.5 kcal/mol for z = +12 Å in the unsymmetrized PMF, compared with −11 kcal/mol for |z| = 12 Å in the symmetrized PMF (Fig. 5). However, we are aware that a number of studies have emphasized the importance of running extended simulations and multiple replicas to achieve a fully converged PMF. We note that several of these studies focus on molecules (e.g., antimicrobial peptides) that significantly perturb the bilayer (27,58,59). In contrast, capsaicin (a relatively lipophilic compound) did not significantly perturb the bilayer (see e.g., Figs. 2 *B* and 4 *A*). Thus, although extended simulation times per window would doubtless improve the convergence of the PMF, we remain confident that 10 ns windows (32) provide a reasonable first estimate of the free energy landscape experienced by capsaicin in a bilayer.

### Adding the S1–S4 TM helix domain

**Figure 6:**
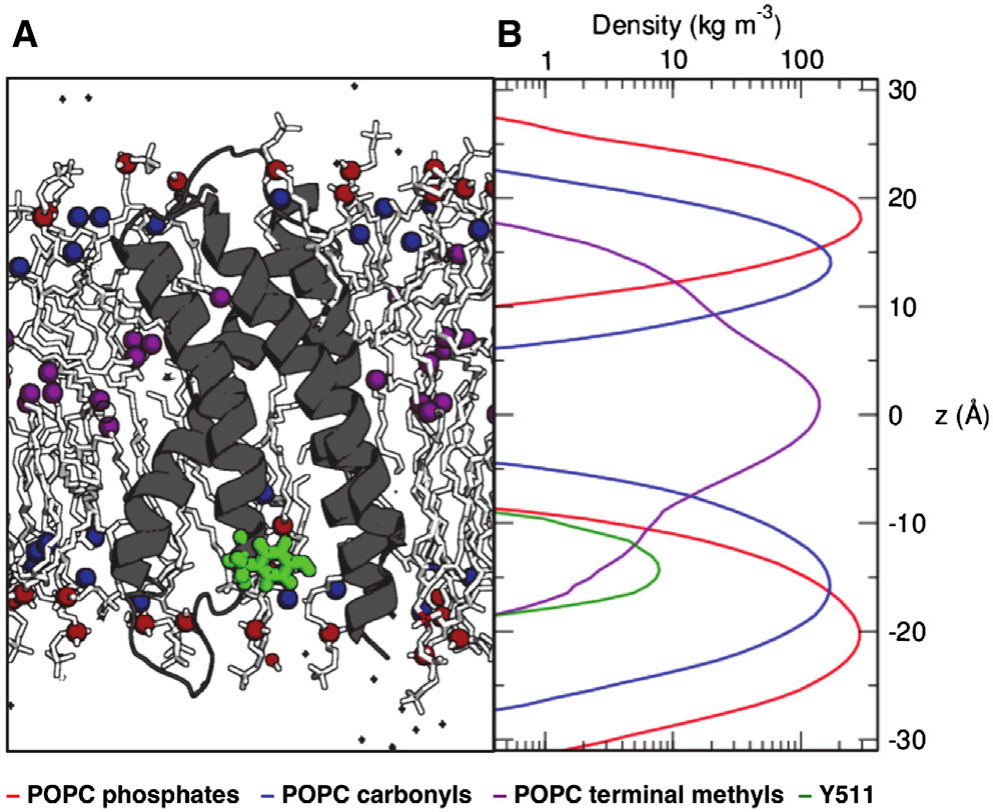
Localization of Y511 of the TRPV1 S1-S4 domain within a POPC bilayer. (*A*) Simulations of the S1–S4 domain from the TRPV1 structure provided an estimate of the location within a lipid bilayer of Y511 (*B*), which is known to play a key role in capsaicin binding. POPC phosphates, carbonyls, and terminal methyls are shown in red, blue, and purple, respectively. The tyrosine is seen to localize alongside the carbonyls of the lipids, similar to the A-group of the capsaicin. To see this figure in color, go online.

As described previously, capsaicin has been suggested to interact with Y511 at the intracellular side of the S3 TM helix. To investigate how the position of this tyrosine relative to the lipid bilayer compared to the localization of capsaicin within the membrane, we performed simulations of the electron microscopy (EM) structure of the S1–S4 helices of TRPV1 in a POPC bilayer. The partial density profile (Fig. 6) reveals that Y511 is located in the interfacial region of the bilayer at *z* ∼ −15 Å, close to the lipid carbonyl groups. This corresponds exactly to the minimum energy location of the capsaicin vanillyl group on the intracellular face of the bilayer (Fig. 3).

We note that despite being isolated from the rest of the full-length TRPV1 channel structure, the S1–S4 helix domain remained relatively stable throughout the simulation (Fig. S4 *A*), as has also been seen in comparable simulations, e.g., of the isolated VSD of Kv channels (60,61). Interestingly, the side chain of Y511 in these simulations was seen to spontaneously flip from the outward-facing orientation, seen in the structure of ligand-free TRPV1, to the inward position seen in the RTX-bound and capsaicin-bound structures (Fig. S4 *B*), even though no ligand was present in the simulations.

**Figure 7:**
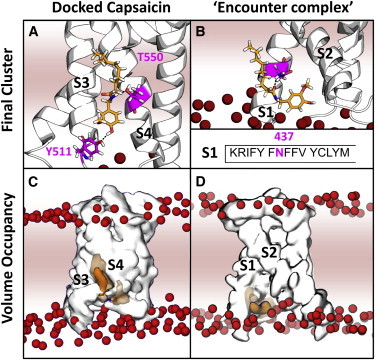
Simulations of TRPV1 S1–S4 with capsaicin. (*A*) The final cluster from analysis of one of the simulations (sim2) starting from a docked capsaicin pose (see Fig. S7 for further details). Stabilizing hydrogen bonding interactions are seen between capsaicin and the side chains of residues Y511 and T550 (*in magenta*). This indicates that capsaicin may remain in a stable bound position with the A group aromatic region facing toward the intracellular phospholipid carbonyls. (*B*) The final cluster from one of the simulations with capsaicin initially in the aqueous phase, revealing that capsaicin interacts with the TRPV1 S1-S4 domain (see Figs. S5 and S6 for further details). The majority of these interactions involved capsaicin initially encountering the S1 helix in the aqueous phase, before ending up fully embedded in the bilayer. An interaction with asparagine 437 (*magenta*) was seen in these simulations. (*C*) Volumetric density map (from VMD, *capsaicin density in orange*, *protein density in white*) over the course of the 50 ns simulation in which capsaicin was initially docked to the TRPV1 S1–S4 (sim2, see *A*). (*D*) The volumetric density map over the course of the 100 ns simulation (see *C*) in which capsaicin was seen to interact with the TPV1 S1-S4 from the aqueous phase. In all four figures POPC phosphates are represented as dark red spheres and the middle of the bilayer as darker pink shading. To see this figure in color, go online.

Further simulations (Fig. 7) of the S1–S4 helices were conducted to include capsaicin either added via the aqueous solution or via insertion into the bilayer. Six 50 or 100 ns duration simulations were conducted for each capsaicin starting position, aqueous phase or lipid. No stable interactions were seen between the capsaicin molecules within the bilayer and the protein. Further simulations (6 × 50 ns) were even conducted using a starting position of all four capsaicin molecules on the Y511 side of the bilayer, with no resulting significant interactions. However, significant capsaicin-TRPV1 interactions occurred in four of the simulations initiated with capsaicin in the aqueous solution (Fig. S5), and all of these interactions occurred at the intracellular side of the S1–S4 helices.

One set of capsaicin-TRPV1 interactions involved an aggregate of two capsaicin molecules that interacted for ∼20 ns with the intracellular S2–S3 loop before diffusing away (Fig. S5 *D*). Other interactions involved capsaicin molecules interacting with the protein while entering the bilayer until they became fully embedded (Fig. S5, *A*–*C*). In three of these instances, the interaction was with the S1 of the protein, rather than the S2–S4 (Fig. 7 *D*). Additionally, in the two 100 ns simulation in which this interaction persisted for 90–100 ns, this interaction was with the same asparagine of the S1 (Fig. 7 *B*). This interaction increasingly stabilized over the duration of the simulation (Fig. S6), and though the interaction was unexpected, the final localization was not far from the tyrosine 511 and the EM density for RTX with a minimum distance from the capsaicin molecule to the Y511 side chain of 11.6 Å, though no specific interactions with Y511 were observed.

To further investigate the capsaicin-TRPV1 interaction within the bilayer, simulations of the S1–S4 of TRPV1 were started from a docked pose of capsaicin, in which it was seen to interact with the S1–S4 helices in line with the current predominant model of capsaicin binding, including hydrogen bonding to Y511 and T550 (Fig. S7 *B*). Here, it was seen that this mode of binding was relatively stable, as 2 of 3 50 ns simulations maintained protein-ligand interactions (Fig. S7 *A*). In these simulations the hydrogen bonding network of the capsaicin-TRPV1 interaction was stabilized further and the location of the capsaicin between the S3 and S4 helices remained throughout the simulations (Fig. 7, *A* and *C*). Additionally, in the third simulation in which the capsaicin dissociates from the bound pose, capsaicin remained in the bilayer in the same manner as in the capsaicin/bilayer simulations described earlier (Fig. S7 *C*). Furthermore, if one overlays capsaicin after the 50 ns simulation from of one of these docked poses with the electron density map from the capsaicin-bound TRPV1 structure, an approximate overlap is seen between the A-group of the capsaicin and the density thought to correspond to capsaicin (Fig. S7 *F*).

## Discussion

Our results show the role of the lipid bilayer in capsaicin localization and of flip-flop when considering its binding to TRPV1. The capsaicin A region localizes alongside the phospholipid carbonyls, as does the Y511 of TRPV1. To access the Y511 side chain, externally applied capsaicin needs to flip from the extracellular leaflet to the intracellular leaflet, thereby crossing a barrier of ∼6 kT as judged via the calculation of a PMF (free energy) profile. We have observed this flip occurs spontaneously, albeit rarely, in molecular detail in an unrestrained simulation.

More generally, our studies may be compared to a number of simulations of passive permeation of drugs across cell membranes (26,29,31–33). Although such investigations shed light on the involvement and significance of the lipid bilayer in the activation of TRPV1 by capsaicin and related molecules, it is important to integrate them with our understanding of the nature of the TRPV1 binding site for capsaicin and related ligands, as revealed in the recent cryo-EM structure. A number of questions remain. For example, it is known that aspects of RTX and capsaicin activation of TRPV1 differ despite the two ligands having overlapping binding sites, but to what extent these differences apply to the orientation, access pathways, and binding pocket of the molecules within TRPV1 remains unclear. In addition, the flip of the Y511 side chain between the apo- and ligand-bound structures of the protein, and our observation that this flip can occur spontaneously in simulations of the S1–S4 domain of the protein in a bilayer, opens the question of the exact role of this motion in ligand binding and/or channel activation.

It is possible that capsaicin could form an initial encounter complex with the TRPV1 molecule before binding more tightly in a manner mediated by the Y511 side chain conformational flip (62). Alternatively, it is possible that the absence of long-lived encounters between capsaicin and TRPV1 in the simulations imply that this ligand, in contrast to RTX, interacts only transiently with the channel. We note, for example, that amantadine has been suggested by solution NMR studies to interact with the exterior of the influenza M2 channel protein before binding to the blocking site within the channel seen in x-ray studies (63,64). This (somewhat speculative) interpretation of capsaicin interactions with TRPV1 would be in agreement with our observation of capsaicin interacting with the S1 TM helix. It would be of interest to investigate whether mutations of residues in the internal end of S1, such as N437, alter either the kinetics of binding or bilayer penetration of capsaicin.

Docking attempts to several regions of the TRPV1 structures, did not yield the encounter complex seen in simulations, perhaps due to the absence of a lipid bilayer during docking. Docking did however, yield a plausible canonical bound state involving the previously predicted S3 and S4 residues. Through simulations starting from this docked pose we were able to see that capsaicin could maintain a stable bound orientation with the A-region near the lipid carbonyls, similar to the simulations of capsaicin alone in the bilayer, though in this case stabilized by hydrogen bonding interactions to residues Y511 and T550. The simulation in which capsaicin dissociates from this docked pose also provides an insight into how capsaicin could access this binding site from the flipped orientation in the intracellular leaflet of the bilayer.

**Figure 8:**
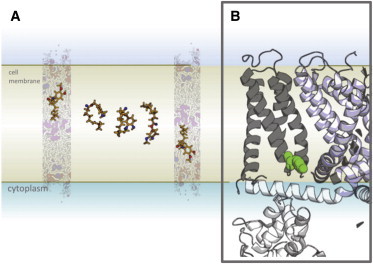
Proposed membrane-mediated interaction of capsaicin binding to TRPV1. Combining images from bilayer localization, capsaicin flip-flop from the extracellular to inner leaflet, the transbilayer PMF profile, and the bilayer localization of tyrosine 511, enables us to propose a possible mechanism of membrane-mediated capsaicin binding: (*A*) capsaicin penetrates the bilayer before flipping from the extracellular to the intracellular leaflet to access (*B*) the transmembrane binding site. To see this figure in color, go online.

By combining interpretations derived from these simulations we are able to present a possible pathway for capsaicin binding to TRPV1 (Fig. 8), whereby capsaicin penetrates the bilayer before flipping from the extracellular to the intracellular leaflet to access the TM-binding site. Although this is not the only possible model, it agrees well both with our observations and with previous literature (12,39). Possible alternative models would suggest either that capsaicin could exit the bilayer on its cytosolic face before interacting with the cytosolic side of the protein to access the binding site or that capsaicin may interact directly with the protein (possibly at the protein lipid interface) to cross the bilayer and reach its final binding site. To understand the barrier presented by the bilayer per se to capsaicin flip-flop from the extracellular leaflet to the cytosolic leaflet (as proposed in our preferred model, see Fig. 8), we have calculated the PMF along the (approximate) bilayer normal. We believe the resulting energetic barrier to inform our discussion of the mode of action of capsaicin, while acknowledging possible limitations of the methodology employed.

A number of recent studies discuss calculations of drug PMF profiles across lipid bilayers, addressing the issues of convergence and of possible local membrane deformations (27,29,59) as well as the difficulties experienced when comparing calculations to experimentally relevant quantities (32) and the importance of increasing timescales and enhancing sampling for such calculations (29). However, our results are in reasonable agreement with previous PMF calculations of amino acids and small molecules across bilayers (33,35,63,65). In particular, the hydrophobic tail of capsaicin is not long enough nor is the molecule sufficiently charged to generate some of the bilayer deformation problems seen in, e.g., studies with antimicrobial peptides (58).

One further possible limitation of our PMF calculations, and of many such calculations in general, is the use of a simple model bilayer (POPC), which fails to address the complexities of composition and asymmetry of cell membranes (66). Such aspects of bilayer composition have proven to be important, for example, in the regulation of TRPV1 by PIP_2_ (67). A further likely limitation concerns our protein simulations, in particular the modest timescales sampled and the isolation of the S1–S4 domain from the full-length TRPV1 protein. Experiments have shown that residues in the N- and C-termini, and even the pore domain are required for capsaicin and RTX activation of TRPV1 (18,19). Thus, further simulations of capsaicin interactions with the full-length protein are desirable in the future. It is likely that calculation of a TRPV1/capsaicin PMF in a lipid bilayer would present issues of convergence and so might require the use of enhanced sampling techniques such as, e.g., metadynamics (68).

Generally, the interaction of lipophilic drugs such as capsaicin with membrane-embedded binding sites requires the consideration of additional parameters to those considered in drug binding events in the aqueous phase. In particular, diffusion of the drug in the spatially inhomogeneous environment presented by cell membranes is likely to be of some importance in determining the kinetics of drug/receptor interactions in a membrane. Although the structures of TRPV1 have illuminated that the vanilloid site is in fact intracellular (10,17) and not almost extracellular as was posited in studies discussing RTX binding to TRPV1 (16,26), questions remain. For example, the involvement of the cytosolic domains, the source of the differences between RTX and capsaicin binding, the mechanism by which ligand binding induces channel opening, and the pathway these hydrophobic molecules take before binding the membrane-embedded binding site all require further investigation. The current study has helped to inform at least the last of these aspects of understanding how capsaicin binds to TRPV1, thus contributing to an improved understanding of how to better target TRPV1 with capsaicin-like compounds.

## Figures and Tables

**Table 1:** Summary of simulations

Number of capsaicin and initial location	Replicas and duration (ns)	Protein
4× aqueous	2 × 100 and 1 × 500	–
4× lipid	1 × 500	–
none (control)	1 × 500	–
1 Å spaced windows along *z* for PMF	76 × 10	–
4× aqueous	4 × 50 and 2 × 100	rV1 S1–S4 (3J5P)
4× lipid	4 × 50 and 2 × 100	rV1 S1–S4 (3J5P)
4× lipid (all at intracellular leaflet)	6 × 50	rV1 S1–S4 (3J5P)
1× docked to Y511 site	3 × 50	rV1 S1–S4 (3J5R)
none (control)	3 × 100	rV1 S1–S4 (3J5P)

## Supporting Material

Document S1. Seven figures and the capsaicin parameters

Document S2. Article plus Supporting Material
