# Supplementary material for: Capsaicin Interaction with TRPV1 Channels in a Lipid Bilayer: Molecular Dynamics Simulation
Source: Biophys J. 2015 Mar 24;108(6):1425–34. doi: 10.1016/j.bpj.2015.02.013 (PMC4375533; doi:10.1016/j.bpj.2015.02.013)
Supplement: Document S1. Seven figures and the capsaicin parameters [file mmc1.pdf]

Supporting Material for:

Capsaicin Interaction with TRPV1 Channels in a Lipid Bilayer: Molecular Dynamics Simulation

Sonya M. Hanson, Simon Newstead, Kenton J. Swartz, & Mark S.P. Sansom

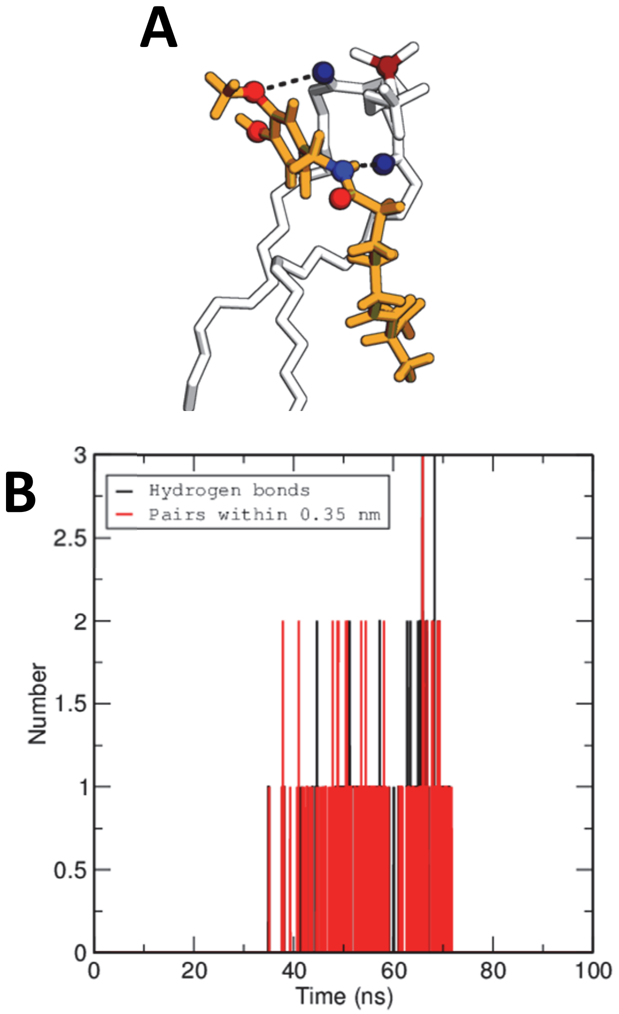

FIGURE S1 **POPC and Capsaicin hydrogen bonding interaction.** *(A)* An example of a long-lived hydrogen bonding interaction between a carbonyl group of a POPC molecule (in white) and the B region of capsaicin (in orange). *(B)* Number of H-bonds vs. time between capsaicin and the selected POPC molecule. This interaction is maintained for more than 30 ns of the simulation.

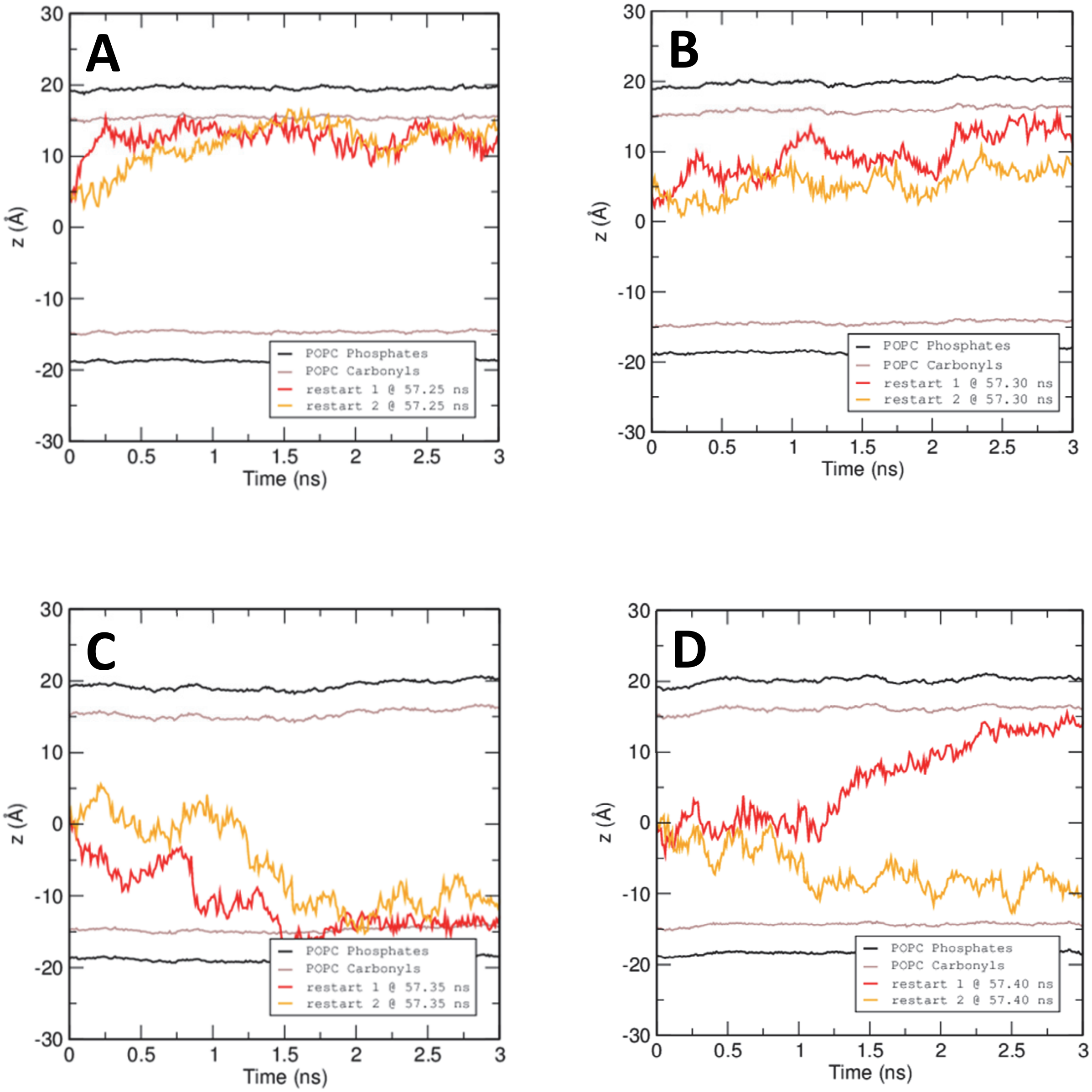

FIGURE S2 **Multiple simulations initiated at selected points along the original capsaicin flip-flop trajectory.** *(A)* Shows two repeats (red and orange lines) with different random velocity seeds using a starting position at 57.25 ns, *(B)* at 57.30 ns, *(C)* at 57.35 ns, and *(D)* at 57.40 ns along the original trajectory which yielded a flip-flop event. Red or orange lines represent the center of mass of capsaicin during different repeats. Black lines represent the phosphate head groups of POPC. Brown lines represent the carbonyls of POPC.

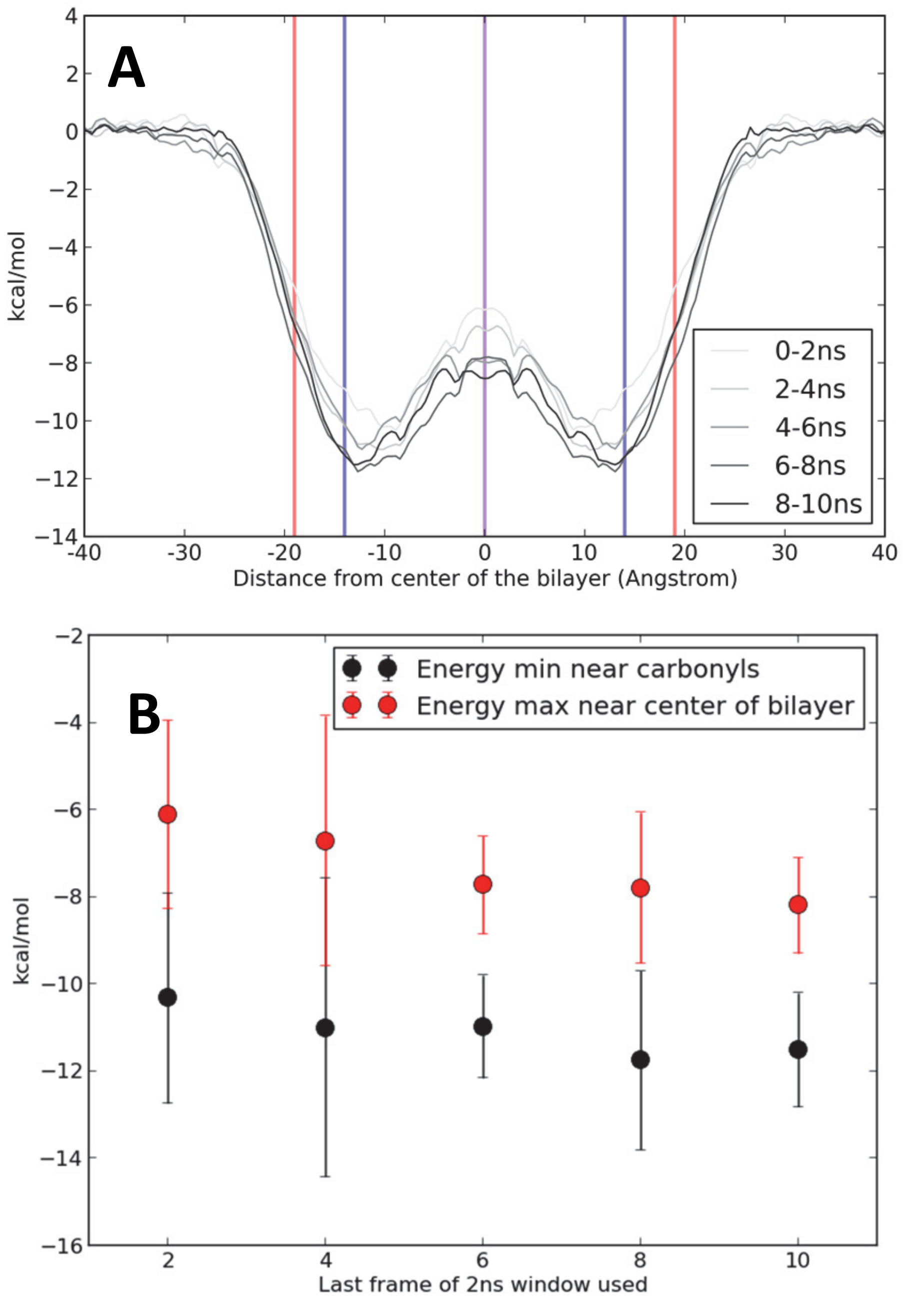

FIGURE S3 **PMF Convergence.** *(A)* PMF profiles as calculated from consecutive 2 ns segments of the full 10 ns simulations of 1 Å windows across the bilayer. *(B)* The values of the energy minima near the carbonyls (z = ±12 Å) and the energy maxima near the center of the bilayer (z = 0 Å) were calculated for the consecutive PMFs in *A* to provide a measure of convergence of the free energy profile.

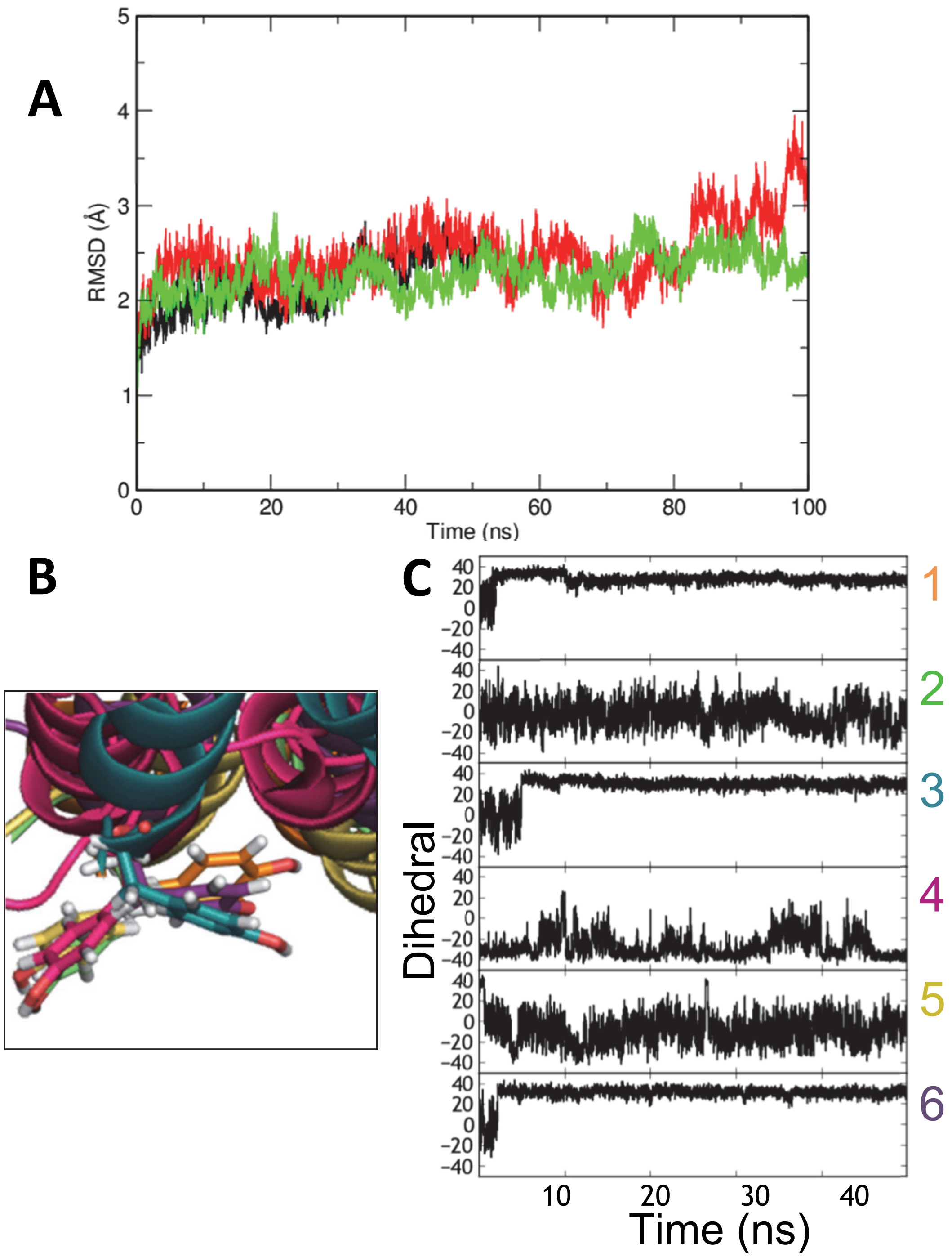

FIGURE S4 **Analysis of stability of the TRPV1 S1-S4 in model membrane.** *(A)* The backbone RMSD from the initial structure of the S1-S4 domain of TRPV1 over the course of three repeat 100 ns simulations. *(B)* The flip from an outward to an inward conformation of the sidechain of tyrosine 511. *(C)* Changes in the X_1 dihedral angle of Tyr511 over time of six separate simulations (colored accordingly) of the S1-S4 of the apo EM structure in a POPC bilayer.

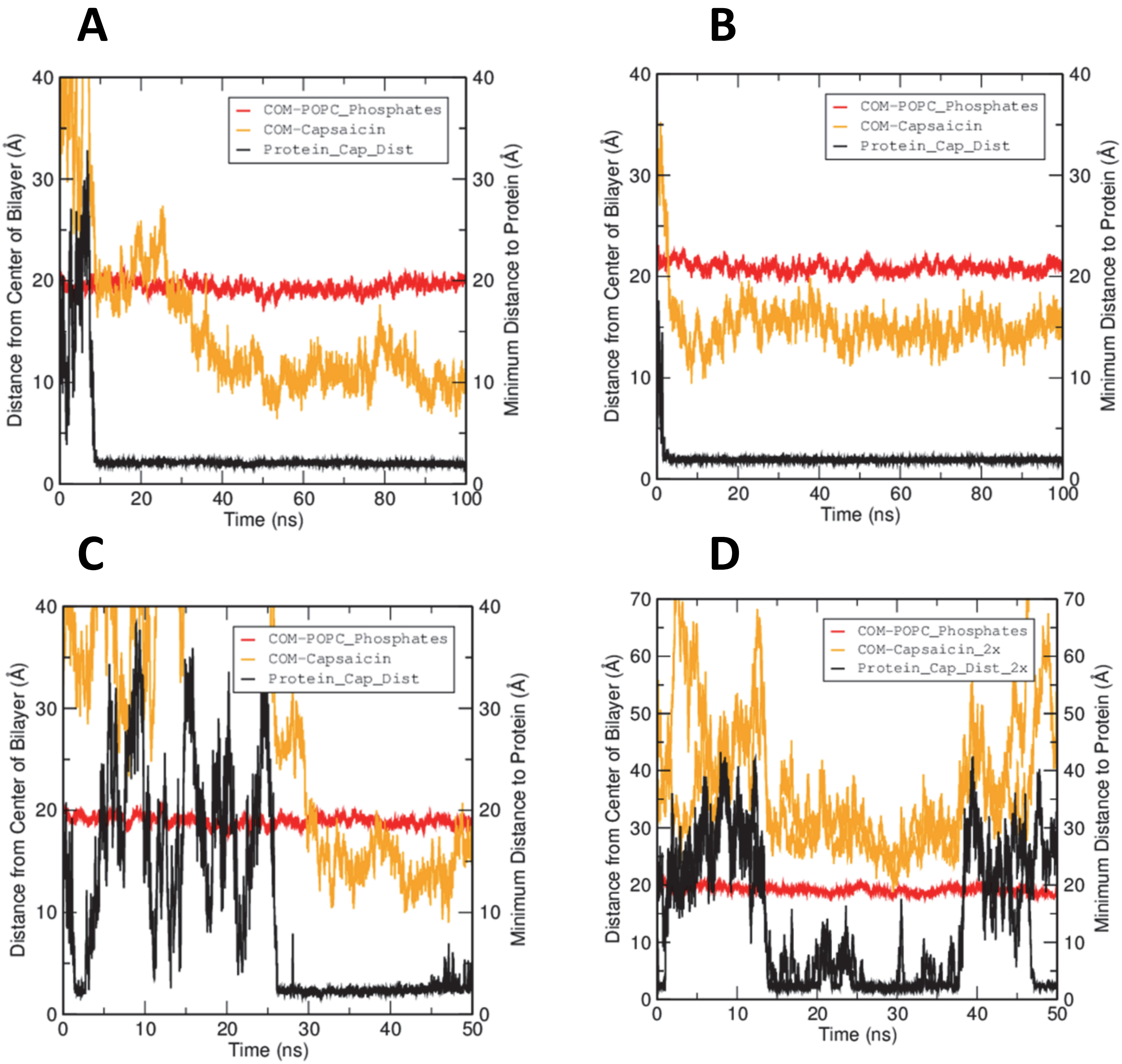

FIGURE S5 **Capsaicin interaction with TRPV1 S1-S4 compared to its membrane penetration.** Plots of the minimum distance of interacting capsaicin molecules to the TRPV1 S1-S4 domain over the course of four simulations (black), overlayed with the bilayer penetration as shown by the center of mass of capsaicin (orange) relative to that of the phosphates of the intracellular leaflet (red). This is shown for two capsaicin molecules that interact with the S1 helix *(A)* and *(B)*, and for three capsaicin molecules that interact with the S2-S3 loop *(C)* and *(D)*. *(D)* is an interaction between a two-capsaicin aggregate and the TRPV1 S1-S4. Notice how in A-C the capsaicin interacts with the protein before and during bilayer penetration.

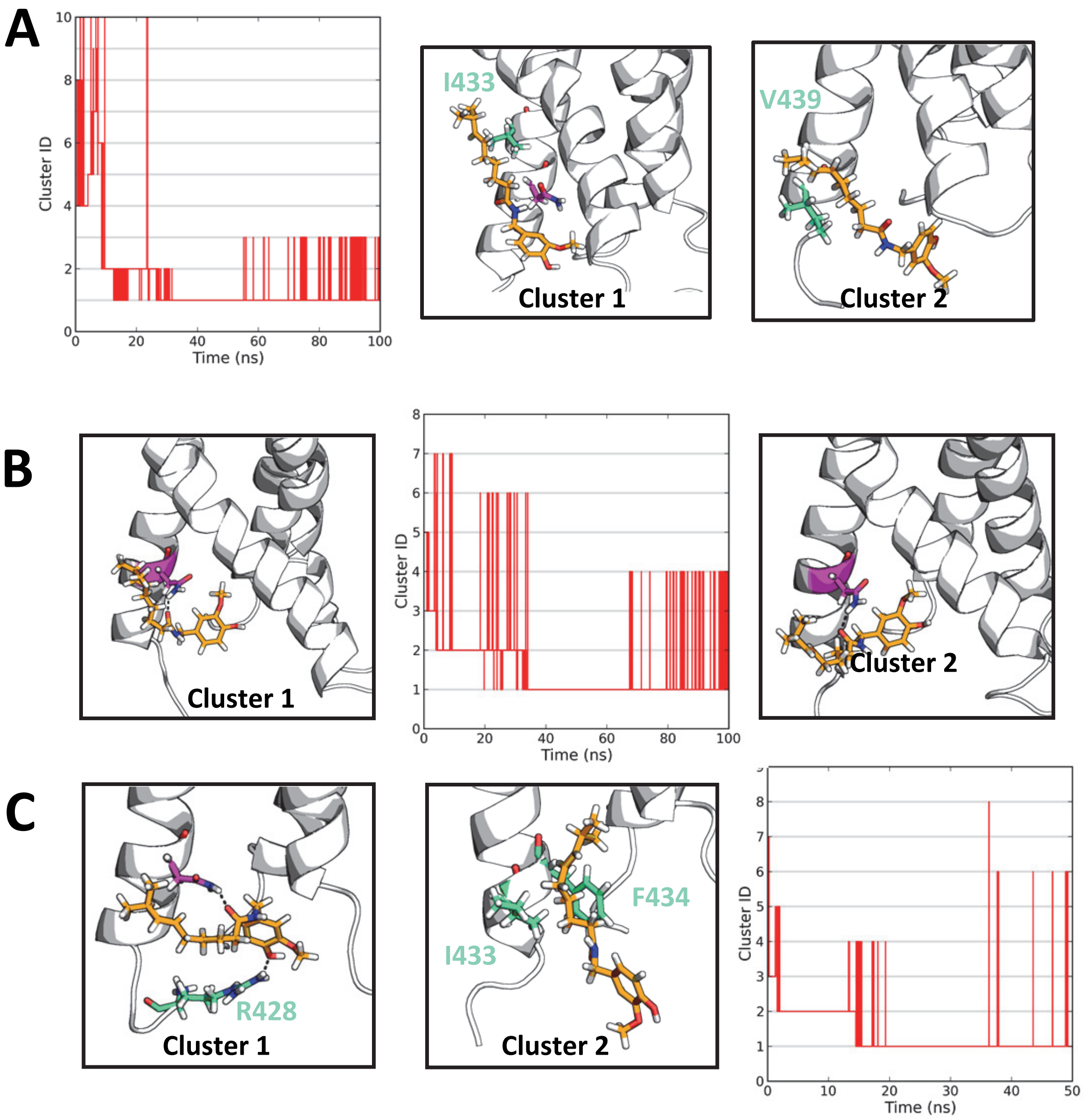

FIGURE S6 **Results of cluster analysis for 'encounter complex' simulations in which interactions are seen between capsaicin and the S1 helix.** *(A)* Cluster analysis of one 100 ns simulation results in a final cluster that involves N437 (magenta), but also I433 (teal). In the second-most dominant cluster the interaction with N437 is not seen, and V439 (teal) instead is seen to interact with capsaicin, perhaps on it's way to a more stable pose. *(B)* This 100 ns simulation is also the one shown in Fig. *7*. Cluster analysis shows a dominant cluster in which capsaicin interacts with N437 (magenta). The second-most dominant cluster also shares this interaction, but the C group acyl chain tail is in a different orientation. *(C)* In this 50 ns simulation, an interaction between capsaicin and the S1 helix is also seen, and the N437 (magenta) interaction is once again observed in the most dominant cluster. However, the R428 (teal) interaction is also seen. This interaction may be an artifact of the S1-S4 helices being truncated from the rest of the full length protein, although it is not seen in the other two simulations.

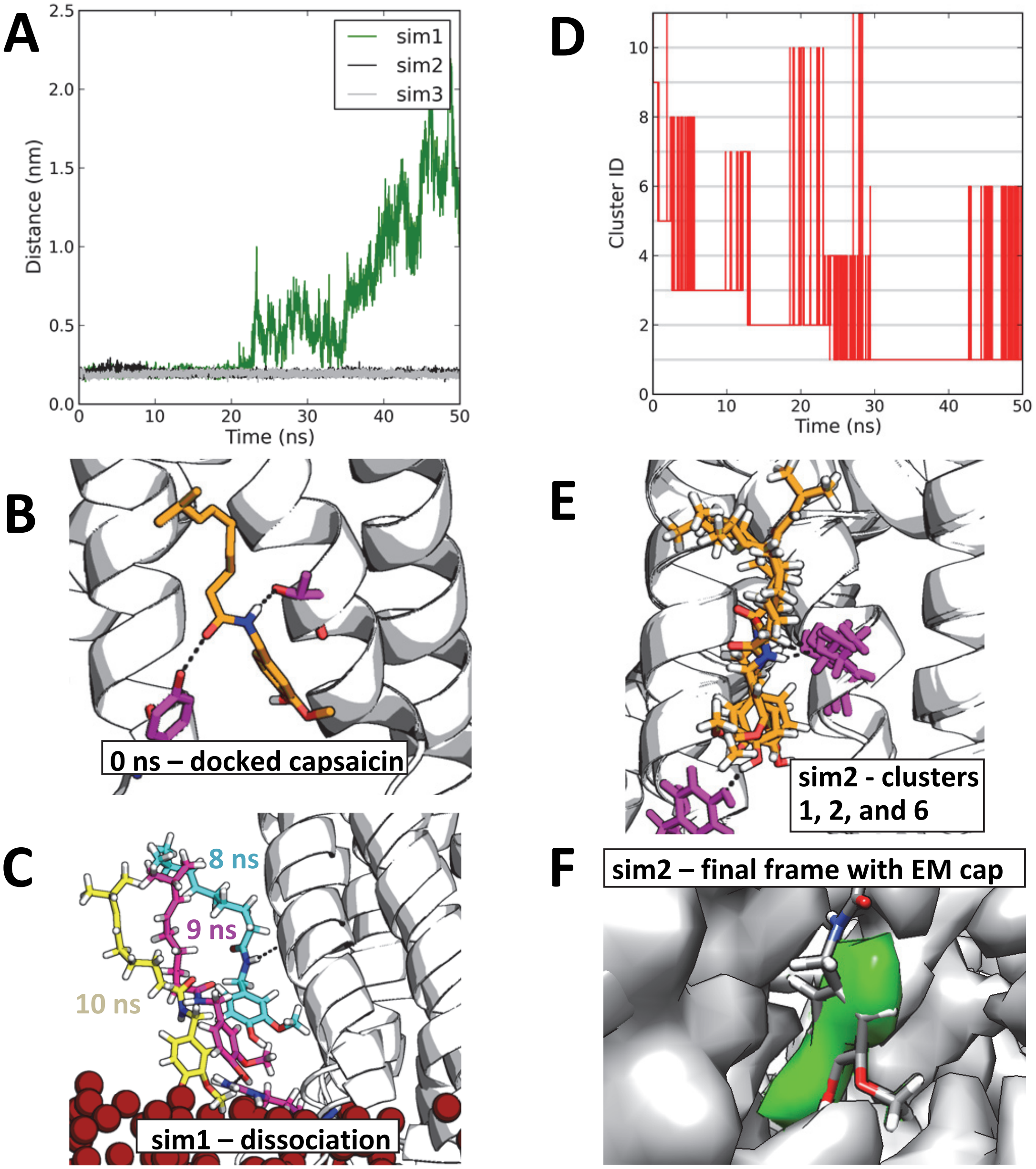

FIGURE S7 **Results of 50ns simulation of capsaicin docked to TRPV1.** *(A)* In two out of three simulations capsaicin continued to equilibrate within the docked position between the S3 and S4. *(B)* The docked pose of capsaicin was the top-ranked pose found using the Autodock Vina program and the full-length TRPV1 (PDB ID: 3J5R). T550 and Y511 are shown in magenta. *(C)* In the simulation in which capsaicin dissociated from the S1-S4 helices it initially had formed a hydrogen bond with T550 (cyan) and then began to dissociate, interacting with an arginine at the intracellular side of the S4 (magenta) and then finally with only the POPC molecules (yellow) in a position seen in the capsaicin-bilayer only simulations. *(D)* Cluster analysis of one of the simulations in which capsaicin remained in the docked pose shows small shifts toward a final cluster. *(E)* Three of the dominant clusters from D (1, 2, and 6) are shown. A change from the initial docked pose is that now Y511 forms a hydrogen bonding interaction with the A group aromatic region instead of the B group. *(F)* An overlay of the final frame of this simulation shows that the A group of capsaicin overlaps with density suspected to correspond to capsaicin in the sharpened EM density (deep green) of the capsaicin bound structure (temperature factor -200 Å, filtered to 4.2 Å, shown at sigma level 8).

Supporting Material - Parameter (*.itp) file for capsaicin

```
 [ moleculetype ]
; name  nrexcl
CAP   3

 [ atoms ]
;  nr    type   resnr   residu   atom   cgnr    charge      mass
    1    opls_145     1        CAP      C1    1  -0.11500  12.01100       ;  -0.1150000
    2    opls_146     1        CAP     H12    1   0.11500   1.00800       ;   0.0000000
    3    opls_145     1        CAP      C2    2  -0.11500  12.01100       ;  -0.1150000
    4    opls_145     1        CAP      C3    3  -0.11500  12.01100       ;  -0.2300000
    5    opls_146     1        CAP     H13    3   0.11500   1.00800       ;  -0.1150000
    6    opls_145     1        CAP      C4    4  -0.11500  12.01100       ;  -0.2300000
    7    opls_146     1        CAP     H14    4   0.11500   1.00800       ;  -0.1150000
    8    opls_166     1        CAP      C5    5   0.15000  12.01100       ;   0.0350000
    9    opls_166     1        CAP      C6    6   0.15000  12.01100       ;   0.1850000
   10    opls_179     1        CAP      O1    6  -0.28500  15.99940       ;  -0.1000000
   11    opls_157     1        CAP      C7    7   0.13000  12.01100       ;   0.0450000
   12    opls_156     1        CAP     H17    7   0.04000   1.00800       ;   0.0850000
   13    opls_156     1        CAP     H18    7   0.04000   1.00800       ;   0.1250000
   14    opls_156     1        CAP     H19    7   0.04000   1.00800       ;   0.1650000
   15    opls_167     1        CAP      O2    5  -0.58500  15.99940       ;  -0.4200000
   16    opls_168     1        CAP     H20    5   0.43500   1.00800       ;   0.0150000
   17    opls_244     1        CAP      C8    8   0.08000  12.01100       ;   0.0950000
   18    opls_140     1        CAP     H10    8   0.06000   1.00800       ;   0.1550000
   19    opls_140     1        CAP     H11    8   0.06000   1.00800       ;   0.2150000
   20    opls_238     1        CAP      N1    8  -0.50000  14.00670       ;  -0.2850000
   21    opls_241     1        CAP     H21    8   0.30000   1.00800       ;   0.0150000
   22    opls_235     1        CAP      C9    9   0.50000  12.01100       ;   0.5150000
   23    opls_136     1        CAP     C10   10  -0.12000  12.01100       ;   0.3950000
   24    opls_140     1        CAP      H8   10   0.06000   1.00800       ;   0.4550000
   25    opls_140     1        CAP      H9   10   0.06000   1.00800       ;   0.5150000
   26    opls_236     1        CAP      O3    9  -0.50000  15.99940       ;   0.0150000
   27    opls_136     1        CAP     C11   11  -0.12000  12.01100       ;  -0.1050000
   28    opls_140     1        CAP      H7   11   0.06000   1.00800       ;  -0.0450000
   29    opls_140     1        CAP     H16   11   0.06000   1.00800       ;   0.0150000
   30    opls_136     1        CAP     C12   12  -0.12000  12.01100       ;  -0.1050000
   31    opls_140     1        CAP      H5   12   0.06000   1.00800       ;  -0.0450000
   32    opls_140     1        CAP      H6   12   0.06000   1.00800       ;   0.0150000
   33    opls_136     1        CAP     C13   13  -0.12000  12.01100       ;  -0.1050000
   34    opls_140     1        CAP      H4   13   0.06000   1.00800       ;  -0.0450000
   35    opls_140     1        CAP     H15   13   0.06000   1.00800       ;   0.0150000
   36    opls_142     1        CAP     C14   14  -0.11500  12.01100       ;  -0.1000000
   37    opls_144     1        CAP      H3   14   0.11500   1.00800       ;   0.0150000
   38    opls_142     1        CAP     C15   15  -0.11500  12.01100       ;  -0.1000000
   39    opls_144     1        CAP      H1   15   0.11500   1.00800       ;   0.0150000
   40    opls_137     1        CAP     C16   16  -0.06000  12.01100       ;  -0.0450000
   41    opls_140     1        CAP      H2   16   0.06000   1.00800       ;   0.0150000
   42    opls_135     1        CAP     C17   17  -0.18000  12.01100       ;  -0.1650000
   43    opls_140     1        CAP     H25   17   0.06000   1.00800       ;  -0.1050000
   44    opls_140     1        CAP     H26   17   0.06000   1.00800       ;  -0.0450000
   45    opls_140     1        CAP     H27   17   0.06000   1.00800       ;   0.0150000
   46    opls_135     1        CAP     C18   18  -0.18000  12.01100       ;  -0.1650000
   47    opls_140     1        CAP     H22   18   0.06000   1.00800       ;  -0.1050000
   48    opls_140     1        CAP     H23   18   0.06000   1.00800       ;  -0.0450000
   49    opls_140     1        CAP     H24   18   0.06000   1.00800       ;   0.0150000
; total molecule charge =   0.0000000


 [ bonds ]
;   ai  aj   funct      b0          kb
       1     3   1     0.14000      392459.       ;    C1-    C2
       1     9   1     0.14000      392459.       ;    C1-    C6
       3     4   1     0.14000      392459.       ;    C2-    C3
```

```
     4     6   1     0.14000      392459.       ;    C3-    C4
     6     8   1     0.14000      392459.       ;    C4-    C5
     8     9   1     0.14000      392459.       ;    C5-    C6
     9    10   1     0.13640      376560.       ;    C6-    O1
    10    11   1     0.14100      267776.       ;    O1-    C7
     8    15   1     0.13640      376560.       ;    C5-    O2
     3    17   1     0.15100      265266.       ;    C2-    C8
    17    20   1     0.14490      282002.       ;    C8-    N1
    20    22   1     0.13350      410032.       ;    N1-    C9
    22    23   1     0.15220      265266.       ;    C9-   C10
    22    26   1     0.12290      476976.       ;    C9-    O3
    23    27   1     0.15290      224262.       ;   C10-   C11
    27    30   1     0.15290      224262.       ;   C11-   C12
    30    33   1     0.15290      224262.       ;   C12-   C13
    33    36   1     0.15100      265266.       ;   C13-   C14
    36    38   1     0.13400      459403.       ;   C14-   C15
    38    40   1     0.15100      265266.       ;   C15-   C16
    40    42   1     0.15290      224262.       ;   C16-   C17
    40    46   1     0.15290      224262.       ;   C16-   C18
    38    39   1     0.10800      307106.       ;   C15-    H1
    40    41   1     0.10900      284512.       ;   C16-    H2
    36    37   1     0.10800      307106.       ;   C14-    H3
    33    34   1     0.10900      284512.       ;   C13-    H4
    30    31   1     0.10900      284512.       ;   C12-    H5
    30    32   1     0.10900      284512.       ;   C12-    H6
    27    28   1     0.10900      284512.       ;   C11-    H7
    23    24   1     0.10900      284512.       ;   C10-    H8
    23    25   1     0.10900      284512.       ;   C10-    H9
    17    18   1     0.10900      284512.       ;    C8-   H10
    17    19   1     0.10900      284512.       ;    C8-   H11
     1     2   1     0.10800      307106.       ;    C1-   H12
     4     5   1     0.10800      307106.       ;    C3-   H13
     6     7   1     0.10800      307106.       ;    C4-   H14
    33    35   1     0.10900      284512.       ;   C13-   H15
    27    29   1     0.10900      284512.       ;   C11-   H16
    11    12   1     0.10900      284512.       ;    C7-   H17
    11    13   1     0.10900      284512.       ;    C7-   H18
    11    14   1     0.10900      284512.       ;    C7-   H19
    15    16   1     0.09450      462750.       ;    O2-   H20
    20    21   1     0.10100      363171.       ;    N1-   H21
    46    47   1     0.10900      284512.       ;   C18-   H22
    46    48   1     0.10900      284512.       ;   C18-   H23
    46    49   1     0.10900      284512.       ;   C18-   H24
    42    43   1     0.10900      284512.       ;   C17-   H25
    42    44   1     0.10900      284512.       ;   C17-   H26
    42    45   1     0.10900      284512.       ;   C17-   H27


[ constraints ]
     8      16   2     0.19393       ;    C5     H20

[ pairs ]
     9       4  1       ;    C6-    C3
     9      17  1       ;    C6-    C8
     2       4  1       ;   H12-    C3
     2      17  1       ;   H12-    C8
     3       8  1       ;    C2-    C5
     3      10  1       ;    C2-    O1
     2       8  1       ;   H12-    C5
     2      10  1       ;   H12-    O1
     1       6  1       ;    C1-    C4
     1       5  1       ;    C1-   H13
    17       6  1       ;    C8-    C4
    17       5  1       ;    C8-   H13
     3       7  1       ;    C2-   H14
```

5	8	1	;	H13-	C5
5	7	1	;	H13-	H14
4	15	1	;	C3-	O2
7	9	1	;	H14-	C6
7	15	1	;	H14-	O2
6	10	1	;	C4-	O1
15	1	1	;	O2-	C1
15	10	1	;	O2-	O1
1	11	1	;	C1-	C7
8	11	1	;	C5-	C7
9	12	1	;	C6-	H17
9	13	1	;	C6-	H18
9	14	1	;	C6-	H19
6	16	1	;	C4-	H20
9	16	1	;	C6-	H20
1	20	1	;	C1-	N1
1	18	1	;	C1-	H10
1	19	1	;	C1-	H11
4	20	1	;	C3-	N1
4	18	1	;	C3-	H10
4	19	1	;	C3-	H11
3	22	1	;	C2-	C9
3	21	1	;	C2-	H21
18	22	1	;	H10-	C9
18	21	1	;	H10-	H21
19	22	1	;	H11-	C9
19	21	1	;	H11-	H21
17	23	1	;	C8-	C10
17	26	1	;	C8-	O3
21	23	1	;	H21-	C10
21	26	1	;	H21-	O3
20	27	1	;	N1-	C11
20	24	1	;	N1-	H8
20	25	1	;	N1-	H9
26	27	1	;	O3-	C11
26	24	1	;	O3-	H8
26	25	1	;	O3-	H9
22	30	1	;	C9-	C12
22	28	1	;	C9-	H7
22	29	1	;	C9-	H16
24	30	1	;	H8-	C12
24	28	1	;	H8-	H7
24	29	1	;	H8-	H16
25	30	1	;	H9-	C12
25	28	1	;	H9-	H7
25	29	1	;	H9-	H16
23	33	1	;	C10-	C13
23	31	1	;	C10-	H5
23	32	1	;	C10-	H6
28	33	1	;	H7-	C13
28	31	1	;	H7-	H5
28	32	1	;	H7-	H6
29	33	1	;	H16-	C13
29	31	1	;	H16-	H5
29	32	1	;	H16-	H6
27	36	1	;	C11-	C14
27	34	1	;	C11-	H4
27	35	1	;	C11-	H15
31	36	1	;	H5-	C14
31	34	1	;	H5-	H4
31	35	1	;	H5-	H15
32	36	1	;	H6-	C14
32	34	1	;	H6-	H4
32	35	1	;	H6-	H15
30	38	1	;	C12-	C15

```
     30      37  1       ;   C12-    H3
     34      38  1       ;    H4-   C15
     34      37  1       ;    H4-    H3
     35      38  1       ;   H15-   C15
     35      37  1       ;   H15-    H3
     33      40  1       ;   C13-   C16
     33      39  1       ;   C13-    H1
     37      40  1       ;    H3-   C16
     37      39  1       ;    H3-    H1
     36      42  1       ;   C14-   C17
     36      46  1       ;   C14-   C18
     36      41  1       ;   C14-    H2
     39      42  1       ;    H1-   C17
     39      46  1       ;    H1-   C18
     39      41  1       ;    H1-    H2
     38      43  1       ;   C15-   H25
     38      44  1       ;   C15-   H26
     38      45  1       ;   C15-   H27
     46      43  1       ;   C18-   H25
     46      44  1       ;   C18-   H26
     46      45  1       ;   C18-   H27
     41      43  1       ;    H2-   H25
     41      44  1       ;    H2-   H26
     41      45  1       ;    H2-   H27
     38      47  1       ;   C15-   H22
     38      48  1       ;   C15-   H23
     38      49  1       ;   C15-   H24
     42      47  1       ;   C17-   H22
     42      48  1       ;   C17-   H23
     42      49  1       ;   C17-   H24
     41      47  1       ;    H2-   H22
     41      48  1       ;    H2-   H23
     41      49  1       ;    H2-   H24

[ angles ]
; ai  aj  ak  funct      th0         cth
     9     1     3  1     120.000    527.1840     ;    C6-    C1-    C2
     2     1     3  1     120.000    292.8800     ;   H12-    C1-    C2
     1     3     4  1     120.000    527.1840     ;    C1-    C2-    C3
     1     3    17  1     120.000    585.7600     ;    C1-    C2-    C8
     2     1     9  1     120.000    292.8800     ;   H12-    C1-    C6
     1     9     8  1     120.000    527.1840     ;    C1-    C6-    C5
     1     9    10  1     120.000    585.7600     ;    C1-    C6-    O1
    17     3     4  1     120.000    585.7600     ;    C8-    C2-    C3
     3     4     6  1     120.000    527.1840     ;    C2-    C3-    C4
     3     4     5  1     120.000    292.8800     ;    C2-    C3-   H13
     5     4     6  1     120.000    292.8800     ;   H13-    C3-    C4
     4     6     8  1     120.000    527.1840     ;    C3-    C4-    C5
     4     6     7  1     120.000    292.8800     ;    C3-    C4-   H14
     7     6     8  1     120.000    292.8800     ;   H14-    C4-    C5
     6     8     9  1     120.000    527.1840     ;    C4-    C5-    C6
     6     8    15  1     120.000    585.7600     ;    C4-    C5-    O2
    15     8     9  1     120.000    585.7600     ;    O2-    C5-    C6
     8     9    10  1     120.000    585.7600     ;    C5-    C6-    O1
     9    10    11  1     111.000    627.6000     ;    C6-    O1-    C7
    10    11    12  1     109.500    292.8800     ;    O1-    C7-   H17
    10    11    13  1     109.500    292.8800     ;    O1-    C7-   H18
    10    11    14  1     109.500    292.8800     ;    O1-    C7-   H19
     8    15    16  1     113.000    292.8800     ;    C5-    O2-   H20
     3    17    20  1     109.700    669.4400     ;    C2-    C8-    N1
     3    17    18  1     109.500    292.8800     ;    C2-    C8-   H10
     3    17    19  1     109.500    292.8800     ;    C2-    C8-   H11
    18    17    20  1     109.500    292.8800     ;   H10-    C8-    N1
    19    17    20  1     109.500    292.8800     ;   H11-    C8-    N1
    17    20    22  1     121.900    418.4000     ;    C8-    N1-    C9
```

17	20	21	1	118.400	317.9840	;	C8-	N1-	H21
21	20	22	1	119.800	292.8800	;	H21-	N1-	C9
20	22	23	1	116.600	585.7600	;	N1-	C9-	C10
20	22	26	1	122.900	669.4400	;	N1-	C9-	O3
26	22	23	1	120.400	669.4400	;	O3-	C9-	C10
22	23	27	1	111.100	527.1840	;	C9-	C10-	C11
22	23	24	1	109.500	292.8800	;	C9-	C10-	H8
22	23	25	1	109.500	292.8800	;	C9-	C10-	H9
24	23	27	1	110.700	313.8000	;	H8-	C10-	C11
25	23	27	1	110.700	313.8000	;	H9-	C10-	C11
23	27	30	1	112.700	488.2730	;	C10-	C11-	C12
23	27	28	1	110.700	313.8000	;	C10-	C11-	H7
23	27	29	1	110.700	313.8000	;	C10-	C11-	H16
28	27	30	1	110.700	313.8000	;	H7-	C11-	C12
29	27	30	1	110.700	313.8000	;	H16-	C11-	C12
27	30	33	1	112.700	488.2730	;	C11-	C12-	C13
27	30	31	1	110.700	313.8000	;	C11-	C12-	H5
27	30	32	1	110.700	313.8000	;	C11-	C12-	H6
31	30	33	1	110.700	313.8000	;	H5-	C12-	C13
32	30	33	1	110.700	313.8000	;	H6-	C12-	C13
30	33	36	1	111.100	527.1840	;	C12-	C13-	C14
30	33	34	1	110.700	313.8000	;	C12-	C13-	H4
30	33	35	1	110.700	313.8000	;	C12-	C13-	H15
34	33	36	1	109.500	292.8800	;	H4-	C13-	C14
35	33	36	1	109.500	292.8800	;	H15-	C13-	C14
33	36	38	1	124.000	585.7600	;	C13-	C14-	C15
33	36	37	1	117.000	292.8800	;	filled		
37	36	38	1	120.000	292.8800	;	H3-	C14-	C15
36	38	40	1	124.000	585.7600	;	C14-	C15-	C16
36	38	39	1	120.000	292.8800	;	C14-	C15-	H1
39	38	40	1	117.000	292.8800	;	filled		
38	40	42	1	111.100	527.1840	;	C15-	C16-	C17
38	40	46	1	111.100	527.1840	;	C15-	C16-	C18
38	40	41	1	109.500	292.8800	;	C15-	C16-	H2
46	40	42	1	112.700	488.2730	;	C18-	C16-	C17
41	40	42	1	110.700	313.8000	;	H2-	C16-	C17
40	42	43	1	110.700	313.8000	;	C16-	C17-	H25
40	42	44	1	110.700	313.8000	;	C16-	C17-	H26
40	42	45	1	110.700	313.8000	;	C16-	C17-	H27
41	40	46	1	110.700	313.8000	;	H2-	C16-	C18
40	46	47	1	110.700	313.8000	;	C16-	C18-	H22
40	46	48	1	110.700	313.8000	;	C16-	C18-	H23
40	46	49	1	110.700	313.8000	;	C16-	C18-	H24
35	33	34	1	107.800	276.1440	;	H15-	C13-	H4
32	30	31	1	107.800	276.1440	;	H6-	C12-	H5
29	27	28	1	107.800	276.1440	;	H16-	C11-	H7
25	23	24	1	107.800	276.1440	;	H9-	C10-	H8
19	17	18	1	107.800	276.1440	;	H11-	C8-	H10
13	11	12	1	107.800	276.1440	;	H18-	C7-	H17
14	11	12	1	107.800	276.1440	;	H19-	C7-	H17
14	11	13	1	107.800	276.1440	;	H19-	C7-	H18
48	46	47	1	107.800	276.1440	;	H23-	C18-	H22
49	46	47	1	107.800	276.1440	;	H24-	C18-	H22
49	46	48	1	107.800	276.1440	;	H24-	C18-	H23
44	42	43	1	107.800	276.1440	;	H26-	C17-	H25
45	42	43	1	107.800	276.1440	;	H27-	C17-	H25
45	42	44	1	107.800	276.1440	;	H27-	C17-	H26

```
[ dihedrals ]
;  ai    aj    ak    al funct           c0           c1           c2           c3           c4           c5
     9     1     3     4   3  30.33400   0.00000 -30.33400   0.00000   0.00000   0.00000 ; dih    C6-    C1-    C2-    C3
     3     1     9     8   3  30.33400   0.00000 -30.33400   0.00000   0.00000   0.00000 ; dih    C2-    C1-    C6-    C5
     1     3     4     6   3  30.33400   0.00000 -30.33400   0.00000   0.00000   0.00000 ; dih    C1-    C2-    C3-    C4
     3     4     6     8   3  30.33400   0.00000 -30.33400   0.00000   0.00000   0.00000 ; dih    C2-    C3-    C4-    C5
     4     6     8     9   3  30.33400   0.00000 -30.33400   0.00000   0.00000   0.00000 ; dih    C3-    C4-    C5-    C6
     6     8     9     1   3  30.33400   0.00000 -30.33400   0.00000   0.00000   0.00000 ; dih    C4-    C5-    C6-    C1
     1     9    10    11   3  12.55200   0.00000 -12.55200   0.00000   0.00000   0.00000 ; dih    C1-    C6-    O1-    C7
     9    10    11    12   3   1.58992   4.76976   0.00000  -6.35968   0.00000   0.00000 ; dih    C6-    O1-    C7-   H17
     6     8    15    16   3   7.03749   0.00000  -7.03749   0.00000   0.00000   0.00000 ; dih    C4-    C5-    O2-   H20
     1     3    17    20   3   0.00000   0.00000   0.00000   0.00000   0.00000   0.00000 ; dih    C1-    C2-    C8-    N1
     3    17    20    22   3  -4.70700   2.92044   1.78656   0.00000   0.00000   0.00000 ; dih    C2-    C8-    N1-    C9
    17    20    22    23   3  30.28798  -4.81160 -25.47638   0.00000   0.00000   0.00000 ; dih    C8-    N1-    C9-   C10
    20    22    23    27   3   4.83252  -7.65254   1.68196   1.13805   0.00000   0.00000 ; dih    N1-    C9-   C10-   C11
    22    23    27    30   3  -4.96013   6.28646   1.30959  -2.63592   0.00000   0.00000 ; dih    C9-   C10-   C11-   C12
    23    27    30    33   3   2.92880  -1.46440   0.20920  -1.67360   0.00000   0.00000 ; dih   C10-   C11-   C12-   C13
    27    30    33    36   3   2.92880  -1.46440   0.20920  -1.67360   0.00000   0.00000 ; dih   C11-   C12-   C13-   C14
    30    33    36    38   3   0.52719  -6.39734  -1.69452   7.56467   0.00000   0.00000 ; dih   C12-   C13-   C14-   C15
    33    36    38    40   3  58.57600   0.00000 -58.57600   0.00000   0.00000   0.00000 ; dih   C13-   C14-   C15-   C16
    36    38    40    42   3   0.52719  -6.39734  -1.69452   7.56467   0.00000   0.00000 ; dih   C14-   C15-   C16-   C17
    38    40    42    43   3   0.76567   2.29701   0.00000  -3.06269   0.00000   0.00000 ; dih   C15-   C16-   C17-   H25
    38    40    46    47   3   0.76567   2.29701   0.00000  -3.06269   0.00000   0.00000 ; dih   C15-   C16-   C18-   H22

[ dihedrals ]
; ai  aj   ak  al  funct    phi0          cp      mult
     1     3     9     2   1     180.000       4.602   2        ; imp     C1-    C2-    C6-   H12
     3     1     4    17   1     180.000       4.602   2        ; imp     C2-    C1-    C3-    C8
     4     3     6     5   1     180.000       4.602   2        ; imp     C3-    C2-    C4-   H13
     6     4     8     7   1     180.000       4.602   2        ; imp     C4-    C3-    C5-   H14
     8     6     9    15   1     180.000       4.602   2        ; imp     C5-    C4-    C6-    O2
     9     1     8    10   1     180.000       4.602   2        ; imp     C6-    C1-    C5-    O1
    22    20    23    26   1     180.000      43.932   2        ; imp     C9-    N1-   C10-    O3
    36    33    38    37   1     180.000      62.760   2        ; imp    C14-   C13-   C15-    H3
    38    36    40    39   1     180.000      62.760   2        ; imp    C15-   C14-   C16-    H1
```
