# Supplementary material for: Capsaicin Interaction with TRPV1 Channels in a Lipid Bilayer: Molecular Dynamics Simulation
Source: Biophys J. 2015 Mar 24;108(6):1425–34. doi: 10.1016/j.bpj.2015.02.013 (PMC4375533; doi:10.1016/j.bpj.2015.02.013)
Supplement: Document S2. Article plus Supporting Material [file mmc2.pdf]

Biophysical Journal Volume 108 March 2015 1425–1434

Article

Capsaicin Interaction with TRPV1 Channels in a Lipid Bilayer: Molecular Dynamics Simulation

Sonya M. Hanson,[1,2,3] Simon Newstead,[1] Kenton J. Swartz,[3] and Mark S. P. Sansom[1,*]
[1]Department of Biochemistry, University of Oxford, Oxford, United Kingdom; [2]Computational Biology Program, Memorial Sloan-Kettering Cancer Center, New York, New York; and [3]Molecular Physiology and Biophysics Section, Porter Neuroscience Research Center, National Institute of Neurological Disorders and Stroke, National Institutes of Health, Bethesda, Maryland

ABSTRACT Transient receptor potential vanilloid subtype 1 (TRPV1) is a heat-sensitive ion channel also involved in pain sensation, and is the receptor for capsaicin, the active ingredient of hot chili peppers. The recent structures of TRPV1 revealed putative ligand density within the S1 to S4 voltage-sensor-like domain of the protein. However, questions remain regarding the dynamic role of the lipid bilayer in ligand binding to TRPV1. Molecular dynamics simulations were used to explore behavior of capsaicin in a 1-palmitoyl-2-oleoyl phosphatidylcholine bilayer and with the target S1–S4 transmembrane helices of TRPV1. Equilibrium simulations reveal a preferred interfacial localization for capsaicin. We also observed a capsaicin molecule flipping from the extracellular to the intracellular leaflet, and subsequently able to access the intracellular TRPV1 binding site. Calculation of the potential of mean force (i.e., free energy profile) of capsaicin along the bilayer normal confirms that it prefers an interfacial localization. The free energy profile indicates that there is a nontrivial but surmountable barrier to the flipping of capsaicin between opposing leaflets of the bilayer. Molecular dynamics of the S1–S4 transmembrane helices of the TRPV1 in a lipid bilayer confirm that Y511, known to be crucial to capsaicin binding, has a distribution along the bilayer normal similar to that of the aromatic group of capsaicin. Simulations were conducted of the TRPV1 S1–S4 transmembrane helices in the presence of capsaicin placed in the aqueous phase, in the lipid, or docked to the protein. No stable interaction between ligand and protein was seen for simulations initiated with capsaicin in the bilayer. However, interactions were seen between TRPV1 and capsaicin starting from the cytosolic aqueous phase, and capsaicin remained stable in the majority of simulations from the docked pose. We discuss the significance of capsaicin flipping from the extracellular to the intracellular leaflet and mechanisms of binding site access by capsaicin.

INTRODUCTION

Transient receptor potential (TRP) channels are a diverse set of nonselective cation channels found in eukaryotic cells. These channels are often involved in sensory transduction, participating in the direct detection of stimuli ranging from osmotic sensing to temperature change, and are crucial members of lipid-based signaling pathways (1,2). Among the diversity of TRP channels are the receptors for molecules such as menthol, camphor, mustard, and capsaicin, the latter being responsible for the pungent hot nature of chili peppers (3). Capsaicin specifically activates the heat-sensitive transient receptor potential vanilloid subtype 1 (TRPV1) ion channel (4). Along with other members of the TRP channel family, known to play key roles in temperature sensation and other sensory functions (5), TRPV1 is a prominent therapeutic target (6,7). As capsaicin is a lipophilic molecule (Fig. 1 *A*) and the TRPV1 channel is an integral membrane protein with an overall transmembrane (TM) architecture resembling that of voltage-gated potassium (Kv) channels (8), it is likely that interactions with the membrane environment play a key role in mediating the effect of capsaicin. It is therefore important to understand those interactions in molecular detail.

Application of capsaicin to sensory neurons increases membrane conductance to cations, and was a valuable tool to early studies of nociceptive neurons (9). The capsaicin molecule may be divided into three functional groups: (A) the substituted aromatic region or vanillyl group, (B) the dipolar amide-bond region, and (C) the hydrophobic tail region (Fig. 1 *A*). The effects of modifications to each of these groups was well explored by the early nineties, for example, removal of the 4′-OH in the A group removes capsaicin activity, whereas the most important characteristic of the C group acyl chain tail is its hydrophobicity as it tolerates variations from as few as five carbons to as many as 15 without extensively modifying activity (10). Although these studies gave clues regarding tolerated modifications to capsaicin, the hydrophobicity of the capsaicin molecule and its derivatives make quantitative direct binding studies to the TM TRPV1 protein difficult. As a result resiniferatoxin (RTX), a compound from Moroccan cactus with higher affinity to TRPV1, has been used to perform radioligand binding experiments (11).

Submitted August 6, 2014, and accepted for publication February 3, 2015.
*Correspondence: mark.sansom@bioch.ox.ac.uk
This is an open access article under the CC BY license (http://creativecommons.org/licenses/by/4.0/).
Editor: Jose Faraldo-Gomez.
© 2015 The Authors
0006-3495/15/03/1425/10 $2.00
http://dx.doi.org/10.1016/j.bpj.2015.02.013

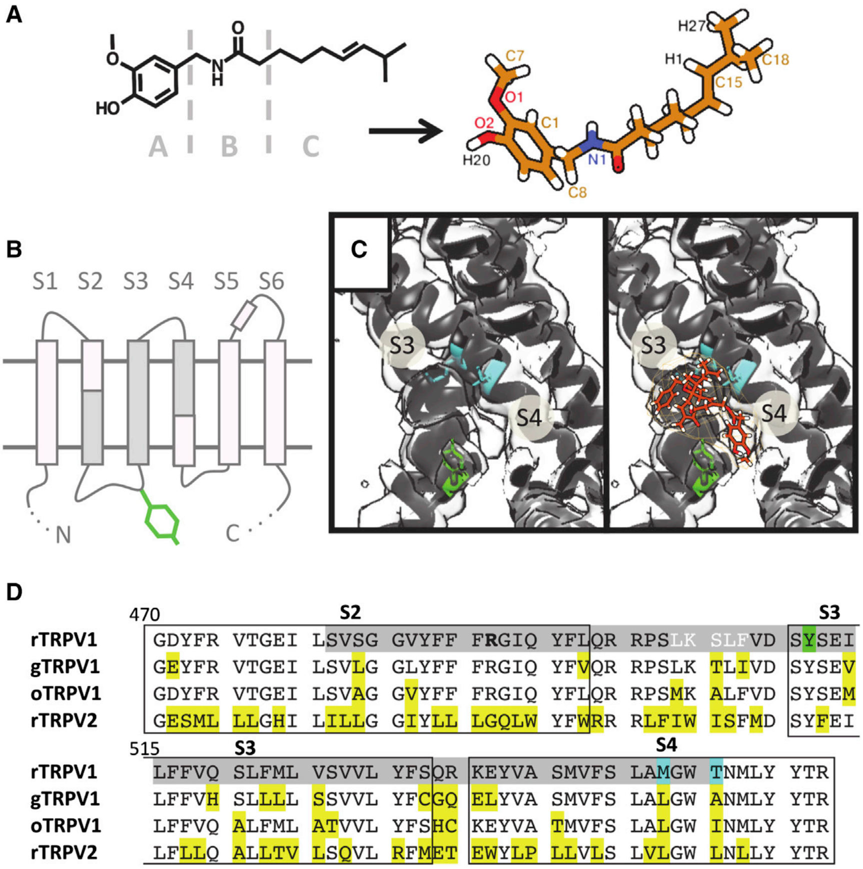

FIGURE 1 Capsaicin and TRPV1. (*A*) Capsaicin is a hydrophobic molecule that may be divided into three functional groups: the A group aromatic head with hydrogen bonding potentiality, the B group dipolar amide-bond region, and the C group hydrophobic tail. (*B*) The TM region of TRPV1 is composed of six TM helices, S1–S6, of which S5 and S6 form the pore domain, and the S2–S4 helices (*in gray*) are predicted to contain the capsaicin binding site, including the tyrosine 511, indicated in green. (*C*) A cryo-EM structure (PDB ID: 3J5Q) solved with both RTX and DkTx bound, shows density near the expected RTX and capsaicin-binding site (14). Residues known to be significant in RTX binding are colored as in (*D*). (*D*) Sequence alignment of rat (r), chicken (g), and rabbit (o) TRPV1 and rat (r) TRPV2, highlighting in gray the region thought to be physiologically significant (11,12). The tyrosine 511 is highlighted in green. M547 and T550 are also highlighted in blue as significant to ligand binding in TRPV1 (13). The region of the disordered S2–S3 loop is shown in white. Differences from the rat sequences are highlighted yellow. To see this figure in color, go online.

TRP channels belong to the tetrameric 6-TM superfamily of ion channels, which are composed of the pore-forming S5–S6 TM helices and an outer domain formed by the S1–S4 TM helices, which constitute the voltage-sensing domain (VSD) in canonical voltage-gated channels (Fig. 1 *B*). The VSD-like domain of TRP channels does not contain the positively charged amino acids in the S4 helix characteristic of voltage-sensitive channels. However, mutagenesis and related studies have revealed that residues found crucial to capsaicin and RTX binding are within the S2–S4 helices of TRPV1 (12–14) (see Fig. 1 *D*), thus implicating the VSD-like domain in the overall activation mechanism of the channel. In particular, mutating a tyrosine residue on the cytoplasmic side of the S3 helix (Y511) (Fig. 1 *B*) to alanine removes capsaicin sensitivity while the channel retains sensitivity to pH and heat (12). Channel activation by the endogenous ligand anandamide, released during tissue injury, is also lost in this mutant. Several mutagenesis studies have confirmed Y511's role in vanilloid binding to TRPV1, and also identified residues crucial to RTX binding in the middle of the S4 helix (13) (see Fig. 1 *D*).

Recently, the structure of rat TRPV1 at 3.4 Å resolution has confirmed that these residues lie within the lipid bilayer (Fig. 1 *C*), in line with the suggestion of a TM-binding site for TRPV1 ligands (8). Structures of TRPV1 with agonists bound (14) revealed both changes in the central pore domain upon ligand application as well as the location of the bound agonist molecules. Although the electron density attributed to RTX and to capsaicin near the S2–S4 helices was not sufficiently detailed to resolve their exact orientation and interactions, the RTX density was consistent with an interpretation that its equivalent to the A group of capsaicin interacts with the Y511 near the water-bilayer interface on the inner (i.e., cytosolic) side of the membrane (Fig. 1 *C*) (14).

Capsaicin is a relatively hydrophobic compound and a number of biophysical studies have revealed effects of capsaicin on model lipid bilayers ranging from pore formation (15), to effecting the phase transition temperature of lipids (16), to increasing bilayer fluidity (17). Additionally, a number of studies have highlighted the experimental issues associated with exploring the mode of action of hydrophobic compounds. For example, one study showed threefold higher potency upon longer preincubation with the ligand (18) and another identified a positive correlation between lipid partition coefficients and the pungency of TRPV1 agonists (19). Furthermore, there remains a lack of clarity as to whether capsaicin acts via the outer (i.e., extracellular) or inner (i.e., cytosolic) side of the cell membrane. Although capsaicin and RTX can both activate the channel when applied from either side of the membrane, a charged analog of capsaicin only activates TRPV1 when

applied intracellularly (20). Several other experiments, however, have also approached this question with conflicting results (20–24).

The surprisingly diverse consequences of the incomplete understanding of the role of the lipid bilayer in the interaction of capsaicin and its analogs with TRPV1 lead to a need for a higher resolution picture of the three elements of the system. Molecular dynamics (MD) simulation studies can reveal details of the interactions between hydrophobic molecules and lipid bilayers (25–34), and between lipids and integral membrane proteins (35–37). Previous relevant studies of the TRPV1-capsaicin interaction include simulations of capsaicin in an octanol-water system (38), as well as a docking study of capsaicin to a TRPV1 homology model (39). Neither of these studies directly addresses the role of the phospholipid bilayer in the interaction of capsaicin and TRPV1. However, simulation studies of other lipid-like molecules have explored their interactions with a lipid bilayer, addressing the issue of flip-flop from one bilayer leaflet to the other (40–42). These studies provide a possible protocol for using simulations to study how externally applied capsaicin reaches a cytosolic-facing binding site.

In this work, we explore the mechanistic importance of the lipid bilayer in ligand binding to TRPV1. By combining submicrosecond simulations of capsaicin in a phospholipid bilayer, both in the absence and the presence of the S1–S4 domain of TRPV1, with calculations of the free energy landscape for capsaicin translation along the bilayer normal, we arrive at a plausible model of how capsaicin accesses its binding site on the cytosolic face of the cell membrane. We find that the distribution of capsaicin in a lipid bilayer aligns the aromatic A group with the carbonyls of the lipid, consistent with the location of the comparable functional group of RTX in recent structural studies. Additionally, we propose the significance of capsaicin flip-flop from the extracellular to the intracellular side of the bilayer to access its binding site on TRPV1.

MATERIALS AND METHODS

Simulations of capsaicin with a 1-palmitoyl-2-oleoyl phosphatidylcholine (POPC) bilayer with or without protein were performed and analyzed using the GROMACS 4.5.5 (www.gromacs.org) (43) using the OPLS force field (44). Each simulation contained four capsaicin molecules, corresponding to 13 mM capsaicin. For each simulation the capsaicin molecules were initially placed at the center of each of the four *x-y* quadrants of the simulation box, either in the aqueous phase at a distance of 30 Å from the center of the bilayer, or at the center of the bilayer. To introduce the capsaicin molecule to the system, a slow-growth approach was used to transform a noninteracting molecule to a fully interacting molecule over a period of 0.5 ns. After this setup procedure 2 × 100 ns and 1 × 500 ns simulations were conducted with capsaicin in the aqueous starting position, and 1 × 500 ns simulation were conducted with capsaicin in the lipid starting position, each simulation containing four capsaicin molecules (see Table 1). A control POPC bilayer with no capsaicin molecules present was simulated for 500 ns.

Each simulation box contained 256 POPC molecules to form the bilayer, which was initially created using coarse-grained self-assembly methods (45). This bilayer was then converted to an atomistic representation. The final simulation box was of dimensions ~93 Å × 93 Å × 99 Å and contained 16893 TIP4P waters and NaCl at a concentration of ~230 mM. The system was maintained at a constant temperature of 323 K and a constant pressure of 1 atm using semiisotropic coupling with the Berendsen algorithm (46). Capsaicin was parameterized in the OPLS all-atom force field using topolbuild version 1.3 for initial parameterization along with an in-house script top-fill. The similarity of functional group in capsaicin to those present in peptides meant no subsequent manual curation was necessary (see the Supporting Material for the parameters used).

TABLE 1 Summary of simulations

Number of capsaicin and initial location	Replicas and duration (ns)	Protein
4× aqueous	2 × 100 and 1 × 500	–
4× lipid	1 × 500	–
none (control)	1 × 500	–
1 Å spaced windows along *z* for PMF	76 × 10	–
4× aqueous	4 × 50 and 2 × 100	rV1 S1–S4 (3J5P)
4× lipid	4 × 50 and 2 × 100	rV1 S1–S4 (3J5P)
4× lipid (all at intracellular leaflet)	6 × 50	rV1 S1–S4 (3J5P)
1× docked to Y511 site	3 × 50	rV1 S1–S4 (3J5R)
none (control)	3 × 100	rV1 S1–S4 (3J5P)

Umbrella sampling simulations employed the *z* axis (as an approximation to the bilayer normal) as the reaction coordinate, with windows spaced at 1 Å intervals. These simulations were conducted using GROMACS 4.6.1. An umbrella potential using a force constant of 1000 kJ mol^{-1} nm^{-2} was applied to the center of mass of the capsaicin molecule using the pull geometry cylinder method (27). Production simulations were run for 10 ns/window, of which the last 5 ns were used to generate a potential of mean force (PMF) profile using the weighted histogram analysis method (47). The profile was symmetrized and errors were calculated by the bootstrapping method (48).

Simulations including protein were conducted using a similar setup to those without protein, but including the S1–S4 helix domain of the apo structure of TRPV1 (Protein Data Bank (PDB) ID: 3J5P; 3.4 Å resolution) (8). The unresolved loop between the S2 and S3 helices (represented in *white lettering in the sequence alignment* in Fig. 1 *C*) was modeled in using Modeler version 9.10 (49). For simulations in which capsaicin was added to the aqueous phase, the S1–S4 TM region was initially embedded into the bilayer using a coarse-grained self-assembly approach, and subsequently converted into an atomistic representation. In a separate set of simulations, the g_membed (50) tool was used to insert the protein into a bilayer already containing four capsaicin molecules in the intracellular leaflet. Overall 4 × 50 ns and 2 × 100 ns simulations each were conducted to explore the capsaicin-S1–S4 interaction from the aqueous and lipid phase, and 6 × 50 ns simulations were conducted to explore possible interactions of the capsaicin molecules already on the intracellular leaflet with the TRPV1 S1-S4 helices. Three 100 ns simulations were also run of just the S1–S4 protein in the membrane without any capsaicin (see Table 1).

To further investigate the interaction between capsaicin and TRPV1 within the context of an explicit bilayer, simulations were conducted starting with capsaicin in a docked pose on TRPV1 S1–S4. Docking in the absence of a bilayer was performed with AutoDock Vina (51) on all three full-length TRPV1 structures (PDB ID: 3J5P, 3J5R, and 3J5Q). Simulations, however, were only started from the top-ranked capsaicin pose docked to 3J5R, as this was deemed the most physiological starting point. The docked capsaicin and the S1–S4 helices of 3J5R were inserted into a POPC membrane using the g_membed tool and 3 × 50 ns simulations were run. Analysis of these simulations was conducted using the gromacs clustering algorithm (52) and the volmap analysis tool in VMD. Images were generated using PyMOL (53), VMD (54), and Chimera (55).

RESULTS

Capsaicin localization

Capsaicin is relatively lipophilic as evidenced by an octanol-water partition coefficient of ~3.8, just above that of *n*-propylbenzene (56,57). In three simulations starting with four capsaicin molecules in the aqueous solution adjacent to a phospholipid bilayer (see Table 1), spontaneous insertion of most of the capsaicin molecules (83%) into the lipid bilayer occurred within the first 100 ns of simulation (Fig. 2). In some cases (four) individual capsaicin molecules are inserted into the bilayer, although in other cases either two (one instance with two capsaicin molecules) or four (one instance with four capsaicin molecules) capsaicin molecules formed an aggregate in the aqueous phase before insertion. However, even when the capsaicin molecules are inserted as an aggregate the capsaicin molecules subsequently disaggregated (laterally) following insertion into the bilayer. In Fig. 2 the trajectories along the bilayer normal of the individual capsaicin molecules from these simulations are shown.

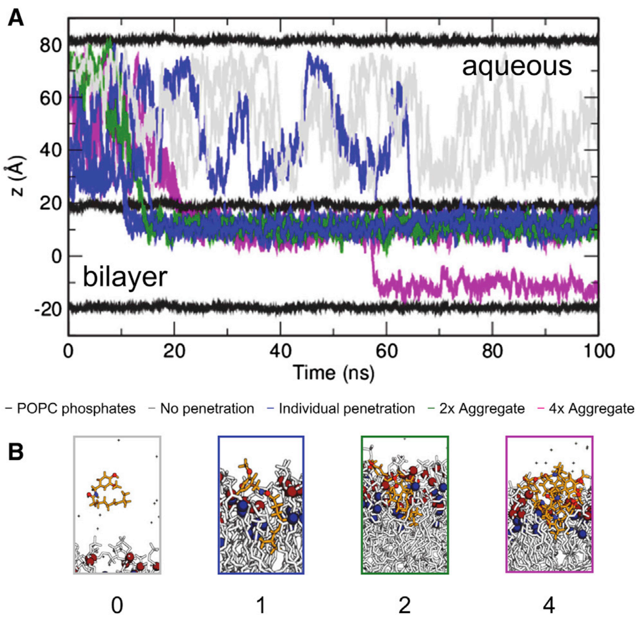

FIGURE 2 Overview of simulations of capsaicin in the presence of a POPC bilayer. (*A*) Capsaicin molecules penetrated the lipid bilayer spontaneously, though with differing pathways as shown in this plot of the center of mass of individual capsaicin molecules: molecules that inserted individually are shown in blue, molecules that inserted as aggregates of two are shown in green, and molecules that inserted as aggregates of four are shown in magenta. Molecules that remained in the aqueous phase are shown in gray. Each line represents an individual capsaicin molecule. The horizontal black traces correspond to the positions of the lipid bilayer phosphates. The topmost black line and the bottommost correspond to the same lipid bilayer phosphates, due to periodic boundary conditions. Note that the capsaicin molecules represented in this plot are from three separate simulations, and not all capsaicin molecules entered the bilayer from the same side, but have here been superposed as such for ease of conceptualization. (*B*) Snapshots of capsaicin and bilayer for failure to penetrate (0; *gray*), for an individually inserted molecule (1; *blue*), and for insertion as an aggregate of 2 (2; *green*), or 4 (4; *magenta*). To see this figure in color, go online.

Capsaicin molecules that formed aggregates before insertion subsequently diffused away from each other laterally within the bilayer. Their localization along the bilayer normal remained consistent through the remainder of the simulations, and was not distinguishable from that of capsaicin, which had inserted as individual (i.e., not aggregated) capsaicin molecules. The density distributions of capsaicin and of its A, B, and C groups along the bilayer normal can be compared with the location of the lipid headgroups and tails (Fig. 3). These density profiles show that the vanillyl group (A-region) interacts predominantly with the carbonyls of the lipid molecules. This is comparable to previously seen interactions of amphipathic aromatic (i.e., tyrosine and tryptophan) amino acid side chains of membrane proteins with these groups of lipids (30). An example of a long-lasting (>20 ns) H-bond interaction between a phospholipid carbonyl and capsaicin is shown in the Fig. S1.

Inspection of Fig. 2 reveals that one capsaicin molecule, originally in a 4× aggregate formed in the aqueous phase before insertion (shown by the *magenta line*), flips from one leaflet of the bilayer to the other. To explore possible capsaicin flip-flops further, two simulations of four capsaicin molecules each were extended to 500 ns, resulting in a total of over 4 μs of individual capsaicin trajectories in a lipid bilayer. A similar event, however, did not reoccur. In

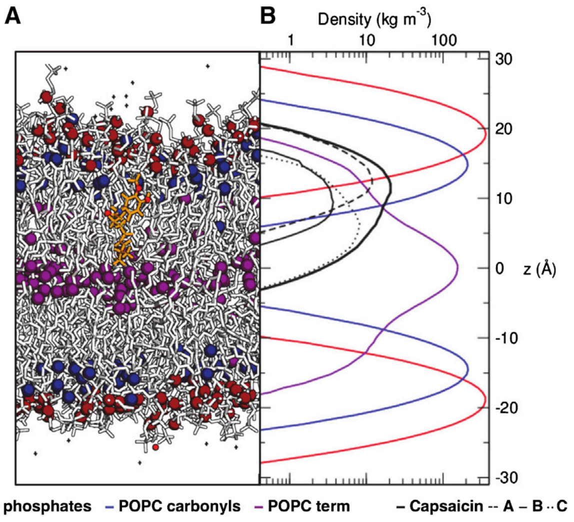

FIGURE 3 Localization of capsaicin within a model bilayer. During the course of simulations the location of capsaicin remained consistent (*A*) once it penetrated the bilayer. Here, a partial density profile (*B*) of 30 ns of simulation shows how capsaicin sits within a POPC bilayer. POPC phosphates, carbonyls, and terminal methyls are shown in red, blue, and purple, respectively. The capsaicin molecule is shown as a thick black line, and the distribution of the A, B, and C groups are shown as dashed, straight, and dotted lines, respectively. The A-group containing the phenol moiety aligns, on average, with the carbonyls of the POPC, whereas the rest of the molecule extends toward the center of the bilayer. To see this figure in color, go online.

Fig. 4 *A* the observed flip is shown in atomistic detail via snapshots that represent the molecule before and after the flip-flop transition (Fig. 4 *B*). Additional simulations (re) started at different time points along this transition (see *vertical arrows* in Fig. 4 *B*) suggest that it may correspond to passage over a local maximum (i.e., barrier) in a free energy landscape in that the capsaicin molecule could either flop back to its initial (i.e., extracellular) leaflet location or flip to its new (i.e., cytosolic) location (see Fig. S2 for the trajectories of capsaicin in these (re)started simulations). The presence of a local energy barrier would also explain the low frequency (once in 4 μs total of simulation) with which such a flip was observed.

Free energy profile

To provide a more quantitative description of the energy landscape underlying capsaicin movement across a lipid bilayer we have calculated the PMF for translation of a capsaicin molecule along the *z* axis, i.e., along the approximate bilayer normal. The PMF for capsaicin crossing a POPC bilayer was obtained by umbrella sampling using 10 ns restrained simulations for 1 Å windows along *z*. Thus, the resulting PMF provides a free energy profile for translation of a single capsaicin molecule from one side to the other of the bilayer (Fig. 5). This profile (see Fig. 5 *B*) is in good agreement with the distribution of capsaicin molecules seen in the earlier standard MD simulations (Fig. 5 *A*).

It is informative to consider errors in the estimates of the PMF. To this end one may compare the symmetrized (Fig. 5 *B*) and unsymmetrized (Fig. 5 *C*) PMFs. Both reveal a similar free energy profile, with energy minima at the water/lipid headgroup interface, and a modest energy barrier at the center of the bilayer. An approximate estimate of the height of the barrier relative to the minima with capsaicin at the preferred interfacial location may be obtained from the relative densities in those two regions, and compared to the estimates from the two PMFs. This gives estimates

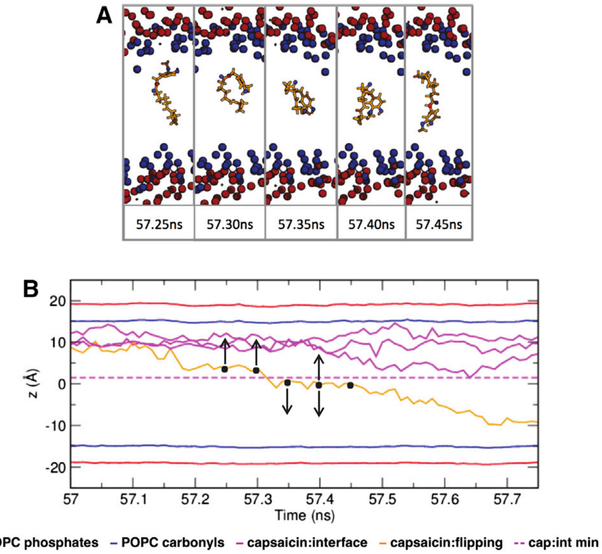

FIGURE 4 Capsaicin was seen to flip spontaneously from one bilayer to the other. (*A*) Snapshots of the flip-flop event. (*B*) Distance of the center of mass of a capsaicin molecule relative to the bilayer center versus time providing a more detailed view of the single capsaicin that flips from one bilayer to the other. POPC phosphates are shown in red, whereas carbonyls are shown in blue. Capsaicin molecules that did not flip are shown in magenta, with their closest approach to the center of the bilayer shown as a dotted magenta line. Snapshots of the flipping capsaicin were taken at either side of this line, indicated by black dots. The arrows indicate the directions of movement capsaicin in additional simulations (re)started at different time points along this transition (see main text and Fig. S2 for details). To see this figure in color, go online.

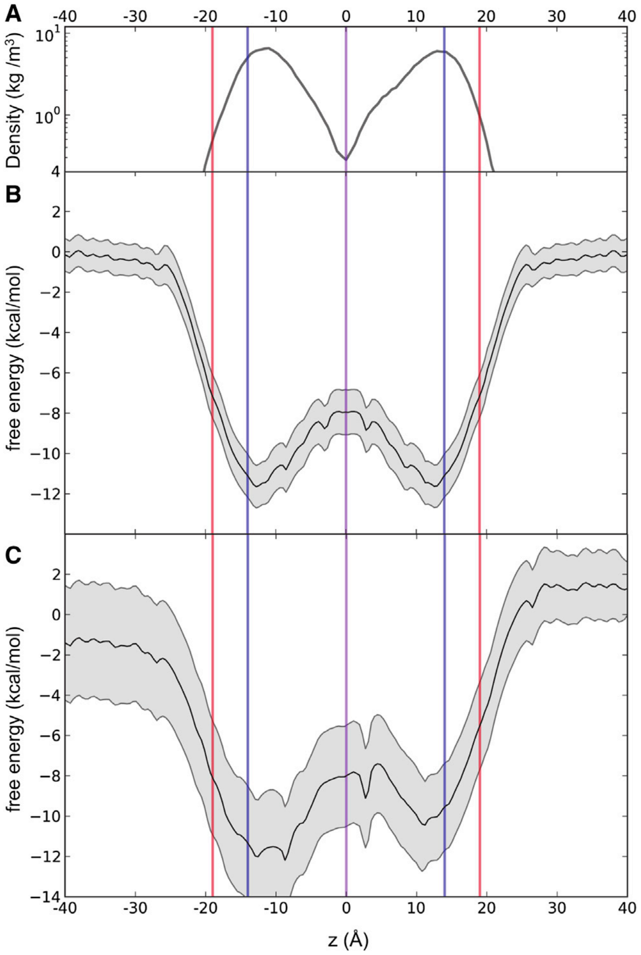

FIGURE 5 Potential of mean force of capsaicin along the normal of a POPC bilayer. (*A*) The partial density averaged over 50 ns for two capsaicin molecules, initially positioned one in each leaflet of the bilayer (see Fig. 2) compared to the (*B*) symmetrized and (*C*) unsymmetrized PMF profiles, as calculated from the last 5 ns of each 10 ns umbrella restrained simulations at 1 Å windows along the *z* axis, with the free energy set to zero in the aqueous phase. The gray region either side of the PMFs represents the standard deviation on either side of the average profile as calculated by bootstrapping. Vertical lines indicate the mean positions along *z* of the phosphates (*red*), carbonyls (*blue*), and terminal methyls (*purple*) of the lipid molecules. To see this figure in color, go online.

of the free energy barrier at the center of the bilayer of ~6 kT from the symmetrized PMF (Fig. 5 *B*), of ~3 to ~7 kT from the unsymmetrized PMF (Fig. 5 *C*), and of ~3 kT from the density profile (Fig. 5 *A*).

The resulting free energy profile also matches well with previous calculations of related PMFs, such as of those previously calculated for the amino acids tyrosine and leucine (30). Because there is no barrier to bilayer insertion and a small but nontrivial barrier (~6 kT) for crossing the hydrophobic core, it is in agreement with the qualitative results of the unrestrained simulations discussed previously, and provides a more quantitative view of barriers capsaicin faces in accessing the potential TRPV1 binding site on the cytosolic face of the cell membrane.

Capsaicin, with a 10-carbon acyl chain tail (the *C-group* in Fig. 1 *A*), is a relatively large compound with which to conduct free energy calculations across a lipid bilayer. We therefore had some concerns as to whether 10 ns for each simulation window in the umbrella sampling calculations would be sufficient to provide a reasonable, albeit approximate, estimate of the underlying PMF. We were encouraged that a consistent profile indicative of convergent behavior was seen when comparing PMF profiles calculated from subsections of the trajectories (see e.g., Fig. S3). We were also encouraged by the close correspondence between the PMF profile and the distribution of capsaicin molecules from standard MD simulations (Fig. 5 *A*). Examination of an unsymmetrized PMF (Fig. 5 *C*) suggested an error of ~1 kcal/mol in estimation of well depths and barrier heights. Thus, the depth of the energy well at the bilayer interface relative to the reference bulk aqueous phase was -10 kcal/mol for z $= -12$ Å and -11.5 kcal/mol for z $= +12$ Å in the unsymmetrized PMF, compared with -11 kcal/mol for $|z| = 12$ Å in the symmetrized PMF (Fig. 5). However, we are aware that a number of studies have emphasized the importance of running extended simulations and multiple replicas to achieve a fully converged PMF. We note that several of these studies focus on molecules (e.g., antimicrobial peptides) that significantly perturb the bilayer (27,58,59). In contrast, capsaicin (a relatively lipophilic compound) did not significantly perturb the bilayer (see e.g., Figs. 2 *B* and 4 *A*). Thus, although extended simulation times per window would doubtless improve the convergence of the PMF, we remain confident that 10 ns windows (32) provide a reasonable first estimate of the free energy landscape experienced by capsaicin in a bilayer.

Adding the S1–S4 TM helix domain

As described previously, capsaicin has been suggested to interact with Y511 at the intracellular side of the S3 TM helix. To investigate how the position of this tyrosine relative to the lipid bilayer compared to the localization of capsaicin within the membrane, we performed simulations of the electron microscopy (EM) structure of the S1–S4 helices of TRPV1 in a POPC bilayer. The partial density profile (Fig. 6) reveals that Y511 is located in the interfacial region of the bilayer at $z \sim -15$ Å, close to the lipid carbonyl groups. This corresponds exactly to the minimum energy location of the capsaicin vanillyl group on the intracellular face of the bilayer (Fig. 3).

We note that despite being isolated from the rest of the full-length TRPV1 channel structure, the S1–S4 helix domain remained relatively stable throughout the simulation (Fig. S4 *A*), as has also been seen in comparable simulations, e.g., of the isolated VSD of Kv channels (60,61). Interestingly, the side chain of Y511 in these simulations was seen to spontaneously flip from the outward-facing orientation, seen in the structure of ligand-free TRPV1, to the inward position seen in the RTX-bound and capsaicin-bound structures (Fig. S4 *B*), even though no ligand was present in the simulations.

Further simulations (Fig. 7) of the S1–S4 helices were conducted to include capsaicin either added via the aqueous solution or via insertion into the bilayer. Six 50 or 100 ns duration simulations were conducted for each capsaicin starting position, aqueous phase or lipid. No stable interactions were seen between the capsaicin molecules within the bilayer and the protein. Further simulations (6×50 ns) were even conducted using a starting position of all four capsaicin molecules on the Y511 side of the bilayer, with no resulting significant interactions. However, significant capsaicin-TRPV1 interactions occurred in four of the simulations initiated with capsaicin in the aqueous solution (Fig. S5), and all of these interactions occurred at the intracellular side of the S1–S4 helices.

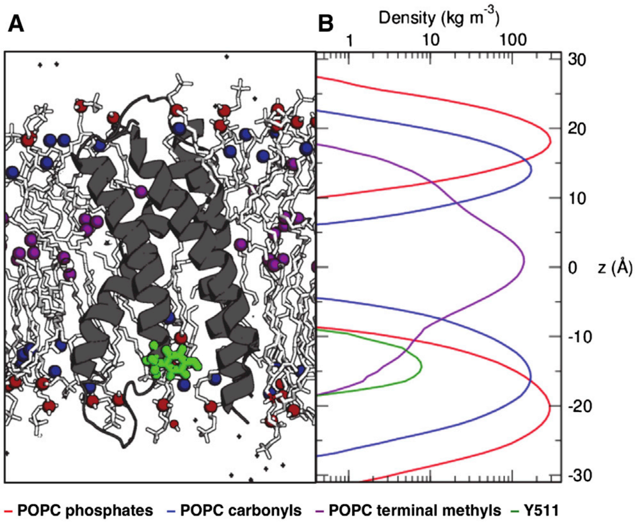

FIGURE 6 Localization of Y511 of the TRPV1 S1-S4 domain within a POPC bilayer. (*A*) Simulations of the S1–S4 domain from the TRPV1 structure provided an estimate of the location within a lipid bilayer of Y511 (*B*), which is known to play a key role in capsaicin binding. POPC phosphates, carbonyls, and terminal methyls are shown in red, blue, and purple, respectively. The tyrosine is seen to localize alongside the carbonyls of the lipids, similar to the A-group of the capsaicin. To see this figure in color, go online.

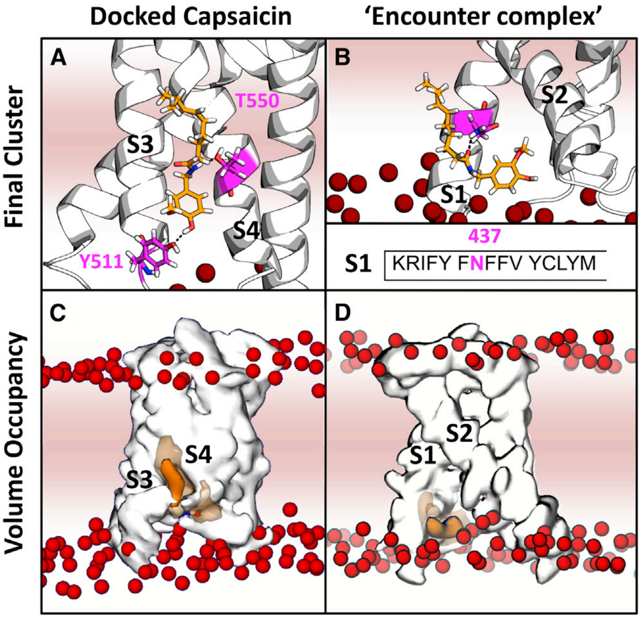

FIGURE 7 Simulations of TRPV1 S1–S4 with capsaicin. (*A*) The final cluster from analysis of one of the simulations (sim2) starting from a docked capsaicin pose (see Fig. S7 for further details). Stabilizing hydrogen bonding interactions are seen between capsaicin and the side chains of residues Y511 and T550 (*in magenta*). This indicates that capsaicin may remain in a stable bound position with the A group aromatic region facing toward the intracellular phospholipid carbonyls. (*B*) The final cluster from one of the simulations with capsaicin initially in the aqueous phase, revealing that capsaicin interacts with the TRPV1 S1-S4 domain (see Figs. S5 and S6 for further details). The majority of these interactions involved capsaicin initially encountering the S1 helix in the aqueous phase, before ending up fully embedded in the bilayer. An interaction with asparagine 437 (*magenta*) was seen in these simulations. (*C*) Volumetric density map (from VMD, *capsaicin density in orange*, *protein density in white*) over the course of the 50 ns simulation in which capsaicin was initially docked to the TRPV1 S1–S4 (sim2, see *A*). (*D*) The volumetric density map over the course of the 100 ns simulation (see *C*) in which capsaicin was seen to interact with the TPV1 S1-S4 from the aqueous phase. In all four figures POPC phosphates are represented as dark red spheres and the middle of the bilayer as darker pink shading. To see this figure in color, go online.

One set of capsaicin-TRPV1 interactions involved an aggregate of two capsaicin molecules that interacted for ~20 ns with the intracellular S2–S3 loop before diffusing away (Fig. S5 *D*). Other interactions involved capsaicin molecules interacting with the protein while entering the bilayer until they became fully embedded (Fig. S5, *A–C*). In three of these instances, the interaction was with the S1 of the protein, rather than the S2–S4 (Fig. 7 *D*). Additionally, in the two 100 ns simulation in which this interaction persisted for 90–100 ns, this interaction was with the same asparagine of the S1 (Fig. 7 *B*). This interaction increasingly stabilized over the duration of the simulation (Fig. S6), and though the interaction was unexpected, the final localization was not far from the tyrosine 511 and the EM density for RTX with a minimum distance from the capsaicin molecule to the Y511 side chain of 11.6 Å, though no specific interactions with Y511 were observed.

To further investigate the capsaicin-TRPV1 interaction within the bilayer, simulations of the S1–S4 of TRPV1 were started from a docked pose of capsaicin, in which it was seen to interact with the S1–S4 helices in line with the current predominant model of capsaicin binding, including hydrogen bonding to Y511 and T550 (Fig. S7 *B*). Here, it was seen that this mode of binding was relatively stable, as 2 of 3 50 ns simulations maintained protein-ligand interactions (Fig. S7 *A*). In these simulations the hydrogen bonding network of the capsaicin-TRPV1 interaction was stabilized further and the location of the capsaicin between the S3 and S4 helices remained throughout the simulations (Fig. 7, *A* and *C*). Additionally, in the third simulation in which the capsaicin dissociates from the bound pose, capsaicin remained in the bilayer in the same manner as in the capsaicin/bilayer simulations described earlier (Fig. S7 *C*). Furthermore, if one overlays capsaicin after the 50 ns simulation from of one of these docked poses with the electron density map from the capsaicin-bound TRPV1 structure, an approximate overlap is seen between the A-group of the capsaicin and the density thought to correspond to capsaicin (Fig. S7 *F*).

DISCUSSION

Our results show the role of the lipid bilayer in capsaicin localization and of flip-flop when considering its binding to TRPV1. The capsaicin A region localizes alongside the phospholipid carbonyls, as does the Y511 of TRPV1. To access the Y511 side chain, externally applied capsaicin needs to flip from the extracellular leaflet to the intracellular leaflet, thereby crossing a barrier of ~6 kT as judged via the calculation of a PMF (free energy) profile. We have observed this flip occurs spontaneously, albeit rarely, in molecular detail in an unrestrained simulation.

More generally, our studies may be compared to a number of simulations of passive permeation of drugs across cell membranes (26,29,31–33). Although such investigations shed light on the involvement and significance of the lipid bilayer in the activation of TRPV1 by capsaicin and related molecules, it is important to integrate them with our understanding of the nature of the TRPV1 binding site for capsaicin and related ligands, as revealed in the recent cryo-EM structure. A number of questions remain. For example, it is known that aspects of RTX and capsaicin activation of TRPV1 differ despite the two ligands having overlapping binding sites, but to what extent these differences apply to the orientation, access pathways, and binding pocket of the molecules within TRPV1 remains unclear. In addition, the flip of the Y511 side chain between the apo- and ligand-bound structures of the protein, and our observation that this flip can occur spontaneously in simulations of the S1–S4 domain of the protein in a bilayer, opens the question of the exact role of this motion in ligand binding and/or channel activation.

It is possible that capsaicin could form an initial encounter complex with the TRPV1 molecule before binding more tightly in a manner mediated by the Y511 side chain conformational flip (62). Alternatively, it is possible that the absence of long-lived encounters between capsaicin and TRPV1 in the simulations imply that this ligand, in contrast to RTX, interacts only transiently with the channel. We note, for example, that amantadine has been suggested by solution NMR studies to interact with the exterior of the influenza M2 channel protein before binding to the blocking site within the channel seen in x-ray studies (63,64). This (somewhat speculative) interpretation of capsaicin interactions with TRPV1 would be in agreement with our observation of capsaicin interacting with the S1 TM helix. It would be of interest to investigate whether mutations of residues in the internal end of S1, such as N437, alter either the kinetics of binding or bilayer penetration of capsaicin.

Docking attempts to several regions of the TRPV1 structures, did not yield the encounter complex seen in simulations, perhaps due to the absence of a lipid bilayer during docking. Docking did however, yield a plausible canonical bound state involving the previously predicted S3 and S4 residues. Through simulations starting from this docked pose we were able to see that capsaicin could maintain a stable bound orientation with the A-region near the lipid carbonyls, similar to the simulations of capsaicin alone in the bilayer, though in this case stabilized by hydrogen bonding interactions to residues Y511 and T550. The simulation in which capsaicin dissociates from this docked pose also provides an insight into how capsaicin could access this binding site from the flipped orientation in the intracellular leaflet of the bilayer.

By combining interpretations derived from these simulations we are able to present a possible pathway for capsaicin binding to TRPV1 (Fig. 8), whereby capsaicin penetrates the bilayer before flipping from the extracellular to the intracellular leaflet to access the TM-binding site. Although this is not the only possible model, it agrees well both with our observations and with previous literature (12,39). Possible alternative models would suggest either that capsaicin could exit the bilayer on its cytosolic face before interacting with the cytosolic side of the protein to access the binding site or that capsaicin may interact directly with the protein (possibly at the protein lipid interface) to cross the bilayer and reach its final binding site. To understand the barrier presented by the bilayer per se to capsaicin flip-flop from the extracellular leaflet to the cytosolic leaflet (as proposed in our preferred model, see Fig. 8), we have calculated the PMF along the (approximate) bilayer normal. We believe the resulting energetic barrier to inform our discussion of the mode of action of capsaicin, while acknowledging possible limitations of the methodology employed.

A number of recent studies discuss calculations of drug PMF profiles across lipid bilayers, addressing the issues of convergence and of possible local membrane deformations (27,29,59) as well as the difficulties experienced when comparing calculations to experimentally relevant quantities (32) and the importance of increasing timescales and enhancing sampling for such calculations (29). However, our results are in reasonable agreement with previous PMF calculations of amino acids and small molecules across bilayers (33,35,63,65). In particular, the hydrophobic tail of capsaicin is not long enough nor is the molecule sufficiently charged to generate some of the bilayer deformation problems seen in, e.g., studies with antimicrobial peptides (58).

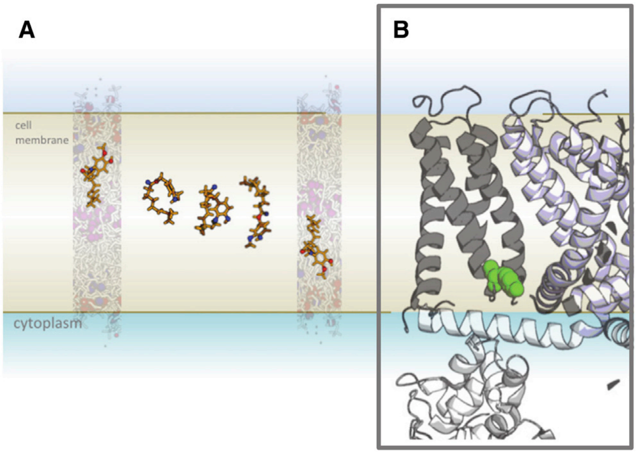

FIGURE 8 Proposed membrane-mediated interaction of capsaicin binding to TRPV1. Combining images from bilayer localization, capsaicin flip-flop from the extracellular to inner leaflet, the transbilayer PMF profile, and the bilayer localization of tyrosine 511, enables us to propose a possible mechanism of membrane-mediated capsaicin binding: (*A*) capsaicin penetrates the bilayer before flipping from the extracellular to the intracellular leaflet to access (*B*) the transmembrane binding site. To see this figure in color, go online.

One further possible limitation of our PMF calculations, and of many such calculations in general, is the use of a simple model bilayer (POPC), which fails to address the complexities of composition and asymmetry of cell membranes (66). Such aspects of bilayer composition have proven to be important, for example, in the regulation of TRPV1 by PIP_2 (67). A further likely limitation concerns our protein simulations, in particular the modest timescales sampled and the isolation of the S1–S4 domain from the full-length TRPV1 protein. Experiments have shown that residues in the N- and C-termini, and even the pore domain are required for capsaicin and RTX activation of TRPV1 (18,19). Thus, further simulations of capsaicin interactions with the full-length protein are desirable in the future. It is likely that calculation of a TRPV1/capsaicin PMF in a lipid bilayer would present issues of convergence and so might require the use of enhanced sampling techniques such as, e.g., metadynamics (68).

Generally, the interaction of lipophilic drugs such as capsaicin with membrane-embedded binding sites requires

the consideration of additional parameters to those considered in drug binding events in the aqueous phase. In particular, diffusion of the drug in the spatially inhomogeneous environment presented by cell membranes is likely to be of some importance in determining the kinetics of drug/receptor interactions in a membrane. Although the structures of TRPV1 have illuminated that the vanilloid site is in fact intracellular (10,17) and not almost extracellular as was posited in studies discussing RTX binding to TRPV1 (16,26), questions remain. For example, the involvement of the cytosolic domains, the source of the differences between RTX and capsaicin binding, the mechanism by which ligand binding induces channel opening, and the pathway these hydrophobic molecules take before binding the membrane-embedded binding site all require further investigation. The current study has helped to inform at least the last of these aspects of understanding how capsaicin binds to TRPV1, thus contributing to an improved understanding of how to better target TRPV1 with capsaicin-like compounds.

SUPPORTING MATERIAL

Seven figures and the capsaicin parameters are available at http://www.biophysj.org/biophysj/supplemental/S0006-3495(15)00176-9.

ACKNOWLEDGMENTS

We thank our colleagues, especially Dr. Andrés Jara-Oseguera, Dr. Chanhyung Bae, Dr. Jerome Ma, Dr. Dmitriy Krepkiy, Dr. Gilman Toombes, Dr. Greg Ross, Jean Hélie, and Dr. Phillip Stansfeld, for their advice and assistance.

This work was supported by grants from the Wellcome Trust and the BBSRC (to M.S.P.S.), from the MRC (G0900399) and the Wellcome Trust (102890/Z/13/Z) (to S.N.), and from the Intramural Research Program of the NINDS, NIH (to K.J.S.).

REFERENCES

1. Clapham, D. E. 2003. TRP channels as cellular sensors. *Nature.* 426:517–524.
2. Sisignano, M., D. L. H. Bennett, …, K. Scholich. 2014. TRP-channels as key integrators of lipid pathways in nociceptive neurons. *Prog. Lipid Res.* 53:93–107.
3. Dasgupta, P., and C. J. Fowler. 1997. Chillies: from antiquity to urology. *Br. J. Urol.* 80:845–852.
4. Caterina, M. J., M. A. Schumacher, …, D. Julius. 1997. The capsaicin receptor: a heat-activated ion channel in the pain pathway. *Nature.* 389:816–824.
5. Nilius, B., G. Owsianik, …, J. A. Peters. 2007. Transient receptor potential cation channels in disease. *Physiol. Rev.* 87:165–217.
6. Wong, G. Y., and N. R. Gavva. 2009. Therapeutic potential of vanilloid receptor TRPV1 agonists and antagonists as analgesics: Recent advances and setbacks. *Brain Res. Brain Res. Rev.* 60:267–277.
7. Gunthorpe, M. J., and B. A. Chizh. 2009. Clinical development of TRPV1 antagonists: targeting a pivotal point in the pain pathway. *Drug Discov. Today.* 14:56–67.
8. Liao, M., E. Cao, …, Y. Cheng. 2013. Structure of the TRPV1 ion channel determined by electron cryo-microscopy. *Nature.* 504:107–112.
9. Marsh, S. J., C. E. Stansfeld, …, D. McCarthy. 1987. The mechanism of action of capsaicin on sensory C-type neurons and their axons in vitro. *Neuroscience.* 23:275–289.
10. Wood, J. N. 1993. Capsaicin in the Study of Pain. *In* Capsaicin in the Study of Pain. J. N. Wood, editor. Academic Press, London, UK, pp. 1–286.
11. Szallasi, A., and P. M. Blumberg. 1990. Specific binding of resiniferatoxin, an ultrapotent capsaicin analog, by dorsal root ganglion membranes. *Brain Res.* 524:106–111.
12. Jordt, S.-E., and D. Julius. 2002. Molecular basis for species-specific sensitivity to "hot" chili peppers. *Cell.* 108:421–430.
13. Gavva, N. R., L. Klionsky, …, J. J. S. Treanor. 2004. Molecular determinants of vanilloid sensitivity in TRPV1. *J. Biol. Chem.* 279:20283–20295.
14. Cao, E., M. Liao, …, D. Julius. 2013. TRPV1 structures in distinct conformations reveal activation mechanisms. *Nature.* 504:113–118.
15. Feigin, A. M., E. V. Aronov, …, J. G. Brand. 1995. Capsaicin and its analogs induce ion channels in planar lipid bilayers. *Neuroreport.* 6:2134–2136.
16. Aranda, F. J., J. Villalaín, and J. C. Gómez-Fernández. 1995. Capsaicin affects the structure and phase organization of phospholipid membranes. *Biochim. Biophys. Acta.* 1234:225–234.
17. Lundbaek, J. A., P. Birn, …, O. S. Andersen. 2005. Capsaicin regulates voltage-dependent sodium channels by altering lipid bilayer elasticity. *Mol. Pharmacol.* 68:680–689.
18. Lazar, J., D. C. Braun, …, J. Lee. 2006. Kinetics of penetration influence the apparent potency of vanilloids on TRPV1. *Mol. Pharmacol.* 69:1166–1173.
19. Ursu, D., K. Knopp, …, E. Sher. 2010. Pungency of TRPV1 agonists is directly correlated with kinetics of receptor activation and lipophilicity. *Eur. J. Pharmacol.* 641:114–122.
20. Jung, J., S. W. Hwang, …, U. Oh. 1999. Capsaicin binds to the intracellular domain of the capsaicin-activated ion channel. *J. Neurosci.* 19:529–538.
21. Jung, J., S.-Y. Lee, …, U. Oh. 2002. Agonist recognition sites in the cytosolic tails of vanilloid receptor 1. *J. Biol. Chem.* 277:44448–44454.
22. Welch, J. M., S. A. Simon, and P. H. Reinhart. 2000. The activation mechanism of rat vanilloid receptor 1 by capsaicin involves the pore domain and differs from the activation by either acid or heat. *Proc. Natl. Acad. Sci. USA.* 97:13889–13894.
23. Chou, M. Z., T. Mtui, …, R. E. Middleton. 2004. Resiniferatoxin binds to the capsaicin receptor (TRPV1) near the extracellular side of the S4 transmembrane domain. *Biochemistry.* 43:2501–2511.
24. Vyklický, L., A. Lyfenko, …, V. Vlachová. 2003. Vanilloid receptor TRPV1 is not activated by vanilloids applied intracellularly. *Neuroreport.* 14:1061–1065.
25. Bemporad, D., C. Luttmann, and J. W. Essex. 2004. Computer simulation of small molecule permeation across a lipid bilayer: dependence on bilayer properties and solute volume, size, and cross-sectional area. *Biophys. J.* 87:1–13.
26. Cerezo, J., J. Zúñiga, …, J. P. Cerón-Carrasco. 2011. Atomistic molecular dynamics simulations of the interactions of oleic and 2-hydroxyoleic acids with phosphatidylcholine bilayers. *J. Phys. Chem. B.* 115:11727–11738.
27. Filipe, H. A. L., M. J. Moreno, …, L. M. S. Loura. 2014. How to tackle the issues in free energy simulations of long amphiphiles interacting with lipid membranes: convergence and local membrane deformations. *J. Phys. Chem. B.* 118:3572–3581.
28. Grossfield, A., and T. B. Woolf. 2002. Interaction of tryptophan analogs with POPC lipid bilayers investigated by molecular dynamics calculations. *Langmuir.* 18:198–210.
29. Loverde, S. M. 2014. Molecular simulation of the transport of drugs across model membranes. *J. Phys. Chem. Lett.* 5:1659–1665.

30. MacCallum, J. L., W. F. D. Bennett, and D. P. Tieleman. 2008. Distribution of amino acids in a lipid bilayer from computer simulations. *Biophys. J.* 94:3393–3404.

31. Marrink, S. J., and H. J. C. Berendsen. 1996. Permeation process of small olecules across lipid membranes studied by molecular dynamics simulations. *J. Phys. Chem.* 100:16729–16738.

32. Swift, R. V., and R. E. Amaro. 2013. Back to the future: can physical models of passive membrane permeability help reduce drug candidate attrition and move us beyond QSPR? *Chem. Biol. Drug Des.* 81:61–71.

33. Vijayan, R., and P. C. Biggin. 2008. A steroid in a lipid bilayer: localization, orientation, and energetics. *Biophys. J.* 95:L45–L47.

34. Wei, C., and A. Pohorille. 2011. Permeation of nucleosides through lipid bilayers. *J. Phys. Chem. B.* 115:3681–3688.

35. Stansfeld, P. J., E. E. Jefferys, and M. S. P. Sansom. 2013. Multiscale simulations reveal conserved patterns of lipid interactions with aquaporins. *Structure.* 21:810–819.

36. Schmidt, M. R., P. J. Stansfeld, …, M. S. Sansom. 2013. Simulation-based prediction of phosphatidylinositol 4,5-bisphosphate binding to an ion channel. *Biochemistry.* 52:279–281.

37. Arnarez, C., J.-P. Mazat, …, X. Periole. 2013. Evidence for cardiolipin binding sites on the membrane-exposed surface of the cytochrome bc1. *J. Am. Chem. Soc.* 135:3112–3120.

38. Lambert, J. W., and A. K. Sum. 2006. Molecular dynamics study of the properties of capsaicin in an 1-octanol/water system. *J. Phys. Chem. B.* 110:2351–2357.

39. Lee, J. H., Y. Lee, …, S. Choi. 2011. Structural insights into transient receptor potential vanilloid type 1 (TRPV1) from homology modeling, flexible docking, and mutational studies. *J. Comput. Aided Mol. Des.* 25:317–327.

40. Gurtovenko, A. A., and I. Vattulainen. 2007. Molecular mechanism for lipid flip-flops. *J. Phys. Chem. B.* 111:13554–13559.

41. Róg, T., L. M. Stimson, …, M. Karttunen. 2008. Replacing the cholesterol hydroxyl group with the ketone group facilitates sterol flip-flop and promotes membrane fluidity. *J. Phys. Chem. B.* 112:1946–1952.

42. Bennett, W. F. D., J. L. MacCallum, …, D. P. Tieleman. 2009. Molecular view of cholesterol flip-flop and chemical potential in different membrane environments. *J. Am. Chem. Soc.* 131:12714–12720.

43. Hess, B., S. Uppsala, and E. Lindahl. 2008. GROMACS 4: Algorithms for highly efficient, load-balanced, and scalable molecular simulation. *J. Chem. Theory Comput.* 4:435–447.

44. Jorgensen, W. L., D. S. Maxwell, and J. Tirado-Rives. 1996. Development and testing of the OPLS all-atom force field on conformational energetics and properties of organic liquids. *J. Am. Chem. Soc.* 118:11225–11236.

45. Stansfeld, P. J., and M. S. P. Sansom. 2011. From coarse grained to atomistic: a serial multiscale approach to membrane protein simulations. *J. Chem. Theory Comput.* 7:1157–1166.

46. Berendsen, H. J. C., J. P. M. Postma, …, J. R. Haak. 1984. Molecular dynamics with coupling to an external bath. *J. Chem. Phys.* 81:3684.

47. Kumar, S., D. Bouzida, …, J. M. Rosenberg. 1992. The weighted histogram analysis method for free-energy calculations on biomolecules. I. The method. *J. Comput. Chem.* 13:1011–1021.

48. Hub, J. S., B. L. de Groot, and D. van der Spoel. 2010. g_wham—A free weighted histogram analysis implementation including robust error and autocorrelation estimates. *J. Chem. Theory Comput.* 6: 3713–3720.

49. Fiser, A., and A. Sali. 2003. Modeller: generation and refinement of homology-based protein structure models. *Methods Enzymol.* 374: 461–491.

50. Wolf, M. G., M. Hoefling, …, G. Groenhof. 2010. g_membed: Efficient insertion of a membrane protein into an equilibrated lipid bilayer with minimal perturbation. *J. Comput. Chem.* 31:2169–2174.

51. Trott, O., and A. J. Olson. 2010. AutoDock Vina: improving the speed and accuracy of docking with a new scoring function, efficient optimization, and multithreading. *J. Comput. Chem.* 31:455–461.

52. Daura, X., K. Gademann, …, A. E. Mark. 1999. Peptide folding: When simulation meets experiment. *Angew. Chem. Int.* 38:236–240.

53. Schrödinger, LLC. 2010. The {PyMOL} Molecular Graphics System, Version~1.3r1.

54. Humphrey, W., A. Dalke, and K. Schulten. 1996. VMD: visual molecular dynamics. *J. Mol. Graph.* 14:33–38, 27–28.

55. Pettersen, E. F., T. D. Goddard, …, T. E. Ferrin. 2004. UCSF Chimera—a visualization system for exploratory research and analysis. *J. Comput. Chem.* 25:1605–1612.

56. Tewari, Y. B., M. M. Miller, …, D. E. Martire. 1982. Aqueous solubility and octanol/water partition coefficient of organic compounds at 25.0. *J. Chem. Eng. Data.* 27:451–454.

57. Iida, T., T. Moriyama, …, M. Tominaga. 2003. TRPV1 activation and induction of nociceptive response by a non-pungent capsaicin-like compound, capsiate. *Neuropharmacology.* 44:958–967.

58. Romo, T. D., and A. Grossfield. 2014. Unknown unknowns: the challenge of systematic and statistical error in molecular dynamics simulations. *Biophys. J.* 106:1553–1554.

59. Martin, L. J., R. Chao, and B. Corry. 2014. Molecular dynamics simulation of the partitioning of benzocaine and phenytoin into a lipid bilayer. *Biophys. Chem.* 185:98–107.

60. Mokrab, Y., and M. S. P. Sansom. 2011. Interaction of diverse voltage sensor homologs with lipid bilayers revealed by self-assembly simulations. *Biophys. J.* 100:875–884.

61. Sands, Z. A., and M. S. P. Sansom. 2007. How does a voltage sensor interact with a lipid bilayer? Simulations of a potassium channel domain. *Structure.* 15:235–244.

62. Guan, J.-Y., J. M. Foerster, …, M. Ubbink. 2014. An ensemble of rapidly interconverting orientations in electrostatic protein-peptide complexes characterized by NMR spectroscopy. *ChemBioChem.* 15: 556–566.

63. Stouffer, A. L., R. Acharya, …, W. F. DeGrado. 2008. Structural basis for the function and inhibition of an influenza virus proton channel. *Nature.* 451:596–599.

64. Schnell, J. R., and J. J. Chou. 2008. Structure and mechanism of the M2 proton channel of influenza A virus. *Nature.* 451:591–595.

65. Paloncýová, M., R. DeVane, …, M. Otyepka. 2014. Amphiphilic drug-like molecules accumulate in a membrane below the head group region. *J. Phys. Chem. B.* 118:1030–1039.

66. Coskun, U., and K. Simons. 2011. Cell membranes: the lipid perspective. *Structure.* 19:1543–1548.

67. Senning, E. N., M. D. Collins, …, S. E. Gordon. 2014. Regulation of TRPV1 ion channel by phosphoinositide (4,5)-bisphosphate: the role of membrane asymmetry. *J. Biol. Chem.* 289:10999–11006.

68. Limongelli, V., M. Bonomi, and M. Parrinello. 2013. Funnel metadynamics as accurate binding free-energy method. *Proc. Natl. Acad. Sci. USA.* 110:6358–6363.

Supporting Material for:

Capsaicin Interaction with TRPV1 Channels in a Lipid Bilayer: Molecular Dynamics Simulation

Sonya M. Hanson, Simon Newstead, Kenton J. Swartz, & Mark S.P. Sansom

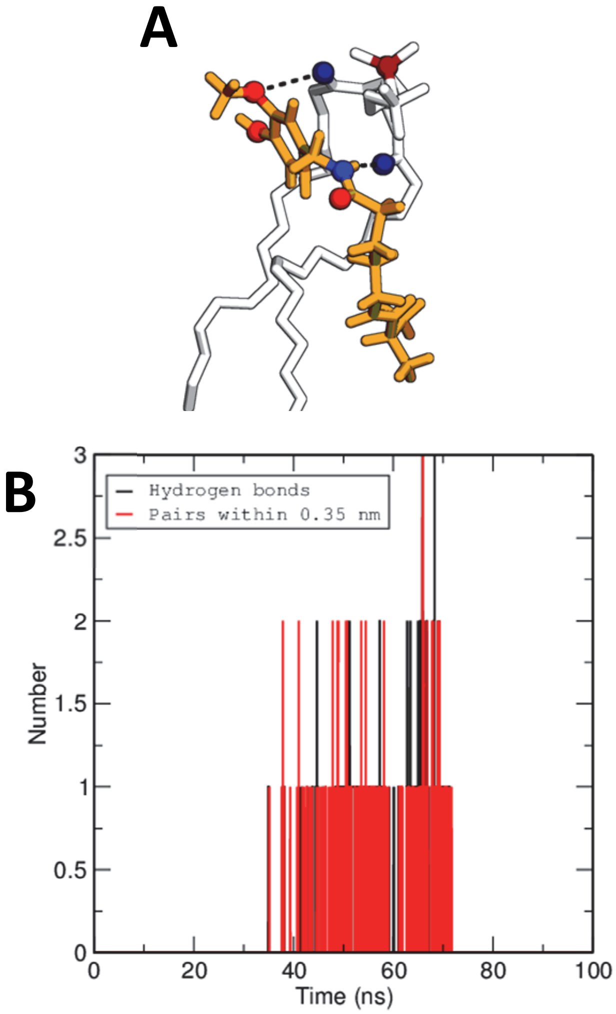

FIGURE S1 **POPC and Capsaicin hydrogen bonding interaction.** *(A)* An example of a long-lived hydrogen bonding interaction between a carbonyl group of a POPC molecule (in white) and the B region of capsaicin (in orange). *(B)* Number of H-bonds vs. time between capsaicin and the selected POPC molecule. This interaction is maintained for more than 30 ns of the simulation.

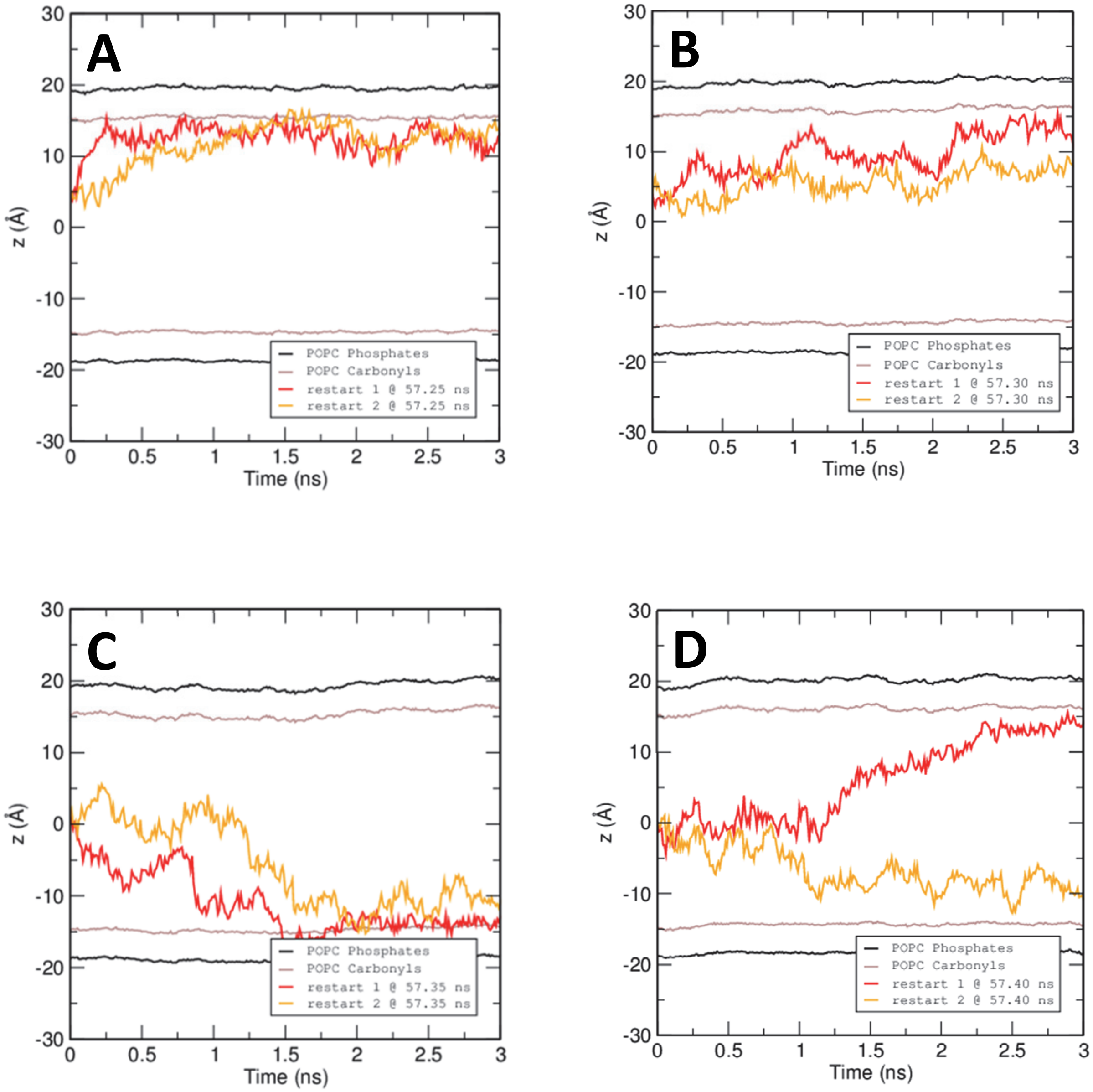

FIGURE S2 **Multiple simulations initiated at selected points along the original capsaicin flip-flop trajectory.** *(A)* Shows two repeats (red and orange lines) with different random velocity seeds using a starting position at 57.25 ns, *(B)* at 57.30 ns, *(C)* at 57.35 ns, and *(D)* at 57.40 ns along the original trajectory which yielded a flip-flop event. Red or orange lines represent the center of mass of capsaicin during different repeats. Black lines represent the phosphate head groups of POPC. Brown lines represent the carbonyls of POPC.

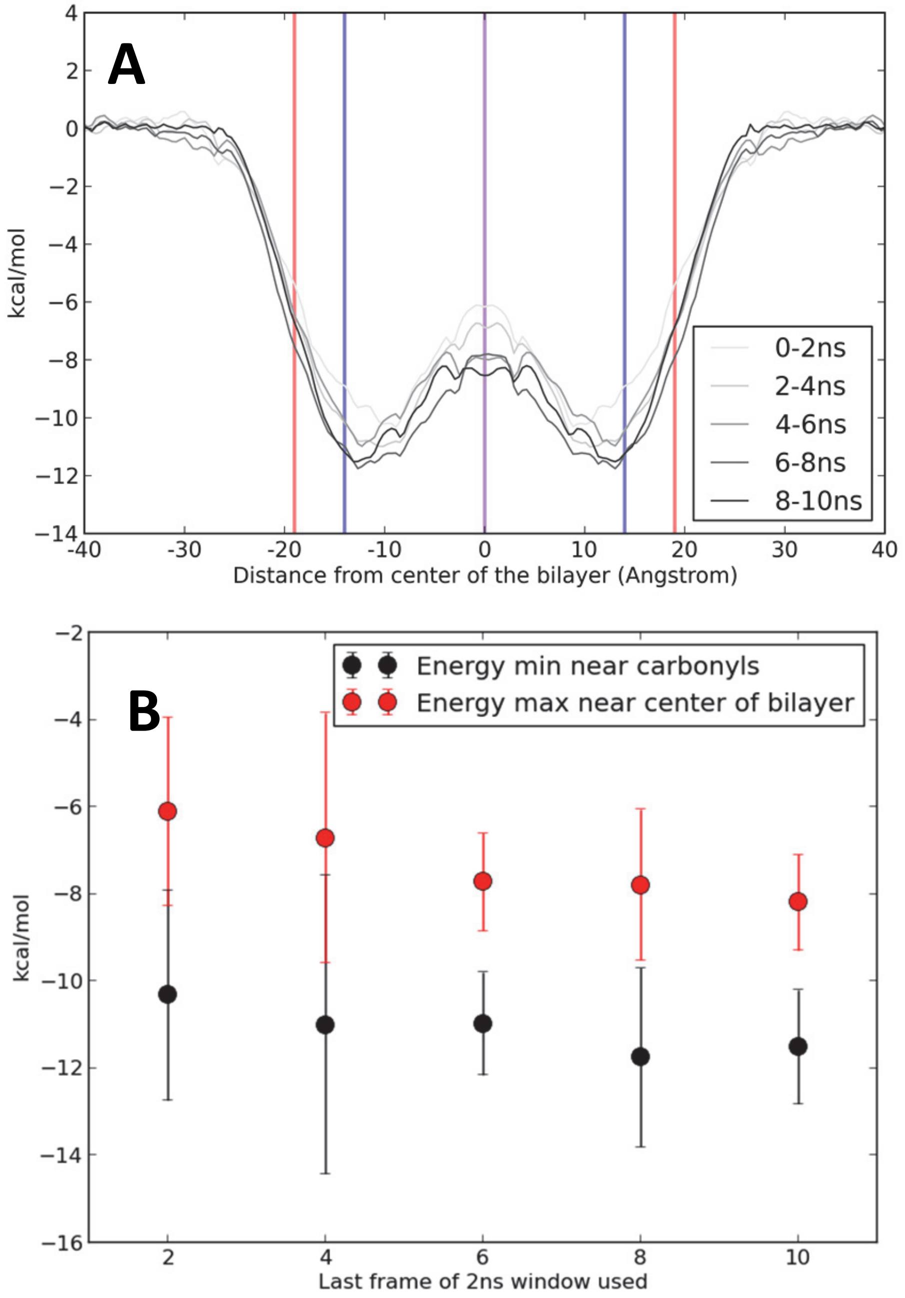

FIGURE S3 **PMF Convergence.** *(A)* PMF profiles as calculated from consecutive 2 ns segments of the full 10 ns simulations of 1 Å windows across the bilayer. *(B)* The values of the energy minima near the carbonyls (z = ±12 Å) and the energy maxima near the center of the bilayer (z = 0 Å) were calculated for the consecutive PMFs in *A* to provide a measure of convergence of the free energy profile.

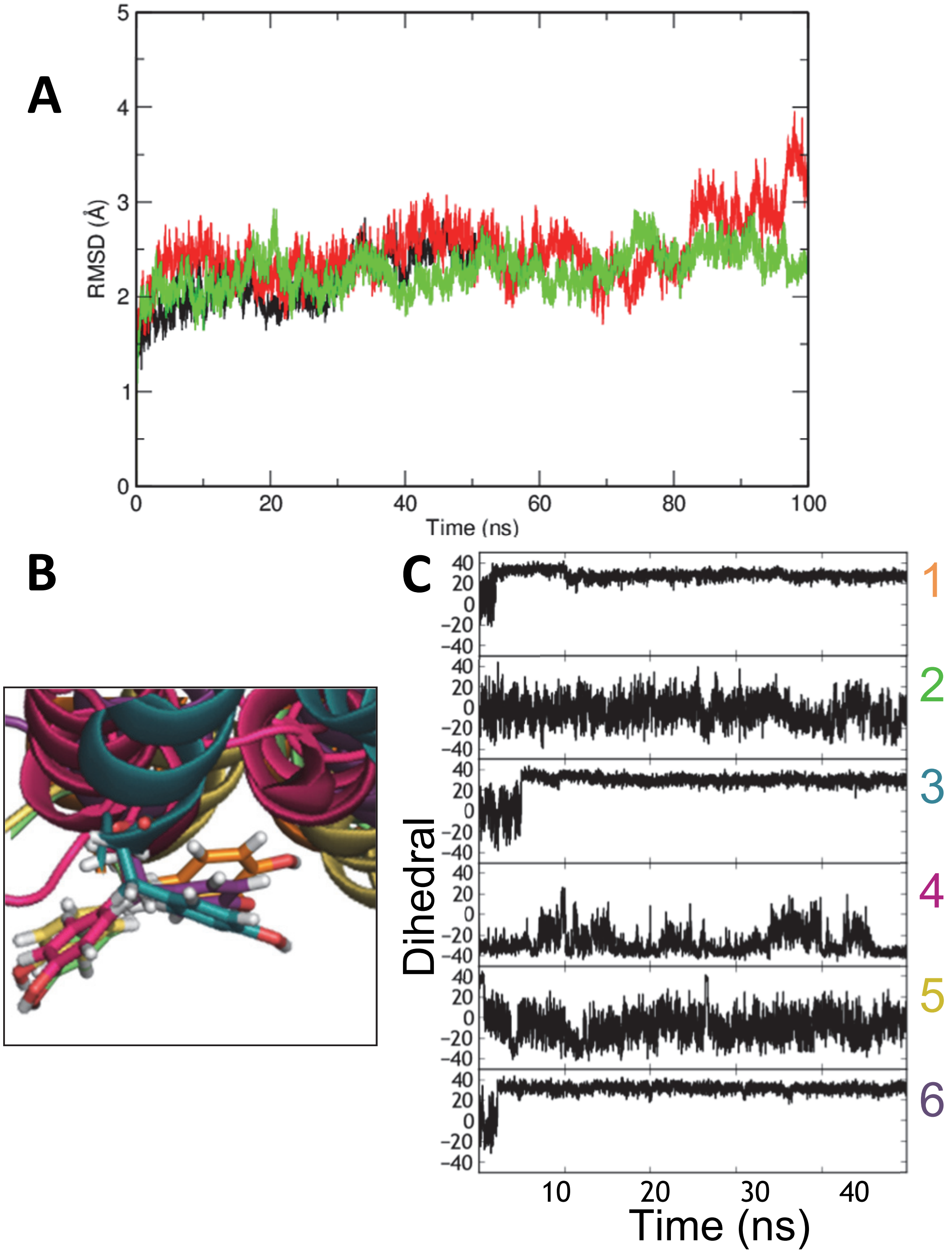

FIGURE S4 **Analysis of stability of the TRPV1 S1-S4 in model membrane.** *(A)* The backbone RMSD from the initial structure of the S1-S4 domain of TRPV1 over the course of three repeat 100 ns simulations. *(B)* The flip from an outward to an inward conformation of the sidechain of tyrosine 511. *(C)* Changes in the X_1 dihedral angle of Tyr511 over time of six separate simulations (colored accordingly) of the S1-S4 of the apo EM structure in a POPC bilayer.

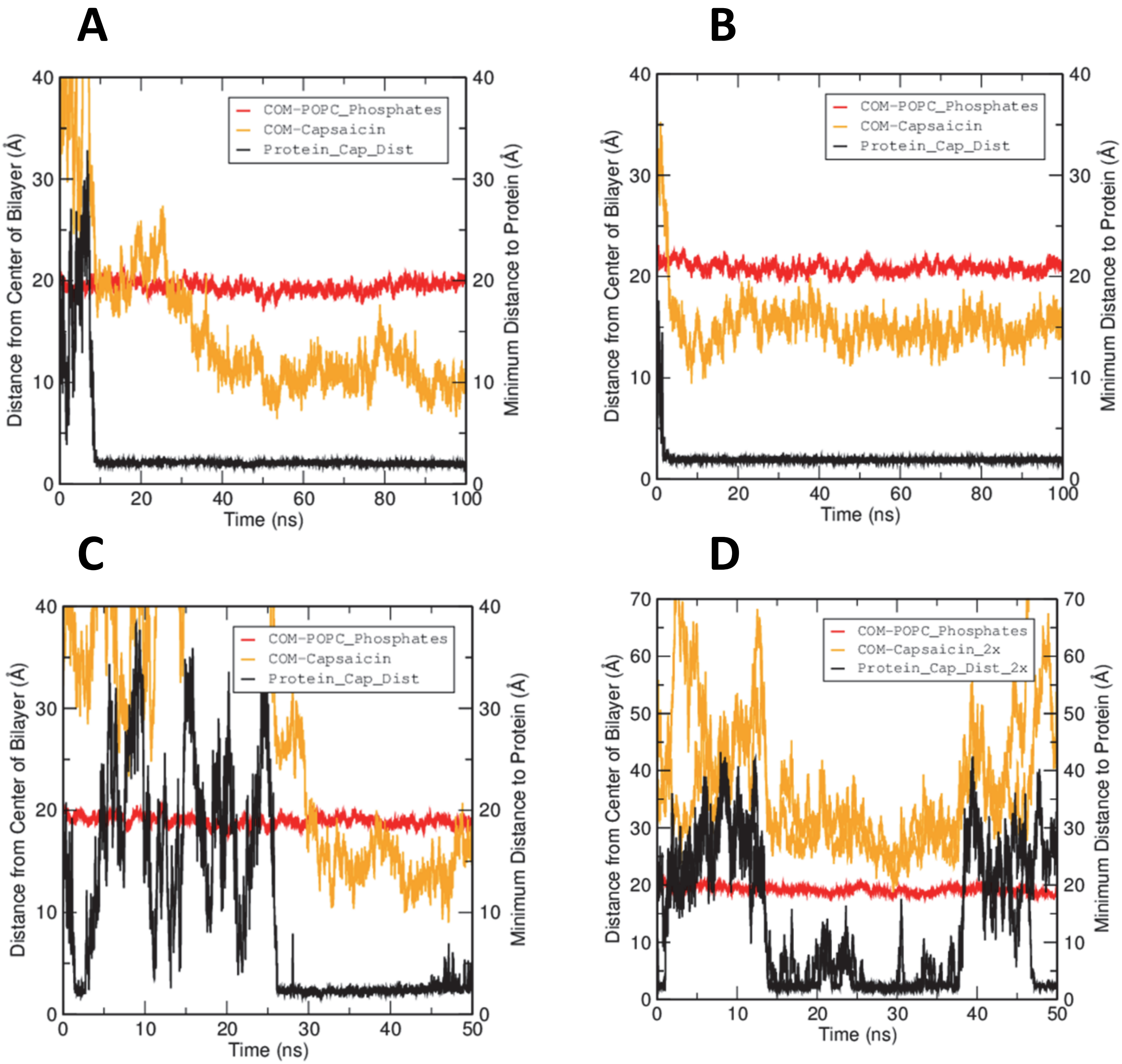

FIGURE S5 **Capsaicin interaction with TRPV1 S1-S4 compared to its membrane penetration.** Plots of the minimum distance of interacting capsaicin molecules to the TRPV1 S1-S4 domain over the course of four simulations (black), overlayed with the bilayer penetration as shown by the center of mass of capsaicin (orange) relative to that of the phosphates of the intracellular leaflet (red). This is shown for two capsaicin molecules that interact with the S1 helix *(A)* and *(B)*, and for three capsaicin molecules that interact with the S2-S3 loop *(C)* and *(D)*. *(D)* is an interaction between a two-capsaicin aggregate and the TRPV1 S1-S4. Notice how in A-C the capsaicin interacts with the protein before and during bilayer penetration.

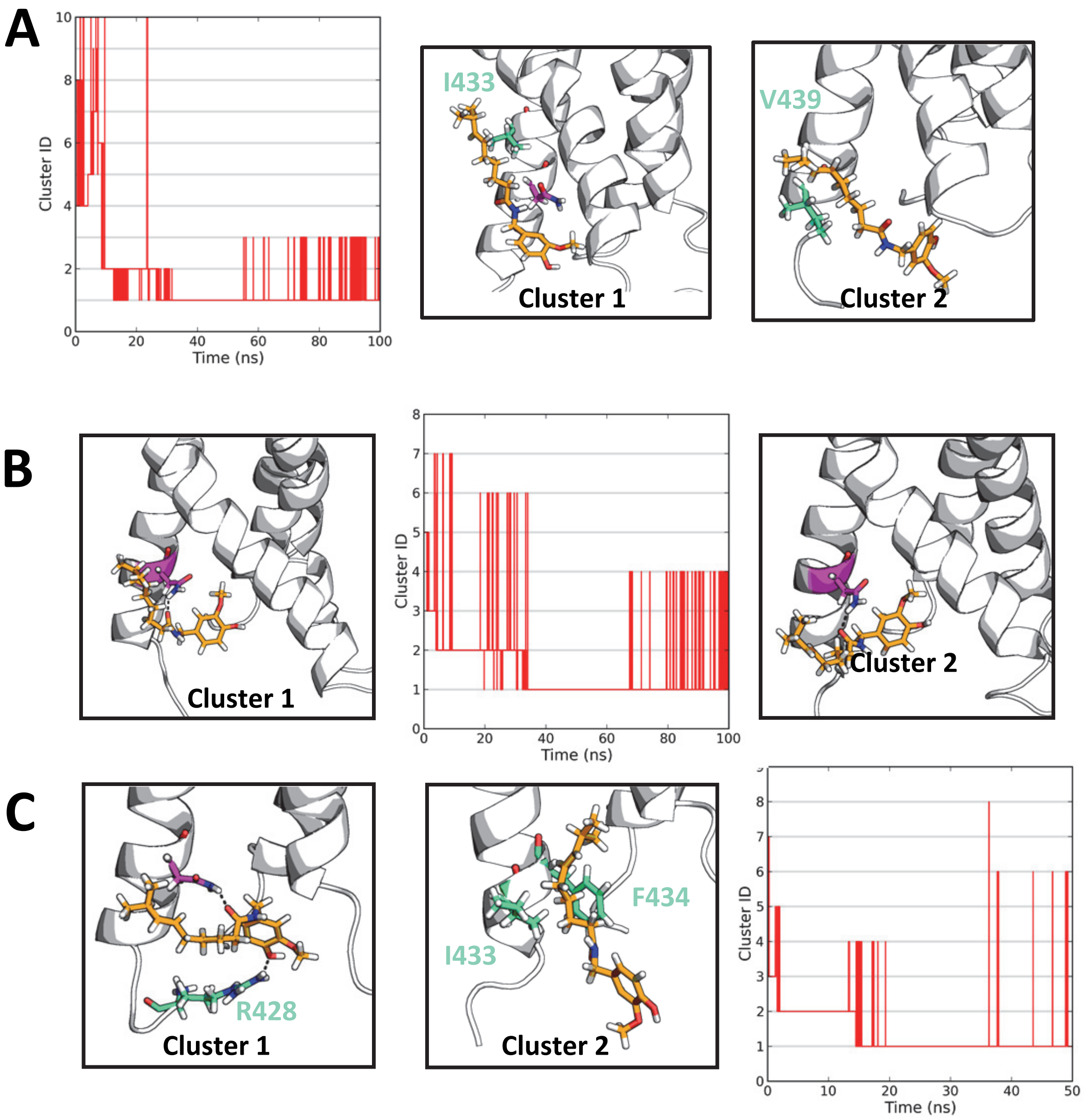

FIGURE S6 **Results of cluster analysis for 'encounter complex' simulations in which interactions are seen between capsaicin and the S1 helix.** *(A)* Cluster analysis of one 100 ns simulation results in a final cluster that involves N437 (magenta), but also I433 (teal). In the second-most dominant cluster the interaction with N437 is not seen, and V439 (teal) instead is seen to interact with capsaicin, perhaps on it's way to a more stable pose. *(B)* This 100 ns simulation is also the one shown in Fig. *7*. Cluster analysis shows a dominant cluster in which capsaicin interacts with N437 (magenta). The second-most dominant cluster also shares this interaction, but the C group acyl chain tail is in a different orientation. *(C)* In this 50 ns simulation, an interaction between capsaicin and the S1 helix is also seen, and the N437 (magenta) interaction is once again observed in the most dominant cluster. However, the R428 (teal) interaction is also seen. This interaction may be an artifact of the S1-S4 helices being truncated from the rest of the full length protein, although it is not seen in the other two simulations.

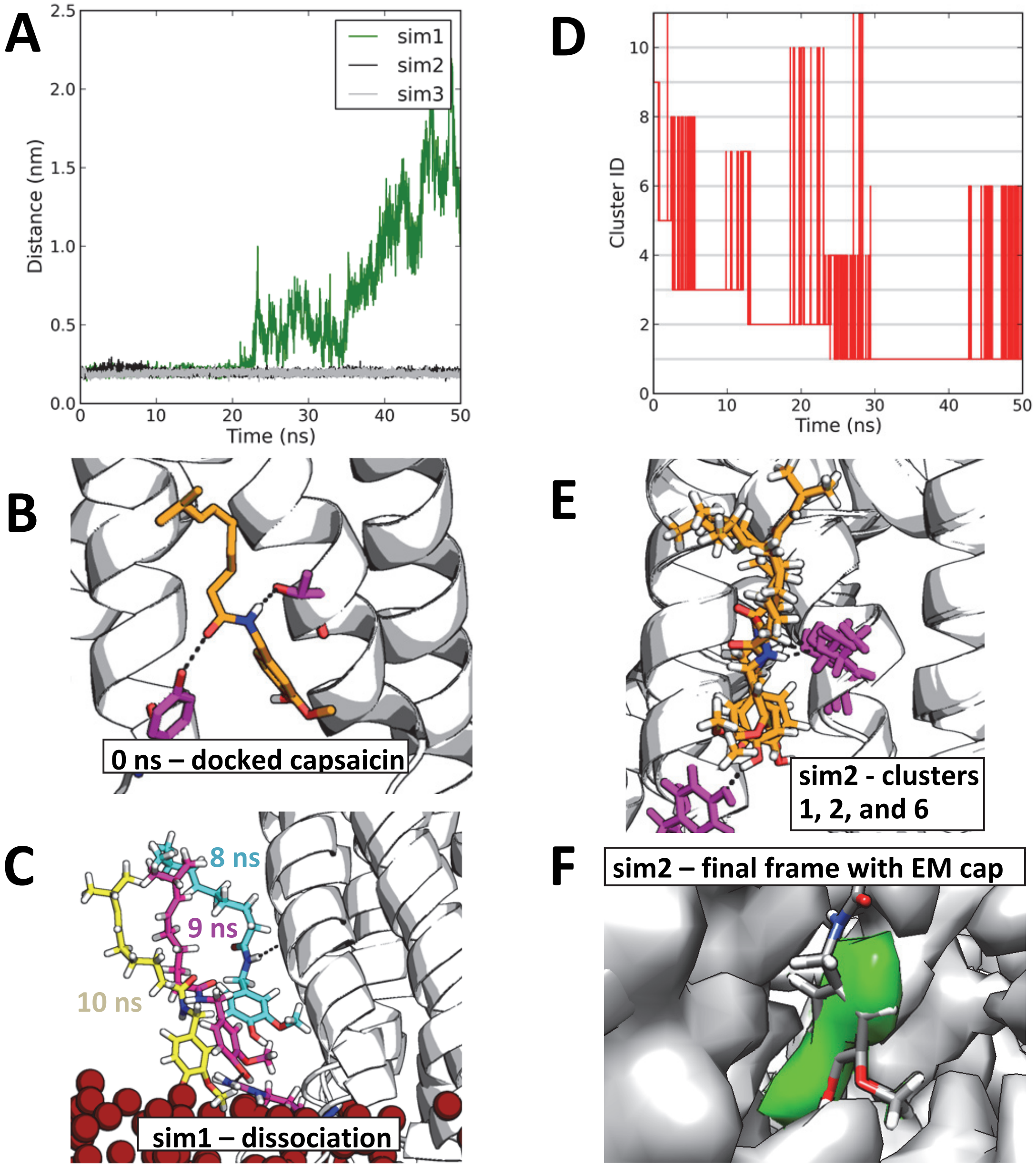

FIGURE S7 **Results of 50ns simulation of capsaicin docked to TRPV1.** *(A)* In two out of three simulations capsaicin continued to equilibrate within the docked position between the S3 and S4. *(B)* The docked pose of capsaicin was the top-ranked pose found using the Autodock Vina program and the full-length TRPV1 (PDB ID: 3J5R). T550 and Y511 are shown in magenta. *(C)* In the simulation in which capsaicin dissociated from the S1-S4 helices it initially had formed a hydrogen bond with T550 (cyan) and then began to dissociate, interacting with an arginine at the intracellular side of the S4 (magenta) and then finally with only the POPC molecules (yellow) in a position seen in the capsaicin-bilayer only simulations. *(D)* Cluster analysis of one of the simulations in which capsaicin remained in the docked pose shows small shifts toward a final cluster. *(E)* Three of the dominant clusters from D (1, 2, and 6) are shown. A change from the initial docked pose is that now Y511 forms a hydrogen bonding interaction with the A group aromatic region instead of the B group. *(F)* An overlay of the final frame of this simulation shows that the A group of capsaicin overlaps with density suspected to correspond to capsaicin in the sharpened EM density (deep green) of the capsaicin bound structure (temperature factor -200 Å, filtered to 4.2 Å, shown at sigma level 8).

Supporting Material - Parameter (*.itp) file for capsaicin

```
 [ moleculetype ]
; name  nrexcl
CAP   3

 [ atoms ]
;  nr    type   resnr   residu   atom   cgnr    charge      mass
    1    opls_145     1       CAP      C1    1  -0.11500  12.01100       ;  -0.1150000
    2    opls_146     1       CAP     H12    1   0.11500   1.00800       ;   0.0000000
    3    opls_145     1       CAP      C2    2  -0.11500  12.01100       ;  -0.1150000
    4    opls_145     1       CAP      C3    3  -0.11500  12.01100       ;  -0.2300000
    5    opls_146     1       CAP     H13    3   0.11500   1.00800       ;  -0.1150000
    6    opls_145     1       CAP      C4    4  -0.11500  12.01100       ;  -0.2300000
    7    opls_146     1       CAP     H14    4   0.11500   1.00800       ;  -0.1150000
    8    opls_166     1       CAP      C5    5   0.15000  12.01100       ;   0.0350000
    9    opls_166     1       CAP      C6    6   0.15000  12.01100       ;   0.1850000
   10    opls_179     1       CAP      O1    6  -0.28500  15.99940       ;  -0.1000000
   11    opls_157     1       CAP      C7    7   0.13000  12.01100       ;   0.0450000
   12    opls_156     1       CAP     H17    7   0.04000   1.00800       ;   0.0850000
   13    opls_156     1       CAP     H18    7   0.04000   1.00800       ;   0.1250000
   14    opls_156     1       CAP     H19    7   0.04000   1.00800       ;   0.1650000
   15    opls_167     1       CAP      O2    5  -0.58500  15.99940       ;  -0.4200000
   16    opls_168     1       CAP     H20    5   0.43500   1.00800       ;   0.0150000
   17    opls_244     1       CAP      C8    8   0.08000  12.01100       ;   0.0950000
   18    opls_140     1       CAP     H10    8   0.06000   1.00800       ;   0.1550000
   19    opls_140     1       CAP     H11    8   0.06000   1.00800       ;   0.2150000
   20    opls_238     1       CAP      N1    8  -0.50000  14.00670       ;  -0.2850000
   21    opls_241     1       CAP     H21    8   0.30000   1.00800       ;   0.0150000
   22    opls_235     1       CAP      C9    9   0.50000  12.01100       ;   0.5150000
   23    opls_136     1       CAP     C10   10  -0.12000  12.01100       ;   0.3950000
   24    opls_140     1       CAP      H8   10   0.06000   1.00800       ;   0.4550000
   25    opls_140     1       CAP      H9   10   0.06000   1.00800       ;   0.5150000
   26    opls_236     1       CAP      O3    9  -0.50000  15.99940       ;   0.0150000
   27    opls_136     1       CAP     C11   11  -0.12000  12.01100       ;  -0.1050000
   28    opls_140     1       CAP      H7   11   0.06000   1.00800       ;  -0.0450000
   29    opls_140     1       CAP     H16   11   0.06000   1.00800       ;   0.0150000
   30    opls_136     1       CAP     C12   12  -0.12000  12.01100       ;  -0.1050000
   31    opls_140     1       CAP      H5   12   0.06000   1.00800       ;  -0.0450000
   32    opls_140     1       CAP      H6   12   0.06000   1.00800       ;   0.0150000
   33    opls_136     1       CAP     C13   13  -0.12000  12.01100       ;  -0.1050000
   34    opls_140     1       CAP      H4   13   0.06000   1.00800       ;  -0.0450000
   35    opls_140     1       CAP     H15   13   0.06000   1.00800       ;   0.0150000
   36    opls_142     1       CAP     C14   14  -0.11500  12.01100       ;  -0.1000000
   37    opls_144     1       CAP      H3   14   0.11500   1.00800       ;   0.0150000
   38    opls_142     1       CAP     C15   15  -0.11500  12.01100       ;  -0.1000000
   39    opls_144     1       CAP      H1   15   0.11500   1.00800       ;   0.0150000
   40    opls_137     1       CAP     C16   16  -0.06000  12.01100       ;  -0.0450000
   41    opls_140     1       CAP      H2   16   0.06000   1.00800       ;   0.0150000
   42    opls_135     1       CAP     C17   17  -0.18000  12.01100       ;  -0.1650000
   43    opls_140     1       CAP     H25   17   0.06000   1.00800       ;  -0.1050000
   44    opls_140     1       CAP     H26   17   0.06000   1.00800       ;  -0.0450000
   45    opls_140     1       CAP     H27   17   0.06000   1.00800       ;   0.0150000
   46    opls_135     1       CAP     C18   18  -0.18000  12.01100       ;  -0.1650000
   47    opls_140     1       CAP     H22   18   0.06000   1.00800       ;  -0.1050000
   48    opls_140     1       CAP     H23   18   0.06000   1.00800       ;  -0.0450000
   49    opls_140     1       CAP     H24   18   0.06000   1.00800       ;   0.0150000
; total molecule charge =   0.0000000


 [ bonds ]
;   ai  aj   funct      b0          kb
      1     3   1     0.14000      392459.       ;    C1-    C2
      1     9   1     0.14000      392459.       ;    C1-    C6
      3     4   1     0.14000      392459.       ;    C2-    C3
```

```
     4     6   1     0.14000      392459.       ;    C3-    C4
     6     8   1     0.14000      392459.       ;    C4-    C5
     8     9   1     0.14000      392459.       ;    C5-    C6
     9    10   1     0.13640      376560.       ;    C6-    O1
    10    11   1     0.14100      267776.       ;    O1-    C7
     8    15   1     0.13640      376560.       ;    C5-    O2
     3    17   1     0.15100      265266.       ;    C2-    C8
    17    20   1     0.14490      282002.       ;    C8-    N1
    20    22   1     0.13350      410032.       ;    N1-    C9
    22    23   1     0.15220      265266.       ;    C9-   C10
    22    26   1     0.12290      476976.       ;    C9-    O3
    23    27   1     0.15290      224262.       ;   C10-   C11
    27    30   1     0.15290      224262.       ;   C11-   C12
    30    33   1     0.15290      224262.       ;   C12-   C13
    33    36   1     0.15100      265266.       ;   C13-   C14
    36    38   1     0.13400      459403.       ;   C14-   C15
    38    40   1     0.15100      265266.       ;   C15-   C16
    40    42   1     0.15290      224262.       ;   C16-   C17
    40    46   1     0.15290      224262.       ;   C16-   C18
    38    39   1     0.10800      307106.       ;   C15-    H1
    40    41   1     0.10900      284512.       ;   C16-    H2
    36    37   1     0.10800      307106.       ;   C14-    H3
    33    34   1     0.10900      284512.       ;   C13-    H4
    30    31   1     0.10900      284512.       ;   C12-    H5
    30    32   1     0.10900      284512.       ;   C12-    H6
    27    28   1     0.10900      284512.       ;   C11-    H7
    23    24   1     0.10900      284512.       ;   C10-    H8
    23    25   1     0.10900      284512.       ;   C10-    H9
    17    18   1     0.10900      284512.       ;    C8-   H10
    17    19   1     0.10900      284512.       ;    C8-   H11
     1     2   1     0.10800      307106.       ;    C1-   H12
     4     5   1     0.10800      307106.       ;    C3-   H13
     6     7   1     0.10800      307106.       ;    C4-   H14
    33    35   1     0.10900      284512.       ;   C13-   H15
    27    29   1     0.10900      284512.       ;   C11-   H16
    11    12   1     0.10900      284512.       ;    C7-   H17
    11    13   1     0.10900      284512.       ;    C7-   H18
    11    14   1     0.10900      284512.       ;    C7-   H19
    15    16   1     0.09450      462750.       ;    O2-   H20
    20    21   1     0.10100      363171.       ;    N1-   H21
    46    47   1     0.10900      284512.       ;   C18-   H22
    46    48   1     0.10900      284512.       ;   C18-   H23
    46    49   1     0.10900      284512.       ;   C18-   H24
    42    43   1     0.10900      284512.       ;   C17-   H25
    42    44   1     0.10900      284512.       ;   C17-   H26
    42    45   1     0.10900      284512.       ;   C17-   H27


[ constraints ]
     8      16   2     0.19393       ;    C5     H20

[ pairs ]
     9       4  1       ;    C6-    C3
     9      17  1       ;    C6-    C8
     2       4  1       ;   H12-    C3
     2      17  1       ;   H12-    C8
     3       8  1       ;    C2-    C5
     3      10  1       ;    C2-    O1
     2       8  1       ;   H12-    C5
     2      10  1       ;   H12-    O1
     1       6  1       ;    C1-    C4
     1       5  1       ;    C1-   H13
    17       6  1       ;    C8-    C4
    17       5  1       ;    C8-   H13
     3       7  1       ;    C2-   H14
```

5	8	1	;	H13-	C5
5	7	1	;	H13-	H14
4	15	1	;	C3-	O2
7	9	1	;	H14-	C6
7	15	1	;	H14-	O2
6	10	1	;	C4-	O1
15	1	1	;	O2-	C1
15	10	1	;	O2-	O1
1	11	1	;	C1-	C7
8	11	1	;	C5-	C7
9	12	1	;	C6-	H17
9	13	1	;	C6-	H18
9	14	1	;	C6-	H19
6	16	1	;	C4-	H20
9	16	1	;	C6-	H20
1	20	1	;	C1-	N1
1	18	1	;	C1-	H10
1	19	1	;	C1-	H11
4	20	1	;	C3-	N1
4	18	1	;	C3-	H10
4	19	1	;	C3-	H11
3	22	1	;	C2-	C9
3	21	1	;	C2-	H21
18	22	1	;	H10-	C9
18	21	1	;	H10-	H21
19	22	1	;	H11-	C9
19	21	1	;	H11-	H21
17	23	1	;	C8-	C10
17	26	1	;	C8-	O3
21	23	1	;	H21-	C10
21	26	1	;	H21-	O3
20	27	1	;	N1-	C11
20	24	1	;	N1-	H8
20	25	1	;	N1-	H9
26	27	1	;	O3-	C11
26	24	1	;	O3-	H8
26	25	1	;	O3-	H9
22	30	1	;	C9-	C12
22	28	1	;	C9-	H7
22	29	1	;	C9-	H16
24	30	1	;	H8-	C12
24	28	1	;	H8-	H7
24	29	1	;	H8-	H16
25	30	1	;	H9-	C12
25	28	1	;	H9-	H7
25	29	1	;	H9-	H16
23	33	1	;	C10-	C13
23	31	1	;	C10-	H5
23	32	1	;	C10-	H6
28	33	1	;	H7-	C13
28	31	1	;	H7-	H5
28	32	1	;	H7-	H6
29	33	1	;	H16-	C13
29	31	1	;	H16-	H5
29	32	1	;	H16-	H6
27	36	1	;	C11-	C14
27	34	1	;	C11-	H4
27	35	1	;	C11-	H15
31	36	1	;	H5-	C14
31	34	1	;	H5-	H4
31	35	1	;	H5-	H15
32	36	1	;	H6-	C14
32	34	1	;	H6-	H4
32	35	1	;	H6-	H15
30	38	1	;	C12-	C15

```
      30      37  1       ;   C12-    H3
      34      38  1       ;    H4-   C15
      34      37  1       ;    H4-    H3
      35      38  1       ;   H15-   C15
      35      37  1       ;   H15-    H3
      33      40  1       ;   C13-   C16
      33      39  1       ;   C13-    H1
      37      40  1       ;    H3-   C16
      37      39  1       ;    H3-    H1
      36      42  1       ;   C14-   C17
      36      46  1       ;   C14-   C18
      36      41  1       ;   C14-    H2
      39      42  1       ;    H1-   C17
      39      46  1       ;    H1-   C18
      39      41  1       ;    H1-    H2
      38      43  1       ;   C15-   H25
      38      44  1       ;   C15-   H26
      38      45  1       ;   C15-   H27
      46      43  1       ;   C18-   H25
      46      44  1       ;   C18-   H26
      46      45  1       ;   C18-   H27
      41      43  1       ;    H2-   H25
      41      44  1       ;    H2-   H26
      41      45  1       ;    H2-   H27
      38      47  1       ;   C15-   H22
      38      48  1       ;   C15-   H23
      38      49  1       ;   C15-   H24
      42      47  1       ;   C17-   H22
      42      48  1       ;   C17-   H23
      42      49  1       ;   C17-   H24
      41      47  1       ;    H2-   H22
      41      48  1       ;    H2-   H23
      41      49  1       ;    H2-   H24

[ angles ]
; ai  aj  ak  funct      th0         cth
     9     1     3  1     120.000    527.1840     ;    C6-     C1-     C2
     2     1     3  1     120.000    292.8800     ;   H12-     C1-     C2
     1     3     4  1     120.000    527.1840     ;    C1-     C2-     C3
     1     3    17  1     120.000    585.7600     ;    C1-     C2-     C8
     2     1     9  1     120.000    292.8800     ;   H12-     C1-     C6
     1     9     8  1     120.000    527.1840     ;    C1-     C6-     C5
     1     9    10  1     120.000    585.7600     ;    C1-     C6-     O1
    17     3     4  1     120.000    585.7600     ;    C8-     C2-     C3
     3     4     6  1     120.000    527.1840     ;    C2-     C3-     C4
     3     4     5  1     120.000    292.8800     ;    C2-     C3-    H13
     5     4     6  1     120.000    292.8800     ;   H13-     C3-     C4
     4     6     8  1     120.000    527.1840     ;    C3-     C4-     C5
     4     6     7  1     120.000    292.8800     ;    C3-     C4-    H14
     7     6     8  1     120.000    292.8800     ;   H14-     C4-     C5
     6     8     9  1     120.000    527.1840     ;    C4-     C5-     C6
     6     8    15  1     120.000    585.7600     ;    C4-     C5-     O2
    15     8     9  1     120.000    585.7600     ;    O2-     C5-     C6
     8     9    10  1     120.000    585.7600     ;    C5-     C6-     O1
     9    10    11  1     111.000    627.6000     ;    C6-     O1-     C7
    10    11    12  1     109.500    292.8800     ;    O1-     C7-    H17
    10    11    13  1     109.500    292.8800     ;    O1-     C7-    H18
    10    11    14  1     109.500    292.8800     ;    O1-     C7-    H19
     8    15    16  1     113.000    292.8800     ;    C5-     O2-    H20
     3    17    20  1     109.700    669.4400     ;    C2-     C8-     N1
     3    17    18  1     109.500    292.8800     ;    C2-     C8-    H10
     3    17    19  1     109.500    292.8800     ;    C2-     C8-    H11
    18    17    20  1     109.500    292.8800     ;   H10-     C8-     N1
    19    17    20  1     109.500    292.8800     ;   H11-     C8-     N1
    17    20    22  1     121.900    418.4000     ;    C8-     N1-     C9
```

17	20	21	1	118.400	317.9840	;	C8-	N1-	H21
21	20	22	1	119.800	292.8800	;	H21-	N1-	C9
20	22	23	1	116.600	585.7600	;	N1-	C9-	C10
20	22	26	1	122.900	669.4400	;	N1-	C9-	O3
26	22	23	1	120.400	669.4400	;	O3-	C9-	C10
22	23	27	1	111.100	527.1840	;	C9-	C10-	C11
22	23	24	1	109.500	292.8800	;	C9-	C10-	H8
22	23	25	1	109.500	292.8800	;	C9-	C10-	H9
24	23	27	1	110.700	313.8000	;	H8-	C10-	C11
25	23	27	1	110.700	313.8000	;	H9-	C10-	C11
23	27	30	1	112.700	488.2730	;	C10-	C11-	C12
23	27	28	1	110.700	313.8000	;	C10-	C11-	H7
23	27	29	1	110.700	313.8000	;	C10-	C11-	H16
28	27	30	1	110.700	313.8000	;	H7-	C11-	C12
29	27	30	1	110.700	313.8000	;	H16-	C11-	C12
27	30	33	1	112.700	488.2730	;	C11-	C12-	C13
27	30	31	1	110.700	313.8000	;	C11-	C12-	H5
27	30	32	1	110.700	313.8000	;	C11-	C12-	H6
31	30	33	1	110.700	313.8000	;	H5-	C12-	C13
32	30	33	1	110.700	313.8000	;	H6-	C12-	C13
30	33	36	1	111.100	527.1840	;	C12-	C13-	C14
30	33	34	1	110.700	313.8000	;	C12-	C13-	H4
30	33	35	1	110.700	313.8000	;	C12-	C13-	H15
34	33	36	1	109.500	292.8800	;	H4-	C13-	C14
35	33	36	1	109.500	292.8800	;	H15-	C13-	C14
33	36	38	1	124.000	585.7600	;	C13-	C14-	C15
33	36	37	1	117.000	292.8800	;	filled		
37	36	38	1	120.000	292.8800	;	H3-	C14-	C15
36	38	40	1	124.000	585.7600	;	C14-	C15-	C16
36	38	39	1	120.000	292.8800	;	C14-	C15-	H1
39	38	40	1	117.000	292.8800	;	filled		
38	40	42	1	111.100	527.1840	;	C15-	C16-	C17
38	40	46	1	111.100	527.1840	;	C15-	C16-	C18
38	40	41	1	109.500	292.8800	;	C15-	C16-	H2
46	40	42	1	112.700	488.2730	;	C18-	C16-	C17
41	40	42	1	110.700	313.8000	;	H2-	C16-	C17
40	42	43	1	110.700	313.8000	;	C16-	C17-	H25
40	42	44	1	110.700	313.8000	;	C16-	C17-	H26
40	42	45	1	110.700	313.8000	;	C16-	C17-	H27
41	40	46	1	110.700	313.8000	;	H2-	C16-	C18
40	46	47	1	110.700	313.8000	;	C16-	C18-	H22
40	46	48	1	110.700	313.8000	;	C16-	C18-	H23
40	46	49	1	110.700	313.8000	;	C16-	C18-	H24
35	33	34	1	107.800	276.1440	;	H15-	C13-	H4
32	30	31	1	107.800	276.1440	;	H6-	C12-	H5
29	27	28	1	107.800	276.1440	;	H16-	C11-	H7
25	23	24	1	107.800	276.1440	;	H9-	C10-	H8
19	17	18	1	107.800	276.1440	;	H11-	C8-	H10
13	11	12	1	107.800	276.1440	;	H18-	C7-	H17
14	11	12	1	107.800	276.1440	;	H19-	C7-	H17
14	11	13	1	107.800	276.1440	;	H19-	C7-	H18
48	46	47	1	107.800	276.1440	;	H23-	C18-	H22
49	46	47	1	107.800	276.1440	;	H24-	C18-	H22
49	46	48	1	107.800	276.1440	;	H24-	C18-	H23
44	42	43	1	107.800	276.1440	;	H26-	C17-	H25
45	42	43	1	107.800	276.1440	;	H27-	C17-	H25
45	42	44	1	107.800	276.1440	;	H27-	C17-	H26

```
[ dihedrals ]
;  ai    aj    ak    al funct           c0           c1           c2           c3           c4           c5
     9     1     3     4   3  30.33400   0.00000 -30.33400   0.00000   0.00000   0.00000 ; dih    C6-    C1-    C2-    C3
     3     1     9     8   3  30.33400   0.00000 -30.33400   0.00000   0.00000   0.00000 ; dih    C2-    C1-    C6-    C5
     1     3     4     6   3  30.33400   0.00000 -30.33400   0.00000   0.00000   0.00000 ; dih    C1-    C2-    C3-    C4
     3     4     6     8   3  30.33400   0.00000 -30.33400   0.00000   0.00000   0.00000 ; dih    C2-    C3-    C4-    C5
     4     6     8     9   3  30.33400   0.00000 -30.33400   0.00000   0.00000   0.00000 ; dih    C3-    C4-    C5-    C6
     6     8     9     1   3  30.33400   0.00000 -30.33400   0.00000   0.00000   0.00000 ; dih    C4-    C5-    C6-    C1
     1     9    10    11   3  12.55200   0.00000 -12.55200   0.00000   0.00000   0.00000 ; dih    C1-    C6-    O1-    C7
     9    10    11    12   3   1.58992   4.76976   0.00000  -6.35968   0.00000   0.00000 ; dih    C6-    O1-    C7-   H17
     6     8    15    16   3   7.03749   0.00000  -7.03749   0.00000   0.00000   0.00000 ; dih    C4-    C5-    O2-   H20
     1     3    17    20   3   0.00000   0.00000   0.00000   0.00000   0.00000   0.00000 ; dih    C1-    C2-    C8-    N1
     3    17    20    22   3  -4.70700   2.92044   1.78656   0.00000   0.00000   0.00000 ; dih    C2-    C8-    N1-    C9
    17    20    22    23   3  30.28798  -4.81160 -25.47638   0.00000   0.00000   0.00000 ; dih    C8-    N1-    C9-   C10
    20    22    23    27   3   4.83252  -7.65254   1.68196   1.13805   0.00000   0.00000 ; dih    N1-    C9-   C10-   C11
    22    23    27    30   3  -4.96013   6.28646   1.30959  -2.63592   0.00000   0.00000 ; dih    C9-   C10-   C11-   C12
    23    27    30    33   3   2.92880  -1.46440   0.20920  -1.67360   0.00000   0.00000 ; dih   C10-   C11-   C12-   C13
    27    30    33    36   3   2.92880  -1.46440   0.20920  -1.67360   0.00000   0.00000 ; dih   C11-   C12-   C13-   C14
    30    33    36    38   3   0.52719  -6.39734  -1.69452   7.56467   0.00000   0.00000 ; dih   C12-   C13-   C14-   C15
    33    36    38    40   3  58.57600   0.00000 -58.57600   0.00000   0.00000   0.00000 ; dih   C13-   C14-   C15-   C16
    36    38    40    42   3   0.52719  -6.39734  -1.69452   7.56467   0.00000   0.00000 ; dih   C14-   C15-   C16-   C17
    38    40    42    43   3   0.76567   2.29701   0.00000  -3.06269   0.00000   0.00000 ; dih   C15-   C16-   C17-   H25
    38    40    46    47   3   0.76567   2.29701   0.00000  -3.06269   0.00000   0.00000 ; dih   C15-   C16-   C18-   H22

[ dihedrals ]
; ai  aj   ak  al  funct    phi0          cp      mult
     1     3     9     2   1     180.000       4.602   2        ; imp     C1-    C2-    C6-   H12
     3     1     4    17   1     180.000       4.602   2        ; imp     C2-    C1-    C3-    C8
     4     3     6     5   1     180.000       4.602   2        ; imp     C3-    C2-    C4-   H13
     6     4     8     7   1     180.000       4.602   2        ; imp     C4-    C3-    C5-   H14
     8     6     9    15   1     180.000       4.602   2        ; imp     C5-    C4-    C6-    O2
     9     1     8    10   1     180.000       4.602   2        ; imp     C6-    C1-    C5-    O1
    22    20    23    26   1     180.000      43.932   2        ; imp     C9-    N1-   C10-    O3
    36    33    38    37   1     180.000      62.760   2        ; imp    C14-   C13-   C15-    H3
    38    36    40    39   1     180.000      62.760   2        ; imp    C15-   C14-   C16-    H1
```
